# Reduction‐Controlled Tunable Synthesis of Covellite (CuS) Nanoparticles from Water‐Soluble Single‐Source Precursors

**DOI:** 10.1002/smll.202514339

**Published:** 2026-03-18

**Authors:** Xiang Xu, Siqiao Huang, Desmond A. Koomson, Tannith‐Jade Cole, Yuanyang Xie, Jagodish C. Sarker, David Pugh, Cécile A. Dreiss, Graeme Hogarth

**Affiliations:** ^1^ Department of Chemistry King's College London London UK; ^2^ Institute of Pharmaceutical Sciences King's College London Franklin Wilkins Building London UK; ^3^ Department of Physics and London Centre for Nanotechnology King's College London London UK; ^4^ Department of Chemistry Jagannath University Dhaka Bangladesh

**Keywords:** covellite, dithiocarbamate, nanomaterials, reduction‐controlled, water‐soluble

## Abstract

The single source precursor (SSP) approach has been extensively used in preparing technologically important covellite (CuS) nanoparticles (NPs) for its high atom‐efficiency and facile control over NPs. However, current SSPs often require time‐consuming decomposition in organic solvents at temperatures typically above 100°C. Here, we propose amino acid‐derived copper(II)‐dithiocarbamate (DTC) complexes, as water‐soluble SSPs for low‐temperature hydrothermal decomposition. We demonstrate that Cu(II)‐DTCs follow a reduction‐mediated molecular decomposition pathway to CuS, with the Cu(I) intermediates, Cu(I)‐DTCs, isolated and confirmed via formation of crystallographically‐characterized phosphine adducts. The Cu(II)‐Cu(I) reduction occurs via the intramolecular ligand‐to‐metal charge‐transfer (LMCT), which proves rate‐limiting in the decomposition and sensitive to changes in pH and ligand metalation. Tuning the rate of intramolecular LMCT and concomitant formation of Cu(I)‐DTC species by varying pH and metalation, monitored by UV–vis spectroscopy, allows the formation rate of molecular building blocks to be controlled, in turn controlling nucleation and growth of NPs. Thus, the same copper‐ligand combinations can be utilized to generate a wide range of size‐shape tunable CuS NPs (ca. 10–150 nm) cost‐effectively and sustainably. These results not only highlight the synthetic utility of this approach but also provide mechanistic insights into the poorly defined molecules‐to‐materials transformation.

## Graphical abstract

Amino acid‐derived Cu(II)‐dithiocarbamates were synthesized as water‐soluble single‐source precursors for low‐temperature hydrothermal decomposition toward covellite (CuS) nanoparticles. Their decomposition followed a reduction‐mediated pathway via Cu(I)‐dithiocarbamate intermediates, with the reduction step being rate‐limiting and readily tunable, enabling effective size‐shape control over the resulting CuS nanoparticles.

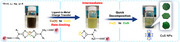

## 1 Introduction

Due to their tunable bandgaps, efficient photothermal conversion, low cost and good biocompatibility, nanoscale copper sulfides such as covellite (CuS) have been shown to have applications in a wide range of areas, including photothermal/photodynamic therapies, drug delivery, biomedical imaging, and catalysis [1, 2, 3, 4]. As their properties are size‐shape, morphology, and phase‐dependent, a large number of synthetic methods have been explored, with the so‐called single‐source precursor (SSP) approach being widely exploited as it allows a high degree of product tunability [2]. Here, molecular complexes with pre‐formed Cu‐S bonds are thermally decomposed, resulting in the loss of volatile organics and concomitant formation of thermodynamically stable nanomaterials. While many SSPs have been utilized for the synthesis of nanoscale copper sulfides [2, 5, 6, 7, 8, 9, 10, 11, 12], dithiocarbamate (DTC = S_2_CNR_2_) complexes have been particularly widely explored, a consequence of accessibility [6, 7]. Thus, DTCs are easily prepared from CS_2_ and secondary and primary amines [13], allowing simple substrate tunability, aqueous reactions with Cu(II) salts afford air and moisture‐stable bis‐DTC complexes, [Cu(κ^2^‐S_2_CNR_2_)_2_], in high yields, while other copper‐DTC complexes are also readily accessible in oxidation states of +1 to +3 [6].

**SCHEME 1:**
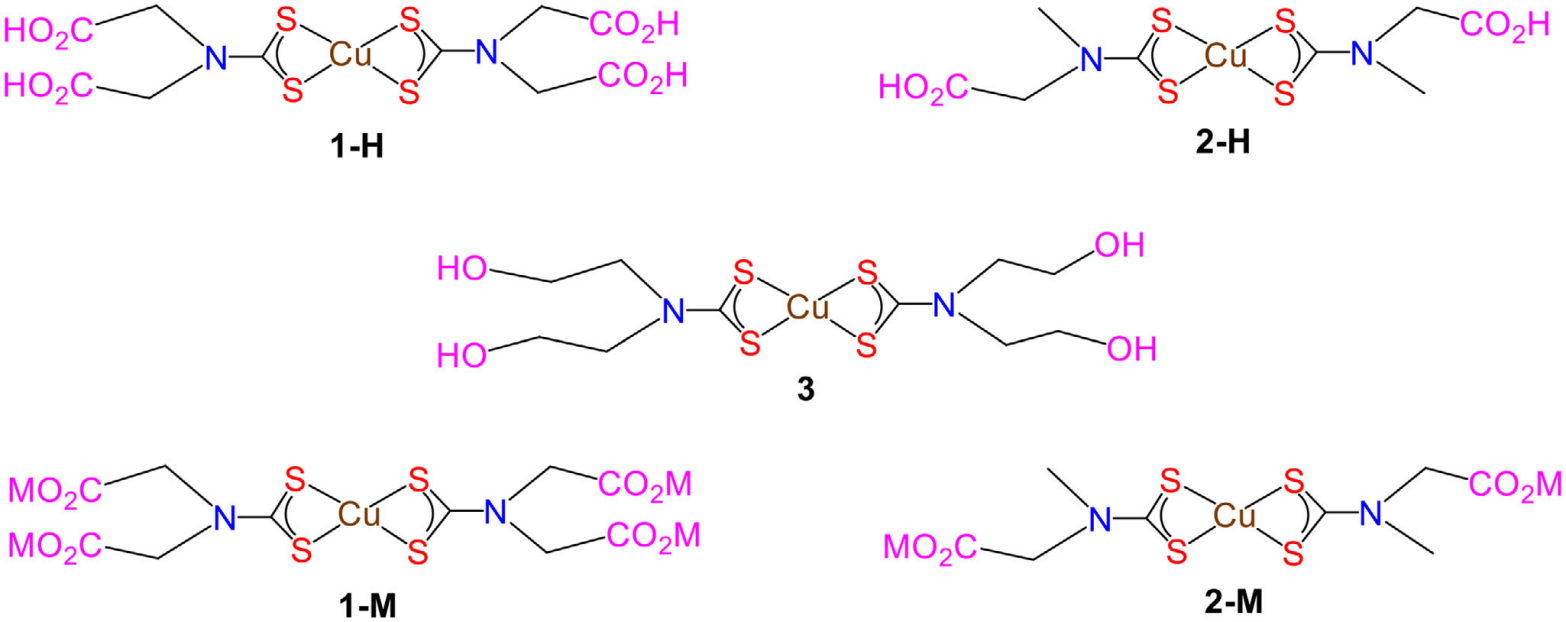
Cu(II) bis(DTC) SSPs used in this study (**M** = **Li**, **Na**, **K**).

Bis‐DTC complexes with alkyl groups have good solubility in a range of common organic solvents [6, 13, 14, 15], for example, those with long chain alkyl groups tend to be highly soluble in non‐coordinating solvents such as hexanes and CO_2_ [16] and also have high vapor pressures [17], making them suitable SSPs for both chemical vapor deposition (CVD) and aerosol‐assisted chemical vapor deposition (AA‐CVD) [7]. In our research, we aim to prepare nanoscale metal‐sulfides, including covellite (CuS), for applications in biomedicine and drug delivery. Thus, the ideal reaction medium, both to prepare and decompose the SSP, is water. In the literature, complexes containing the diethanolamine‐derived DTC such as [Cu{κ^2^‐S_2_CN(CH_2_CH_2_OH)_2_}_2_] (**3**) (Scheme 1) [18, 19, 20] are touted as being water‐soluble; however, while showing some water‐solubility, solubility of **3** is relatively low (see later). Other DTC ligands have been reported that confer water‐solubility on their complexes [21], but most are quite elaborate and not suitable for SSP studies. Many contain carboxylate groups, as do simple α‐amino acid‐derived DTCs [22], which affords water‐solubility of their complexes [23, 24, 25, 26, 27, 28, 29, 30, 31]. In this context, we recently communicated the synthesis and decomposition (in water) of the iminodiacetic acid (Imd) derived complex [Cu{κ^2^‐S_2_CN(CH_2_CO_2_H)_2_}_2_] (**1‐H**) (Scheme 1) [23]. Importantly, not only were we able to prepare and decompose **1‐H** in a single step, but decomposition occurred at ca. 90°C, producing CuS aggregates (ca. 100 ± 40 nm) composed of small individual nanocrystals (NCs) (ca. 8 ± 1 nm) [23]. Further, the Imd‐DTC ligands were found to act as capping agents, improving homogeneity and colloidal stability. Thus, unlike common dialkyl‐DTC complexes, which decompose in organic solvents at high temperatures (ca. > 200°C) [6], amino acid‐derived Cu‐DTC complexes offer a facile and efficient way of preparing monodisperse CuS NPs, rendering them candidates for conducting tunable synthesis in water.

In this contribution, we explore the use of iminodiacetic acid (Imd‐DTC) and sarcosine (Sar‐DTC) dithiocarbamate complexes: [Cu{κ^2^‐S_2_CN(CH_2_CO_2_H)_2_}_2_] (**1‐H**) and [Cu{κ^2^‐S_2_CNMe(CH_2_CO_2_H)}_2_] (**2‐H**), and their metalated derivatives **1‐M** and **2‐M** (Scheme 1) as SSPs toward covellite nanoparticles (NPs) and show that by simply varying pH, their size‐shape can be readily tuned. We also briefly study the diethanolamine‐DTC complex [Cu{κ^2^‐S_2_CN(CH_2_CH_2_OH)_2_}_2_] (**3**) as a “water‐soluble” standard. While the initial aim of our work was to establish standardized methods to reliably generate CuS NPs of varying size‐shapes in water, we have also established that the initial molecular decomposition process for Cu(II) complexes occurs via an intramolecular LMCT (Ligand‐to‐Metal Charge Transfer) process, resulting in generation of the analogous Cu(I)‐DTC complexes, which are the true SSPs. Further, we have established that formation of these intermediates is rate‐limiting in generating the molecular building blocks for CuS NPs generation and is related directly to the reduction potential of the Cu(II) SSP, which can be regulated via cation exchange in metalated derivatives. Thus, the generation of CuS NPs from Cu(II)‐DTC SSPs is reduction‐controlled. We have also isolated the Cu(I) intermediates as phosphine adducts and independently used them as SSPs, allowing the rapid generation of small (ca. 10 nm) CuS NPs suitable for biological applications. Monitoring these decomposition processes by UV–vis spectroscopy allows us to follow both the loss of SSP molecules and the formation of CuS NPs via their absorption(s) in the NIR. This work established a readily tunable synthesis of CuS NPs from water‐soluble SSPs and presented mechanistic insights into the underlying molecules‐to‐materials transformation, thereby offering a novel synthesis paradigm for metal‐chalcogenides.

## 2 Results and Discussion

### 2.1 Synthesis and Characterization of Water‐Soluble Cu(II)‐DTC Complexes

Cu(II)‐DTC complexes (Scheme 1) were prepared from DTC ligands Imd‐Na‐DTC [32] and Sar‐Na‐DTC [24] (Sections S1.2 and S1.5). Addition of CuSO_4_·5H_2_O (2:1 ratio) in water gives dark brown solutions, which, upon acidification with 3 m HCl, led to the precipitation of dark brown solids of [Cu{κ^2^‐S_2_CN(CH_2_CO_2_H)_2_}_2_] (**1‐H**) [25] and [Cu{κ^2^‐S_2_CNMe(CH_2_CO_2_H)}_2_] (2‐H) [33] (Sections S1.4 and S1.7). Similar reactions in water‐MeOH mixtures resulted in the immediate precipitation of [Cu{κ^2^‐S_2_CN(CH_2_CO_2_Na)_2_}_2_] (1‐Na) [25] and [Cu{κ^2^‐S_2_CNMe(CH_2_CO_2_Na)}_2_] (2‐Na) [34]. For [Cu{κ^2^‐S_2_CN(CH_2_CH_2_OH)_2_}_2_] (3), the DTC was generated in situ and the addition of CuSO_4_·5H_2_O resulted in the desired product in high yield [18]. Lithium and potassium salts of 1‐Li, 1‐K, 2‐Li and 2‐K were generated in solution upon adding LiOH or KOH to aqueous solutions of **1‐H** and **2‐H**, respectively.

Characterization was in accord with previous reports. Magnetic susceptibility measurements confirmed the presence of a Cu(II) center (Table S2), while IR spectra showed expected ν(C─S) and ν(C═N) vibrations of the DTC ligands, together with strong absorptions associated with the carboxylate‐carboxylic acid groups (Figure S1, Table S1). Metalation of the carboxylic acid groups shifts the ν(CO_2_) bands to lower wavenumbers by ca. 200 cm^−1^ as compared to their protonated counterparts, something previously noted by Liebing and co‐workers [25]. Elemental analyses support the formulations but show (as expected) binding of water to both the DTC salts of **1‐Na** and **2‐Na** [25]. ESI(+) mass spectra of **1‐H** (*m/z* = 478.88) and **2‐H** (*m/z* = 390.72) show the expected molecular ions for a Cu(DTC)_2_ stoichiometry (Figures S11 and S13). ESI(+) mass spectra of **1‐Na** and **2‐Na** (Figures S10 and S12) shows only peaks associated with degradation [25].

### 2.2 Water‐Solubility Studies

Before using these complexes as SSPs in water, we assessed their water‐solubility. While the diethanolamine‐derived DTC ligand is often considered to provide water‐solubility to its complexes, the water‐solubility of 3 at pH 7 is only 28.3 µg/mL (Figure S3a) and generally does not vary significantly below pH 11. Above this, there is a significant increase to ca. 496 µg/mL at pH 13 (Figure S3b). Dissolution of **1‐H** in water (3.5 mm) affords a solution of pH 2.9. It shows good water‐solubility over a wide range of pHs, being ca. 2300 µg/mL at pH 7. Like 3, the Sar‐DTC complex 2‐H is only slightly soluble in water at pH 7 (ca. 27 µg/mL), but upon raising the pH, its solubility significantly increases, being ca. 800 µg/mL at pH 9. We also briefly investigated solubilities in MeOH: **1‐Na** is completely insoluble, while **2‐Na** is only very slightly soluble. As noted earlier, **1‐H** and **2‐H** can easily be obtained by protonating their respective sodium salts, **1‐Na** and **2‐Na** with 3 m HCl. Consequently, Cu(II) bis(DTC) SSPs derived from α‐amino acids, such as Imd and Sar, have good potential as water‐soluble SSPs for the tunable aqueous synthesis of CuS NPs. Further, of the water‐soluble SSPs we have chosen (Scheme 1) there is a clear difference in their water‐solubilities: 1Na > 2Na > 1H > 2H >> 3H thus allowing us to potentially utilize this in tuning the generated CuS NPs.

### 2.3 Intramolecular Electron‐Transfer and Formation of Cu(I)‐DTC Complexes

**FIGURE 1:**
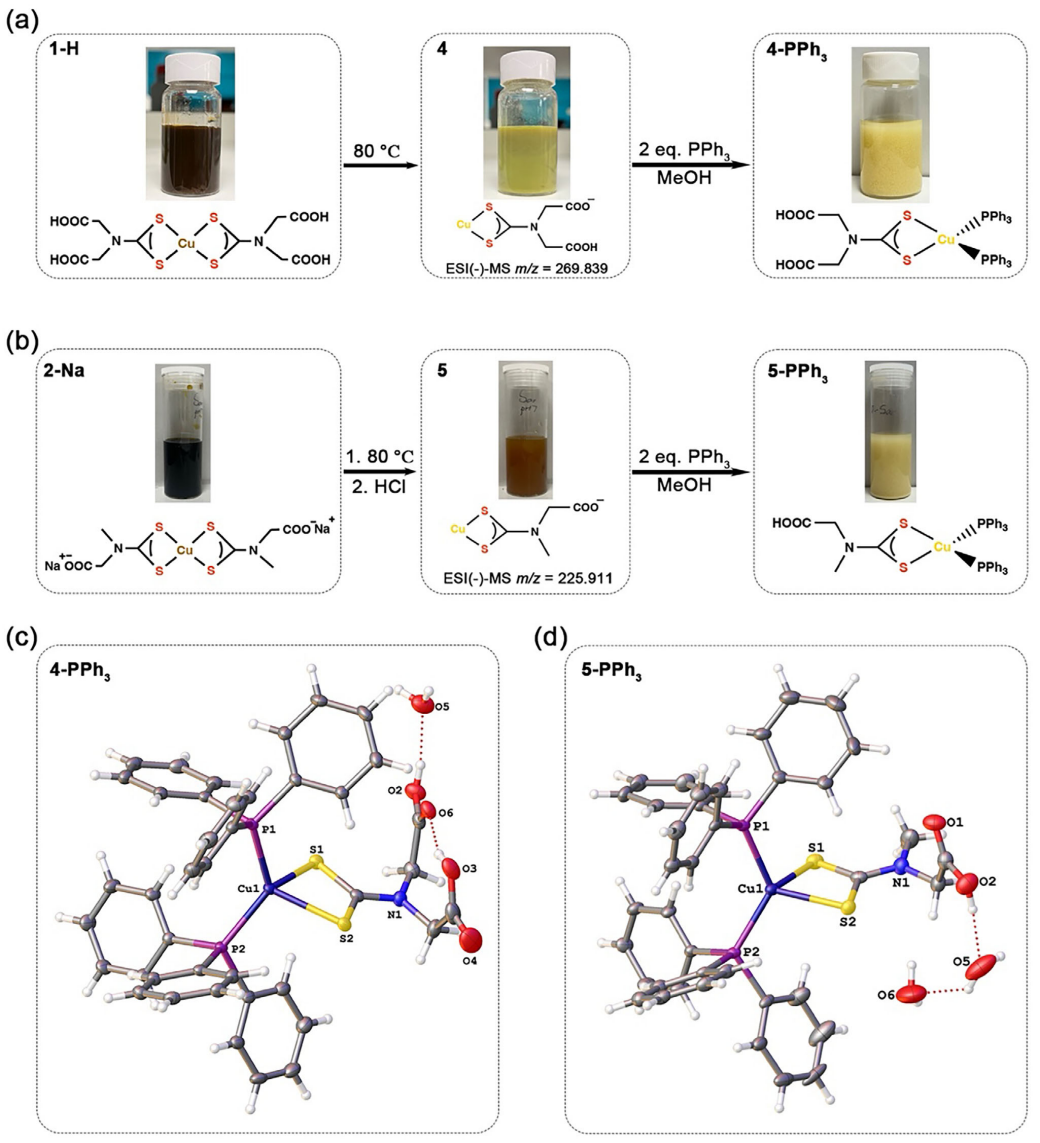
Images showing conversion of (a) **1‐H** and (b) **2‐Na** into **4‐PPh_3_** and **5‐PPh_3_**, respectively, upon heating in water at 80°C and reacting with PPh_3_ in MeOH; Molecular structure of (c) [Cu(PPh_3_)_2_{κ^2^‐S_2_CNCH_2_(CO_2_H)_2_}].3H_2_O (**4‐PPh_3_**).H_2_O and (d) [Cu{κ^2^‐S_2_CNMe(CH_2_CO_2_H)}(PPh_3_)_2_].2H_2_O (**5‐PPh_3_**).2H_2_O in the solid state.

The Cu(II)‐Cu(I) redox couple normally occurs at low potentials and is highly tunable, via judicious choice of supporting ligands [35]. Indeed, nature takes advantage of this, with copper‐containing species such as blue copper proteins serving as electron‐transfer mediators [36]. While redox potentials of [Cu(κ^2^‐S_2_CNR_2_)_2_] are tunable [37], they display long‐term stability in organic solvents since both reduction and oxidation events are inaccessible under these conditions. Thus, unexpectedly, we noted that upon standing at room temperature, aqueous solutions of **1‐Na** and **1‐H** (very) slowly turned from brown to yellow (over ca. 10–20 days), with the gradual deposition of a yellow precipitate. To expedite this transformation, a brown suspension of **1‐H** (ca. 20 mg/mL) was heated in water at 80°C for 2 h, resulting in conversion to a yellow suspension that was isolated by centrifugation to afford [Cu{κ^2^‐S_2_CN(CH_2_CO_2_H)_2_}]_n_ (4) in moderate yields (Figure 1a). As **2‐H** has poor solubility in water, we instead heated an aqueous solution of **2‐Na** at 80°C for 4 h, to give a light brown suspension, from which [Cu{κ^2^‐S_2_CNMe(CH_2_CO_2_H)}]_n_ (5) was precipitated after acidification (Figure 1b). Unfortunately, neither could be isolated pure via this method, with PXRD patterns showing small amounts of CuS (Figure S30). This suggests (as later confirmed) that Cu(I) complexes are intermediates in the decomposition of Cu(II)‐DTC into CuS. We note that Marcias and co‐workers have previously reported that amino acid‐derived Cu(II) DTC complexes undergo reduction to analogous Cu(I) species [38, 39].

Understanding that these new species were Cu(I) complexes, we screened reducing agents and found that dithiothreitol (DTT) was powerful enough to reduce the Cu(II) center. Thus, addition of an excess of DTT to an acidic aqueous suspension of **1‐H** resulted over ca. 1 h in the formation of a bright yellow precipitate, which, when collected, washed and dried, afforded **4** in ca. 70% yield. Complex **5** was formed in a similar way but required a higher concentration of DTT (Section S1.11). Importantly, warming aqueous solutions of either **4** or **5** to aid dissolution resulted in the yellow solution turning green rapidly and depositing a black solid shown by PXRD to be CuS (covellite) (Figure S41), thus confirming the intermediary of **4–5** in the thermal decomposition of **1–2** and confirms that the rate‐limiting step in the transformation of the latter to CuS is the reduction of Cu(II) to Cu(I). This is further validated and developed in later studies.

Complexes **4–5** isolated from the heating method were characterized by a suite of analytical methods. Magnetic susceptibility measurements showed that both are diamagnetic, confirming their Cu(I) nature (Table S2). IR spectra (Figure S1) shows the presence of ν(C─S), ν(C═N) and ν(CO_2_) absorptions, distinct from but similar to those observed for **1‐H** and **2‐H**. Interestingly, the ESI(‐)‐MS of **4** in MeCN shows an intense peak at *m/z* = 269.839, attributed to the anion generated from proton loss (Figure S19), while **5** shows a related intense peak at *m/z* = 225.911 (Figure S20). As compared to the Cu(II) complexes, both **4** and **5** have poor solubility in water (<0.1 mg/mL), which suggests that rather than adopting a tetranuclear cluster form [40, 41, 42], they are likely coordination polymers (or oligomers). Related Cu(I) xanthate complexes are coordination polymers, the precise linking arrangement being system‐dependent [43, 44].

The relatively facile reduction of the Cu(II) center in 1 and 2 in water may be a result of the electron‐withdrawing nature of the carboxylate groups. We recently reported a similar (very fast) LMCT process in [Cu(κ^2^‐S_2_CNHR)_2_], which converts rapidly and cleanly into yellow Cu(I) coordination polymers [Cu(κ^2^‐S_2_CNHR)]_n_ [45]. The mode of conversion of 1–2 to 4–5, respectively, is unknown. For related Cu(II) xanthate complexes, kinetic studies have revealed an intermolecular electron‐transfer, with the rate varying as a function of complex concentration [46]. In contrast, the loss rate of **1‐H** is independent of concentration (at 0.26, 1.04 and 2.08 mm), suggesting that conversion to 4 occurs via an intramolecular electron‐transfer (Figure S14a). Unlike the reported photoredox behavior of Cu(II)‐diethyldithiocarbamate mixed‐ligand complexes [47, 48], **1‐H** still exhibited significant reduction even when wrapped with foil (Figure S14b), indicating the reduction is photon‐independent charge transfer. The secondary product of this process is expected to be the corresponding thiuram disulfide (TDS). We attempted to confirm this by isolating and identifying the TDS, NMR spectra following workup of the aqueous phase showed only the free amines in both instances (Figures S15–S18). Tetra‐alkyl TDSs are normally easily prepared upon oxidation of the corresponding DTC salts with a variety of mild oxidizing agents [49] such as K_3_[Fe(CN)_6_] [50] or I_2_ [51] in water, although reactions may be more complicated in some cases [52] and over‐oxidation needs to be avoided [53]. TDSs are normally insoluble in water, but have good solubility in organic solvents, and thus simple phase extraction affords the pure compounds. We tried to prepare and isolate the TDS of Imd‐DTC but have had little success with either I_2_ or K_3_[Fe(CN)_6_]. Both gave precipitates, but analysis showed no evidence of the TDS.

### 2.4 Synthesis and Molecular Structures of [Cu(κ^2^‐DTC)(PPh_3_)_2_]

The low solubility of 4 and 5 in common organic solvents is akin to that noted for related Cu(I) coordination polymers, [Cu(κ^2^‐S_2_CNHR)]_n_ [45], hampering their precise characterization. A strategy we used in working with the latter was to prepare soluble bis(phosphine) adducts, [Cu(κ^2^‐S_2_CNHR)(PPh_3_)_2_], which we then characterized by NMR spectroscopy and X‐ray diffraction [45]. Adding ca. 2 equivalents of PPh_3_ to suspensions of either 4 or 5 in MeOH (Figure 1a,b) results in their slow conversion from dark to pale yellow solids, the latter being indicative of the formation of phosphine adducts. After work‐up (Section S1.12, Figure S21), pale yellow solids of [Cu{κ^2^‐S_2_CN(CH_2_CO_2_H)_2_}(PPh_3_)_2_] (4‐PPh_3_) and [Cu{κ^2^‐S_2_CNMe(CH_2_CO_2_H)}(PPh_3_)_2_] (5‐PPh_3_) were obtained in pure form. Both are slightly soluble in MeOH and have good solubility in CH_2_Cl_2_, allowing characterization by NMR spectroscopy. ^1^H NMR spectra show a 1:2 DTC:PPh_3_ ratio, consistent with formation of bis(phosphine) adducts, and ^31^P{^1^H} NMR spectra show a singlet resonance ca. ‐2 ppm (Figures S22–S27). Most importantly, in the ^13^C{^1^H} NMR spectra, the backbone DTC resonance is clearly observed at ca. 210–213 ppm (Figures S23 and S26). Slow evaporation of CHCl_2_/MeOH solutions led to the formation of crystals suitable for single crystal X‐ray diffraction (SCXRD) studies, the results of which are shown in Figure 1c,d.

The (highly) distorted tetrahedral coordination geometry around the Cu(I) center [S(1)‐Cu(1)‐S(2) 74.944(17)° in 4‐PPh_3_, 74.98(4) & 74.83(5)° in 5‐PPh_3_; P(1)‐Cu(1)‐P(2) 125.12(2)° in 4‐PPh_3_, 125.46(5) & 123.85(5)° in 5‐PPh_3_] is in accord with related [Cu(κ^2^‐DTC)(PPh_3_)_2_] structures [45]. In both complexes, the two carboxylate groups are protonated. In 4‐PPh_3_ (Figure 1c), they are linked by a hydrogen bond and there are also two co‐crystallized water molecules. The first (full occupancy) is hydrogen‐bonded to the second carboxylate group, while the second (not shown in Figure 1c) has only partial occupancy (0.25) and lies relatively close to O(4) [O(4)···O(1) 2.632(3) Å] but is not involved in hydrogen‐bonding. In 5‐PPh_3_ (Figure 1d), there are two symmetry‐independent molecules in the asymmetric unit (only one shown) that have slightly different hydrogen‐bonded interactions with co‐crystallized water, forming a chain with the carboxylate group in both.

### 2.5 Monitoring Cu(II)‐Cu(I) LMCT

Since the reduction of the Cu(II) center in 1–2 is the first step in the conversion to CuS nanomaterials, we next moved to consider how its rate varied as a function of pH. Brown Cu(II) bis‐DTC complexes have a characteristic strong absorption in the visible region at ca. 430 nm (ε = 13000 dm^3^ mol^−1^ cm^−1^ for [Cu(S_2_CNEt_2_)_2_] in CCl_4_) associated with a solvent‐independent equatorial ligand‐metal charge transfer [54]. In contrast, d^10^ Cu(I) complexes do not show strong absorption(s) in the visible region, while generated CuS nanomaterials are associated with a broad absorption(s) in the near IR (NIR) region associated with localized surface plasmon resonance (LSPR) which is a result of the plasmon resonance of free holes in Cu_2−x_S NPs [55]. The LSPR is a function of both NP size and shape, with larger particles tending to absorb at longer wavelengths [56, 57, 58, 59]. Thus, UV–vis spectroscopy is potentially a simple way of understanding the transformation of 1–2 into CuS NPs, with the relative difference between the loss of Cu(II) and growth of CuS, providing further insight into the stability of intermediate Cu(I) species.

**FIGURE 2:**
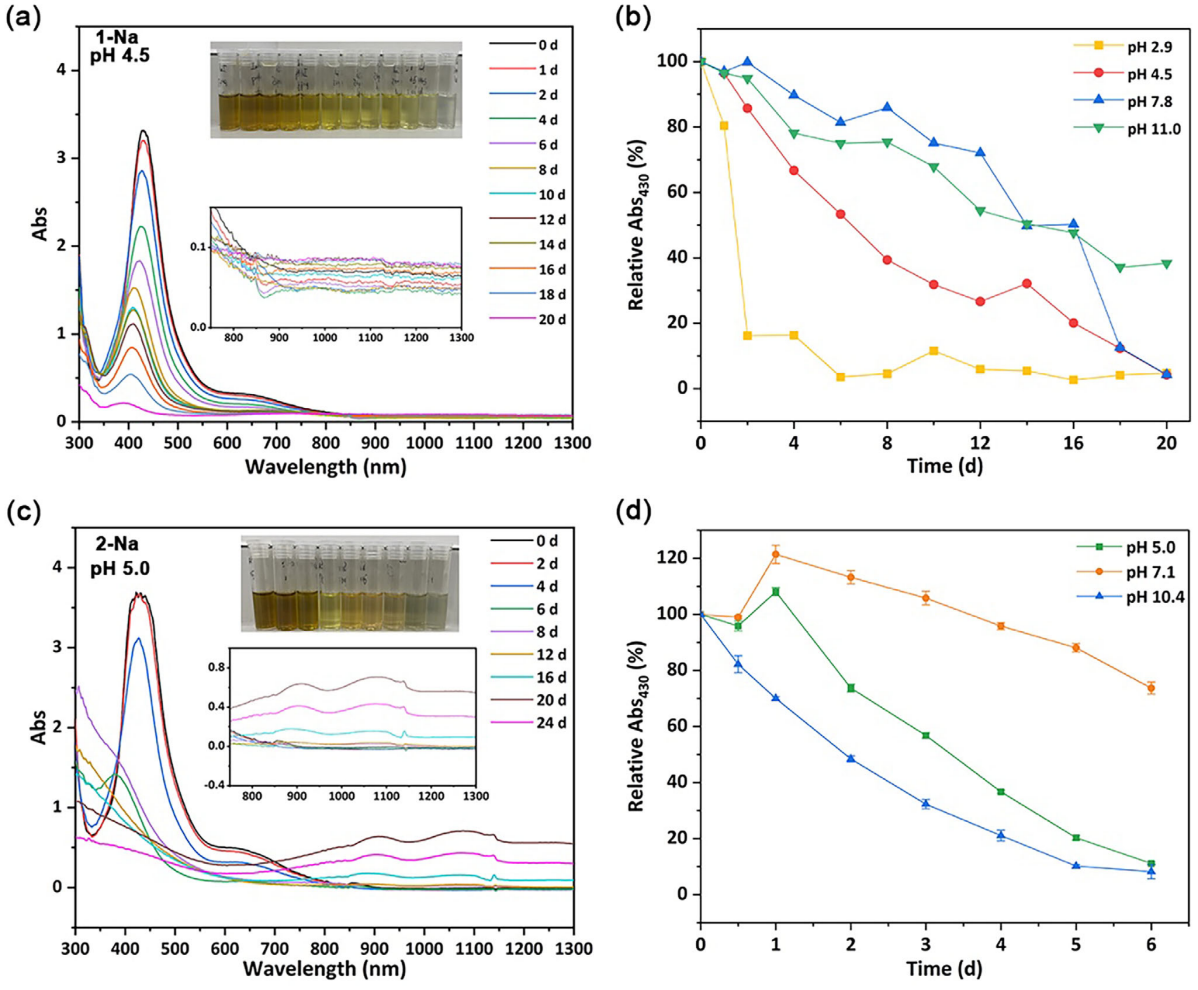
(a) UV–vis‐NIR spectra of **1‐Na** (pH 4.5) showing absorbance changes *vs* time and (inserted images) associated color changes, (b) plot showing the rate of decrease of Abs_430_ as a function of time (days) at various pH; (c) UV–vis‐NIR spectra of **2‐Na** (pH 5.0) showing absorbance changes *vs* time and (inserted images) associated color changes, (d) plot showing the rate of decrease of Abs_430_ as a function of time (days) at various pHs.

Accordingly, we studied the room temperature decomposition of various forms of 1 and 2 at a range of pH values (Section S1.13). In all these studies, 10 mg of **1‐H**/Na or 2‐Na was dissolved in 40 mL of deionized water, and the pH was adjusted via the addition of 0.1 m NaOH or 0.1 m HCl. Solutions were then sealed and stored at ambient temperature, and UV–vis‐NIR spectra were recorded over ca. 20 days. A representative example (**1‐Na** at pH 4.5) is given in Figure 2a, which shows a loss of intensity of the 430 nm absorption over time, and slow growth of a broad absorption in the NIR associated with the formation of CuS (insert) and changes in the color of the solution (top). Figure 2b gives a plot of the (normalized) relative intensity of the absorption maximum at 430 nm (Abs_430_) *vs* time at different pHs. The rate of Cu(II) reduction is highly pH dependent, significantly faster at lower pH. Thus, complete loss of **1‐H** is seen at pH 2.9 after ca. 2 days, while at pH 11, there is still a significant amount remaining after 20 days. As noted above, it is harder to comment directly on the rate of CuS formation as the NIR features are so broad due to the size dependence, but a general observation is that the growth of CuS is also faster at low pH (Figure S29; Figure 2). The more neutral the pH, the slower the reduction of the Cu(II) complex. Most solutions ended up as green suspensions of CuS, as confirmed by the slow increase in the intensity of NIR absorbance(s). For **1‐H** (pH 2.9), the NIR absorbance increases continuously until day 10, after which it begins to decrease, possibly a result of the dissolution of tiny CuS NPs in the strongly acidic environment. For **1‐Na** (pH 7.8), the NIR absorbance did not rise, suggesting that either the Cu(I) complex is stable under these conditions or NPs are small (<3 nm dia.). Importantly, the decrease in **1‐H**/Na and increase in NIR absorption of CuS are not simultaneous, suggesting that there is still an activation barrier for the conversion of 4 to CuS.

The pH dependency on the reduction of **2‐Na** follows a similar pattern (Figure 2d). As seen for **1‐Na**, the rate of loss of Cu(II) is pH‐dependent, being slowest at low pH 7.1 and being accelerated upon both raising and lowering the pH. In comparison to **1‐Na**, decomposition of **2‐Na** is faster, the latter being completely consumed after 6 days at both pH 5.0 and 10.4 (Figure S29). However, at pH 5 (Figure 2c) no significant NIR absorption(s) are seen until ca. 16 days, suggesting that there is a window (ca. 10 days) whereby the Cu(I) complex decomposes to form molecular building blocks that then aggregate to give small NCs, and these only slowly aggregate to give particles with a NIR absorption, which then slowly increases over the next 8 days. Importantly, there is no shift of the developing NIR absorption to higher wavenumbers, suggesting that during the NC growth, the overall range of NP diameters remains approximately the same.

Thus, we have confirmed here that the first step in the decomposition of Cu(II) SSPs **1** and **2** is their reduction to afford the corresponding Cu(I) complexes, as shown by the formation of bright yellow solutions for all samples, being indicative of Cu(I)‐DTCs [40, 41, 42]. A preliminary study suggests that this occurs via an intramolecular electron‐transfer, as it is not concentration‐dependent (Figure S14a). The rate of electron‐transfer is, however, strongly dependent on pH, being slowest at pH ca. 7 and accelerated especially at lower pHs, while the Sar complex **2‐Na** decomposes at a significantly faster rate than the Imd‐derivative **1‐Na**. To further understand these differences, we have probed their redox chemistry at various pHs, with the general premise that a lowering of the reduction potential would accelerate electron‐transfer.

### 2.6 Cyclic Voltammetry of [Cu(DTC)_2_]

**SCHEME 2:**
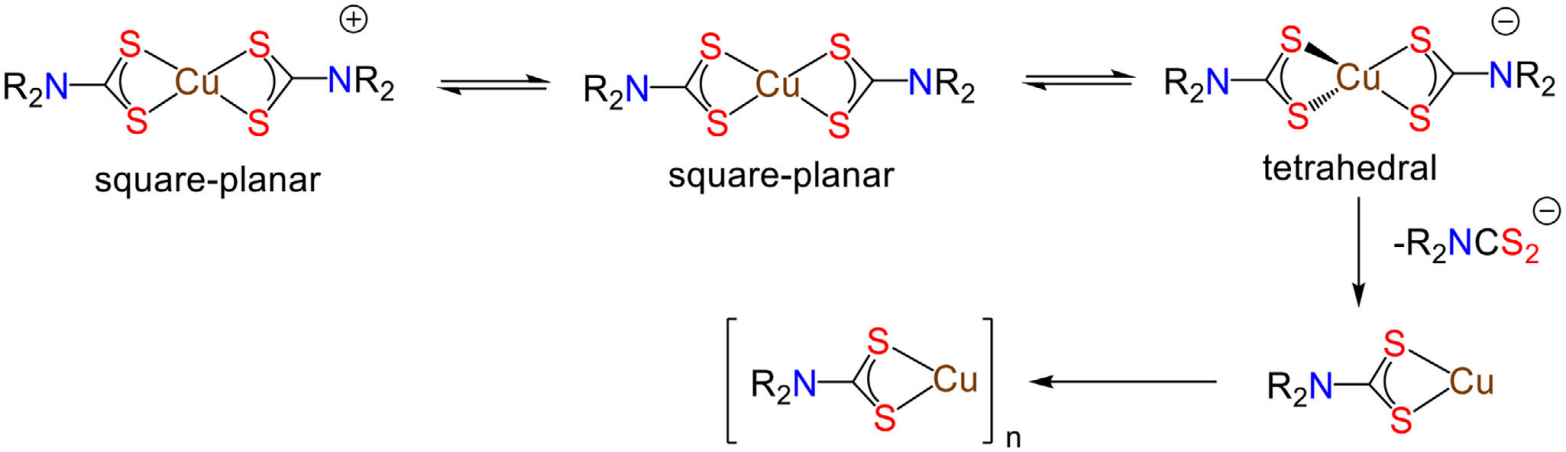
Summary of the redox chemistry of [Cu(κ^2^‐S_2_CNR_2_)_2_].

The redox chemistry of bis‐DTC complexes, [Cu(κ^2^‐S_2_CNR_2_)_2_], has been widely studied previously [6, 37] (Scheme 2). Briefly, they show a reversible one‐electron oxidation to afford green Cu(III) cations, [Cu(κ^2^‐S_2_CNR_2_)_2_]^+^, and a quasi‐reversible one‐electron reduction to afford Cu(I) anions, [Cu(κ^2^‐S_2_CNR_2_)_2_]^−^. While Cu(III) cations can be isolated and fully characterized [6, 60, 61], the labile (d^10^) nature of the Cu(I) center results in facile DTC loss to afford Cu(I) species that aggregate to (normally) give tetranuclear clusters [Cu(µ‐S_2_CNR_2_)]_4_ [40, 41, 42].

**FIGURE 3:**
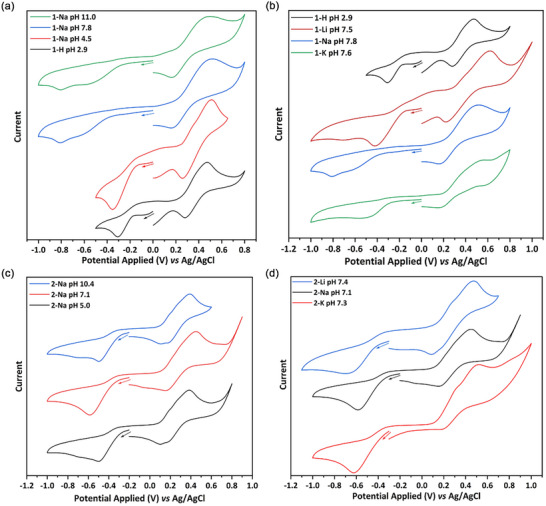
CVs for (a) **1‐H** and **1‐Na** at different pHs; (b) **1‐M** complexes with different cations; (c) **2‐Na** complexes at different pH; (d) **2‐M** complexes with different cations.

To gain further insight into Cu(II)‐to‐Cu(I) transformation, we carried out a series of cyclic voltammetry (CV) experiments in H_2_O at 25°C (Figure 3 and Table 1). The oxidation chemistry for **1‐Na** and **2‐Na** is (broadly) as expected. Each displays a (quasi‐reversible) one‐electron oxidation, the potential of which does not vary significantly as a function of pH: **1‐Na** (pH 7.8) *E*_1/2_ = 0.40 V (Δ*E*_p_ 194 mV), while for **2‐Na** (pH 7.1) *E*_1/2_ = 0.30 V (Δ*E*_p_ 295 mV). These oxidation potentials are slightly lower than found for analogous dialkyl‐derivatives (measured in acetone) [37], and more notably, the peak‐peak separations (ΔE_p_) are much greater than those for dialkyl‐derivatives, which are electrochemically reversible [37]. This difference may result from the coordination of water to the vacant sites of the cationic Cu(III) center. Changing the cation (M = Li, K) results in only small changes to the oxidation potential but does result in a significant increase in ΔE_p_ (for **1‐M** M = Li, 387 mV; M = K, 399 mV), also likely due to the degree of water binding.

**TABLE 1:** CV parameters (*vs* Ag/AgCl) for Cu(II)‐DTC complexes in water (scan rate = 100 mV s^−1^).

Complex	pH	*E*_1/2 ox_ (V)	Δ*E*_p_ (mV)	*E*_red_ (V)
**1‐Na**	11.0	0.41	226	−0.78 ± 0.02
**1‐Na**	7.8	0.4	194	−0.82 ± 0.01
**1‐Na**	4.5	0.37	224	−0.35 ± 0.02
**1‐H**	2.9	0.38	264	−0.34 ± 0.02
**1‐Li**	7.5	0.42	387	−0.41 ± 0.01
**1‐K**	7.6	0.33	399	−0.62 ± 0.04
**2‐Na**	10.4	0.25	288	−0.50 ± 0.01
**2‐Na**	7.1	0.3	295	−0.60 ± 0.01
**2‐Na**	5.0	0.25	289	−0.52 ± 0.02
**2‐Li**	7.4	0.29	451	−0.73 ± 0.03
**2‐K**	7.3	0.35	373	−0.62 ± 0.01

In contrast to the behavior noted for dialkyl [Cu(κ^2^‐S_2_CNR_2_)_2_] [37] or diaryl [Cu(κ^2^‐S_2_CNAr_2_)_2_] [14] derivatives (in acetone), which exhibit a quasi‐reversible one‐electron reduction, **1‐Na** and **2‐Na** show irreversible reduction chemistry at all pHs, not unexpected as water will significantly stabilize the [DTC]^−^ leaving group. The reduction potential of **1‐Na** is pH‐dependent. Thus, at pH 7.8, E_red_ of **1‐Na** is −0.82 V, but shifts to ca. −0.35 V at pH 4.5, and then does not change significantly at pH 2.9. Similarly, E_red_ at pH ca. 7 is lower for both **1‐Li** (E_red_ −0.41 V) and **1‐K** (E_red_ −0.62 V). For **2‐Na** at pH 7.1 (E_red_ is −0.60 V), reduction occurs at slightly lower potentials than for **1‐Na** and is less sensitive to changes in pH. There is some change in the reduction potential upon changing the cation, with both **2‐Li** (ca. −0.73 V) and **2‐K** (ca. −0.62 V) reducing at slightly higher potentials. Thus, considering that Cu(II)‐DTC reduction is the initial and rate‐determining step for decomposition to CuS, then modulating the reduction potential by changing cations provides a potential handle to tune the generated CuS NPs (see later).

### 2.7 Thermal Stability of Cu‐DTC SSPs in the Solid State

While our decomposition studies are in aqueous solutions, to better understand the relative thermal stability of the SSPs in this work, we have compared the thermal stability (in the solid state) of Cu(II) and Cu(I) DTC complexes (Figure S28). For the Cu(II) complexes, upon heating to 100°C, there is some mass loss (together with strong endothermic peaks) associated with the coordination of water, being greatest for the sodium salts. The most notable difference between protonated and metalated complexes is significantly decreased decomposition temperature of the protonated *vs* metalated complexes, with **1‐H** and **2‐H** starting to decompose at ca. 180°C via a process that has a sharp mass loss (ca. 33% for **1‐H** and ca. 45% for **2‐H**), both being close to the mass of a DTC ligand, and thus consistent with the formation of Cu(DTC) species. Following this, decomposition of **2‐H** is relatively rapid and is complete by ca. 250°C, while, in contrast, further mass loss from **1‐H** is slow and significant changes are seen even above 400°C. Thus, while the two complexes have a similar first decomposition step, the breakdown of the coordinated DTC ligands to afford sulfide is very different and likely proceeds via quite different pathways. By comparison, their respective sodium salts to **1‐Na** and **2‐Na** are far more stable than their protonated analogues [32], with significant mass loss only occurring at ca. 250 and 300°C, respectively (Figure S28a,b, Table S1). This is fully in accord with related observations made by Liebing and co‐workers [25].

As Cu(I) DTC complexes **4** and **5** are intermediates in the decomposition of **1, 2‐H**, we have also looked at their decomposition by TGA/DSC (Figure S28c,d). Both lose co‐crystallized water but are then stable up to ca. 170°C. Above this initial mass loss, the decomposition of **4** follows that of **1‐H** quite well, with a slow mass loss up to ca. 350°C, broadly consistent with the formation of **4** upon heating **1‐H** at 180°C. However, there are some more significant differences between **5** and **2‐H**, the former showing significant mass loss above 250°C that is not observed for **2‐H**. It is not easy to explain this difference, but we note that at room temperature, **5** is proposed to be oligomeric, while when generated via DTC upon heating **2‐H**, it may be in a quite different aggregated state.

### 2.8 Hydrothermal Decomposition of Water‐Soluble Cu(II)‐DTC SSPs

We next looked at using water‐soluble Cu(II)‐DTC complexes as SSPs toward CuS (covellite) NPs. We have focused primarily on the Imd system as these have the highest water‐solubility, but also present analogous Sar chemistry for comparison, together with a brief study of the aqueous decomposition of [Cu{κ^2^‐S_2_CN(CH_2_CH_2_OH)_2_}_2_] (**3**). We varied several parameters, including temperature, backbone substituents, pH and counter‐ions, finding that while some have only a small effect on the generated CuS NPs, others allow the size‐shape of the produced NPs to be easily tuned. Earlier, we identified the reduction step and initial generation of Cu(I) intermediate as a key step in the overall decomposition process. We have also studied the decomposition of isolated Cu(I) complexes **4** and **5** and have tried to correlate the rate of decomposition of Cu(II) SSPs, and consequent control over NC size, with the Cu(II)‐Cu(I) reduction potential.

#### 2.8.1 Effect of Temperature on CuS NPs Obtained From **1‐Na**

**FIGURE 4:**
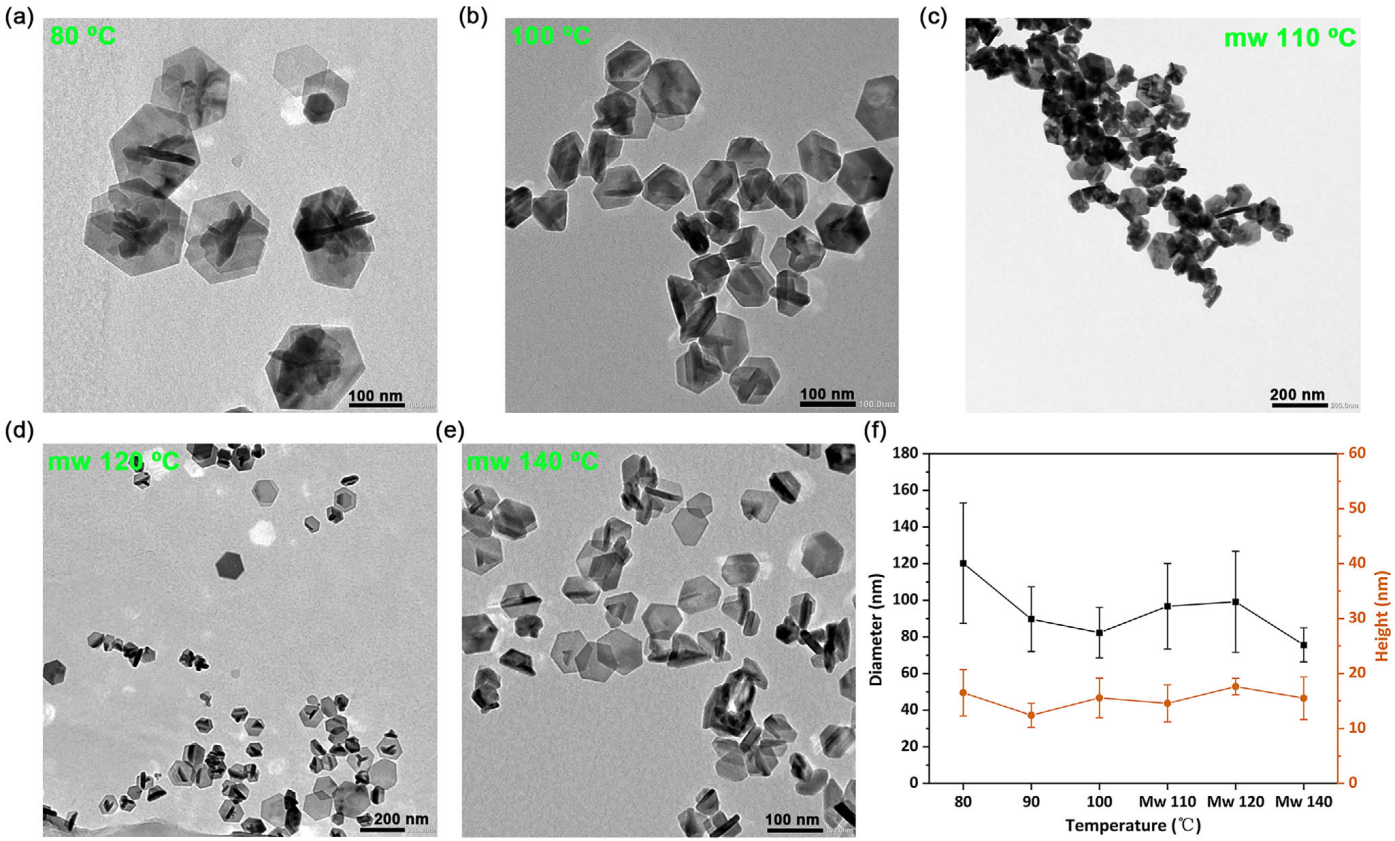
Decomposition studies of **1‐Na** (pH 7.8) at various temperatures. TEM images of NPs prepared at (a) 80°C; (b) 100°C; (c) 110°C; (d) 120°C; (e) 140°C. (f) Sizes of NPs obtained at different temperatures. Microwave‐assisted decompositions are abbreviated as “mw”.

**FIGURE 5:**
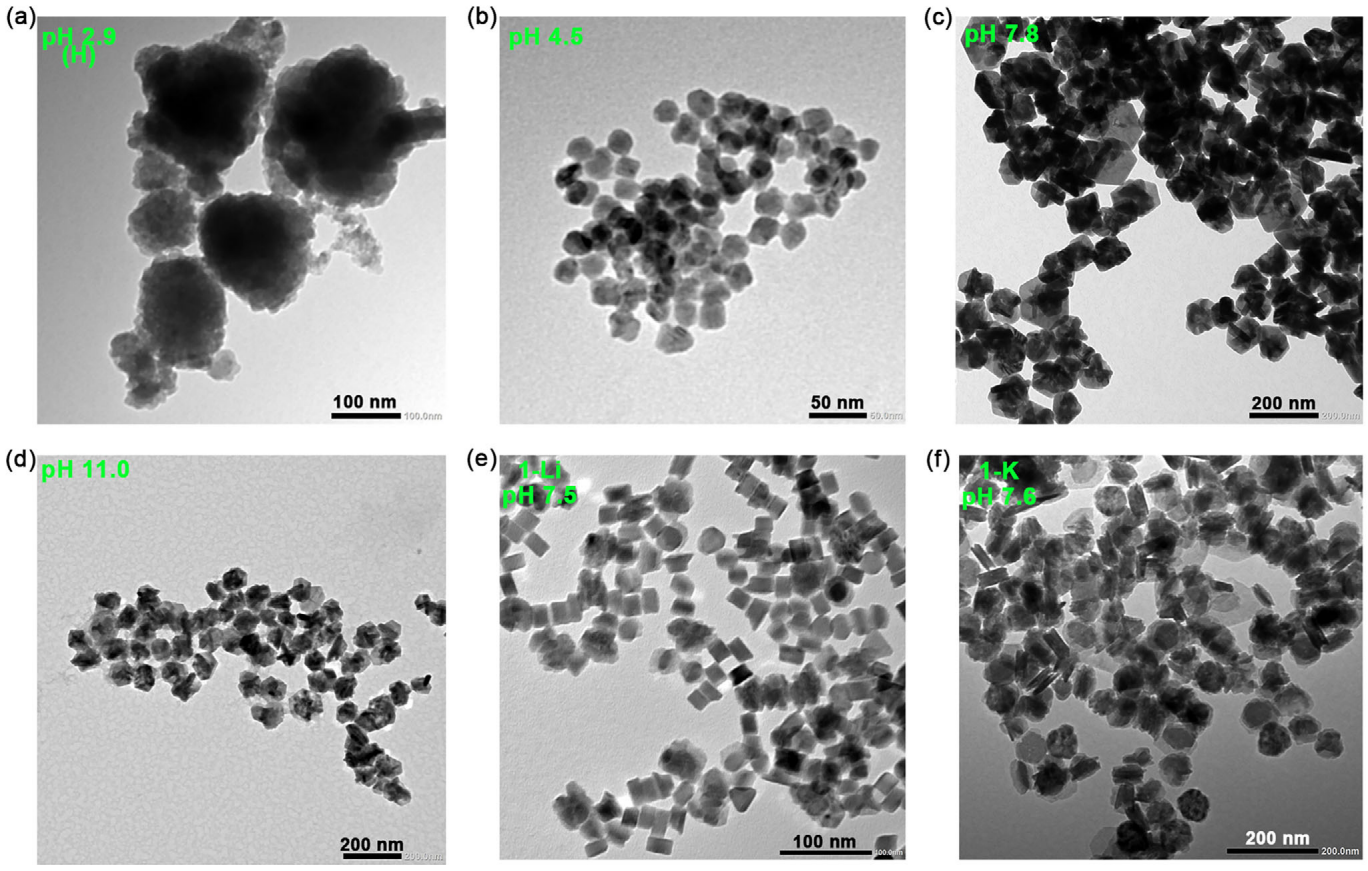
Decomposition studies of **1** at 90°C. TEM images of NPs from (a) **1‐H** (pH 2.9); (b) **1‐Na** (pH 4.5); (c) **1‐Na** (pH 7.8); (d) **1‐Na** (pH 11.0); (e) **1‐Li** (pH 7.5); (f) **1‐K** (pH 7.6).

Varying the decomposition temperature is widely used in SSP chemistry as a way of tuning both the size and morphology of the generated NPs; higher temperatures generally favor faster nucleation, resulting in numerous smaller NPs [62, 63]. To study the effects of temperature, we focused on **1‐Na** at pH 7.8 as the SSP. Five different temperatures were explored, with decompositions of 3.5 mm solutions at 80, 90 and 100°C using the hot‐injection method, heating for 4 h after injection (Section S1.15). Higher temperatures were achieved using a microwave reactor, but heating was only needed for 20 min to reach full conversion into green suspension (Section S1.15). During the microwave‐assisted decompositions, the initial brown solutions turned green, with the isolation of a dark green suspension of NPs after standard work‐up. Although complex solutions decomposed below 100°C, they remained brown (due to the high extinction constant of Cu(II)‐DTC complexes), but a significant amount of CuS NPs were isolated (after ultracentrifugation). In all cases, NPs obtained were identified as CuS (Covellite, JCPDS No. 06–0464) via PXRD (Figure S36) with NP size‐shape being determined by TEM (Figure 4a–e, NPs from **1‐Na** at 90°C are demonstrated in Figure 5c).

Most NPs formed were hexagonal plates. Figure 4f gives the average diameter (black) and thickness (orange) of the generated NPs. The thickness of the plates does not vary significantly (or controllably) with temperature, being on average ca. 15 nm. In contrast, average diameters do vary, although there is a wide distribution. Thus, via hot injection, increasing the temperature affords both smaller and more homogeneous NPs, of ca. 80 ± 30 nm at 100°C. At higher temperatures under microwave irradiation, NP diameter changes non‐linearly with temperature, although a similar aspect ratio (diameter/height) is maintained. NPs have a broader distribution of diameters at 110°C and 120°C, while at 140°C the average diameter decreased by ca. 25 nm with NPs becoming more uniform (Figure 5f; Figure S31). Thus, while there is some variation in NP diameters as a function of temperature, changes to their size and distribution are small.

#### 2.8.2 Effect of Changing pH and Cation for 1‐M

We next turned our attention to how changes in pH and the counter cation would affect the decomposition product of **1‐M**. All these experiments were carried out at 90°C for 4 h in a reflux system via hot injection. As detailed above, in all cases the initially clear dark brown solutions gave a green suspension that was shown to be CuS (covellite) as confirmed by PXRD (Figures S37 and S38). The size‐morphology of the nanomaterials generated was again probed by TEM (Figure 5). Both the size and morphology of the generated NPs vary significantly with pH. Thus, at pH 7.8, hexagonal plates of ca. 15 nm thickness and 90 nm diameter result. Upon lowering the pH, both the size and shape of the NPs are significantly altered, with particles getting smaller (21 nm at pH 4.5; 16 nm at pH 2.9) and becoming more spherical. Further, small NPs aggregate into large clusters, a consequence of the absence of a significant surfactant. This behavior, namely the formation of large aggregates of small NPs, is consistent with our earlier communication [23]. Interestingly, upon raising the pH to 11.0, while the size of the NPs decreases (from dia. 90 nm to dia. 68 nm), the morphology (hexagonal plates) and thickness (ca. 14 nm) remain unchanged. Thus, unlike temperature, variations in pH have a significant effect upon the size and morphology of the generated NPs.

We also considered whether the nature of the counter cation, simply altered by adjusting the pH (to ca. 7.5–7.8) of an aqueous solution of **1‐H** by addition of LiOH (**1‐Li**) or KOH (**1‐K**), would play a role in controlling the size‐shape of the generated NPs as it affects the reduction potential. While **1‐Na** decomposes at pH 7.8 to afford large hexagonal plates (ca. 90 nm × 15 nm), under similar conditions **1‐K** gave smaller plates but of significantly reduced thickness (61 nm × 5 nm), while with **1‐Li** the NPs were much smaller (ca. 26 nm) but of a similar thickness (ca. 14 nm) to those generated from **1‐Na**. These differences relate to the relative reduction potentials of the different salts at pH ca. 7.5 (Table 1). Thus, the lowest reduction potential (**1‐Li**: −0.41 V) generates Cu(I) building blocks rapidly, giving multiple nucleation sites resulting in small NPs, while the highest reduction potential (**1‐Na**: −0.82 V) generates reactive monomers only slowly and therefore yields large, well‐formed NPs. Thus, we term this approach a reduction‐controlled tunable synthesis of covellite (CuS) NPs.

#### 2.8.3 Decomposition of Sar Complexes—Effects of Changing pH and Cation

**FIGURE 6:**
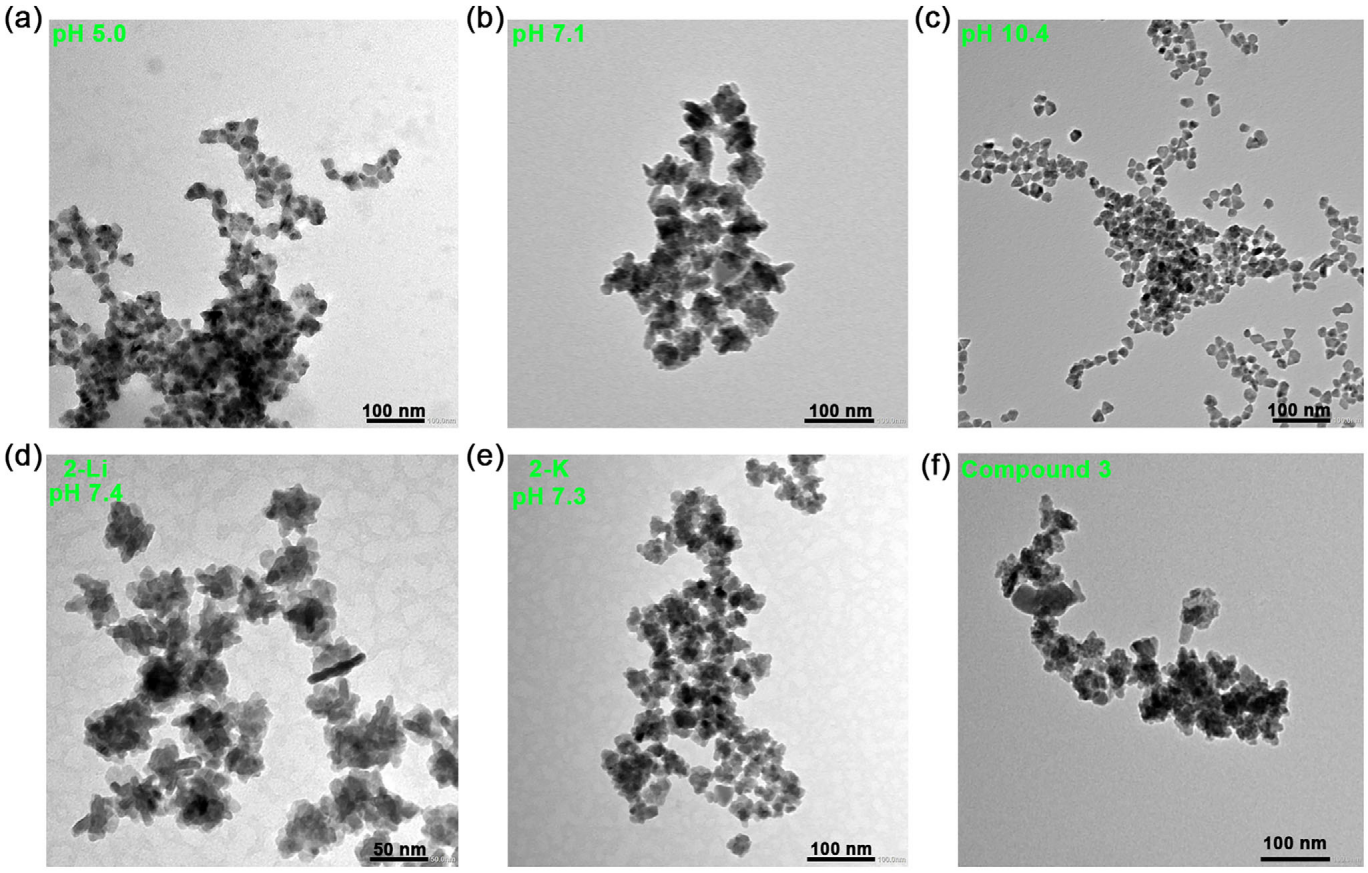
Decomposition studies of **2** at 90°C. TEM images of NPs from (a) **2‐Na** (pH 5.0); (b) **2‐Na** (pH 7.1); (c) **2‐Na** (pH 11.0); (d) **2‐Li** (pH 7.4); (e) **2‐K** (pH 7.3); (f) **3**.

We next turned our attention to the corresponding Sar SSPs, all decompositions being carried out at 90°C for 4 h, and again, under all conditions, CuS (covellite) was exclusively formed (Figures S39 and S40). In comparison to the respective Imd‐SSPs, under similar experimental conditions, significantly smaller NPs were produced (Figure 6). For example, while **1‐Na** decomposes at pH 7.8 to afford large hexagonal plates (ca. 90 nm × 15 nm), under similar conditions (pH 7.1), **2‐Na** gives much smaller (ca. 18 nm) irregular NPs. Further, and quite unlike the behavior found for **1‐Na**, lowering the pH (to 5.0) had very little effect on NP size. Although increasing the pH (to 10.4) barely changed the size, NP from this condition revealed regular shapes, such as triangular and hexagonal disks, which can be utilized for customizable synthesis. Similar insensitivity to changes in the cations was also noted, **2‐Li** decomposing at pH 7.4 to afford slightly smaller NPs (ca. 11 nm) than for **2‐Na**, while those obtained upon decomposition of **2‐K** at pH 7.3 were virtually identical to **2‐Na**. We have also decomposed [Cu{κ^2^‐S_2_CN(CH_2_CH_2_OH)_2_}_2_] (**3**) under analogous conditions (pH ca. 7, 3.5 mm, 90°C, 4 h) and find that small (ca. 10 nm) irregular NPs result. Thus, while **2** and **3** are potentially useful SSPs toward the synthesis of small CuS NPs, control of the size and shape of the generated NPs is less significant.

#### 2.8.4 Following Thermal Decomposition by UV–vis Spectroscopy

**FIGURE 7:**
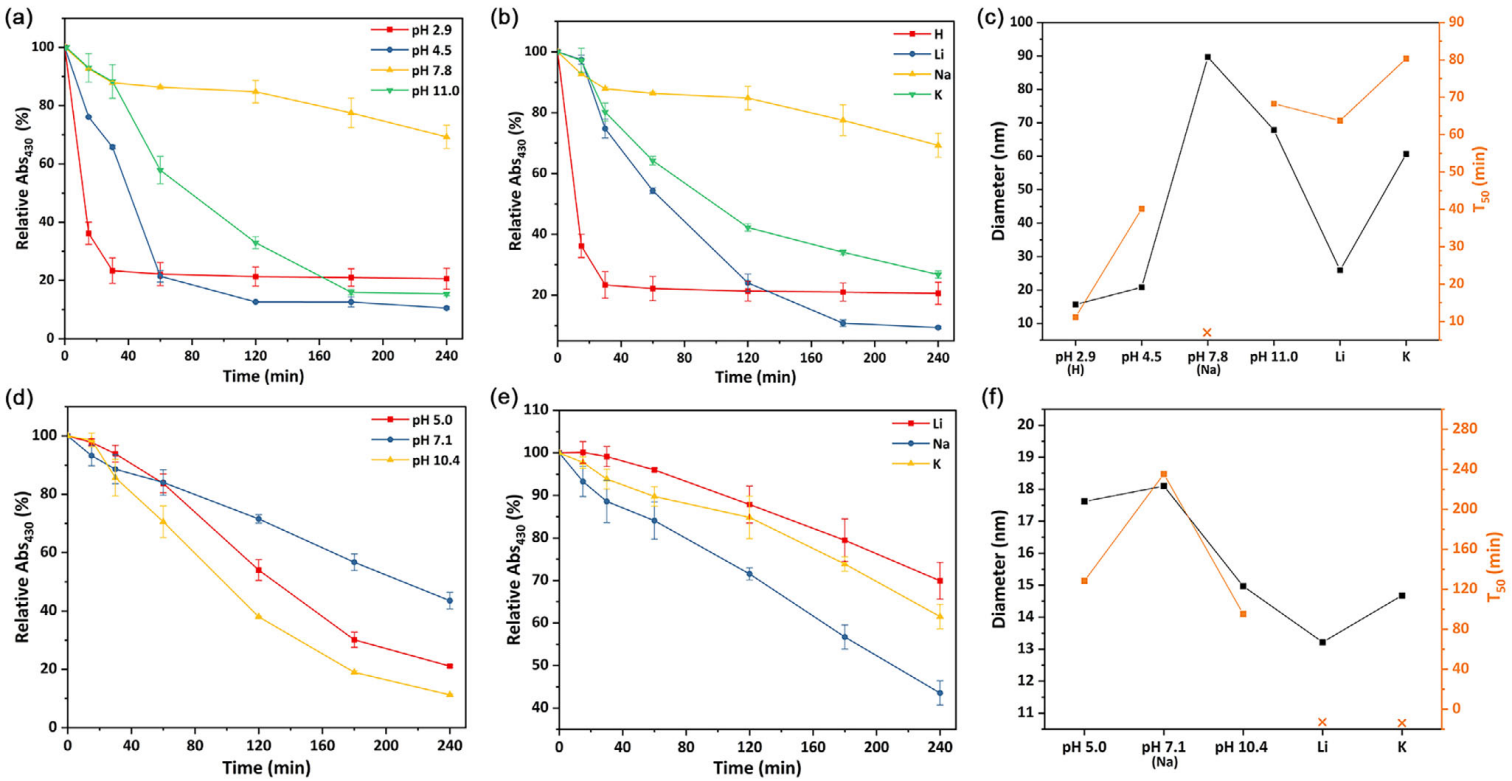
(a) Variation of relative Abs_430_ (%) when **1‐Na** decomposed at different pH (*n* = 3); (b) variation of relative Abs_430_ (%) when **1‐M** decomposed at pH ∼ 7.5 (*n* = 3) (Abs_430_ the absorbance at 430 nm); (c) correlation between the diameter of NPs and T_50_ from different decompositions. (d) Variation of relative Abs_430_ (%) when **2‐Na** decomposed at different pH (*n* = 3); (e) variation of relative Abs_430_ (%) when **2‐M** decomposed at pH ∼ 7 (*n* = 3) (Abs_430_ the absorbance at 430 nm); (f) correlation between the diameter of NPs and T_50_ from different decompositions. T_50_ – time for the Abs_430_ to drop to 50% calculated according to the fitting of Abs_430_‐Time graphs; Diameter—the diameter of discrete NPs; Yellow X means T_50_ > 240 min, which exceeds the curve's fitting range.

A key challenge when trying to design SSPs that can be tuned to prepare a range of bespoke nanomaterials is to better understand the nature and rate(s) of molecular processes that occur during the conversion of SSPs into nanomaterials [7, 64, 65, 66, 67, 68]. This is difficult as there is (generally) little cross‐over between the analytical techniques used to probe the molecular and nanoscale‐materials domains. One technique that does potentially apply to both is UV–vis spectroscopy. As discussed earlier, we have used this to probe the conversion of Cu(II) to Cu(I) DTC SSPs. We have also monitored decompositions of **1‐Na** and **2‐Na** (at various pH) and **1‐M** and **2‐M** (at pH ca. 7.5) as described in the preceding two sections, by UV–vis spectroscopy (Figure 7). To do this, aliquots were taken at various times and the absorbance at 430 nm (Abs_430_) measured. Each experiment was carried out three times and the results were averaged. During each decomposition, we also monitored the growth of a broad band(s) in the NIR (Figures S32–S35). The broad nature of these makes any detailed analysis difficult, but the final maximum absorbance wavelength in the NIR (A_max_) does relate to some extent to the average particle size and is listed in Table 2.

**TABLE 2:** CuS NP morphologies and sizes from different SSPs.

SSP		Shape	Size (d. nm)	WL of NIR A_max_ (nm)
1	pH 2.9	NC clusters	∼ 16	1050
	pH 4.5	Spheres	∼ 21	1070
	pH 7.8	Hexagonal plates	∼ 90 (10^h^ ^)^)	1100
	pH 11.0	Hexagonal plates	∼ 68 (14^h^ ^)^)	1090
	Li (pH 7.5)	Hexagonal columns	∼ 26 (14^h^ ^)^)	1250
	K (pH 7.6)	Hexagonal plates	∼ 61 (5^h^ ^)^)	960
2	pH 5.0	Irregular particles	∼ 18	1125
	pH 7.1	Irregular flakes	∼ 18	1050
	pH 10.4	Triangular/hexagonal disks	∼ 15	1016
	Li (pH 7.4)	Irregular disks	∼ 11 (7 ^h^)	1100
	K (pH 7.3)	Irregular spheres	∼ 15	1000
3		Irregular particles	∼ 10	—
4		Irregular spheres	∼11	940
5		Irregular disks	∼11 (6^h^ ^)^)	1140

^h^ means height.

As found for the earlier room temperature decomposition and reduction studies, the rate of decomposition of **1** is highly pH dependent, being accelerated upon both decreasing and increasing the pH (Figure 7a,c). Thus, at pH 2.9, loss of **1** occurs over ca. 15 mins, while at pH 7.8, only ca. 20% decomposition has occurred after 4 h. Thus, at pH 7.8, reactive monomer building blocks are only very slowly released into the solution, while in contrast, at pH 2.9, they are released very rapidly. The latter allows for burst nucleation and formation of many nucleation sites, thus leading to the formation of small, uniform CuS NPs. In contrast, at pH 7.8, the monomer is slowly and consistently generated, resulting in a small number of nucleation sites that slowly grow over time, thus affording large well‐formed NPs but with a relatively high dispersity. Similar, though less pronounced, changes to the decomposition rates of SSPs with different cations are also found (Figure 7b,c), again accounting for the different NP sizes, although within a much narrower range.

For the sarcosine complexes, a broadly similar but less pronounced pH trend is noted (Figure 7d), the rate of decomposition being increased (by approximately the same amount) upon raising or lowering the pH, but change(s) are smaller. Likewise, there is a less pronounced variation with respect to the cation, likely a result of being a dicationic *vs* a tetracationic system. Consequently, while changes are seen in the decomposition rates, the less significant differences are reflected in the smaller range of NP sizes generated (Table 2). Further, CuS NPs generated from **2** have a range of morphologies, from nanospheres and nanoflakes to triangular/hexagonal plates, and can be tailored by adjusting the reduction potential (Figure 6a–e). Both **2‐Li** and **2‐K** have similar reduction potentials and thus decompose into NPs of similar sizes (Figure 6d,e and Figure 7f).

Thus, the rate of Cu(II)‐Cu(I) electron‐transfer controls the size, shape and dispersity of the generated NPs, which, in turn, is directly related to our measured reduction potential(s) (see above). Hence, this system is predominantly reduction‐controlled, with reduction of the Cu(II) pre‐SSP to the actual Cu(I)‐SSP being rate‐limiting. These observations align with our CV and room temperature solution‐stability tests, pre‐SSPs with a lower reduction potential leading to faster decomposition and smaller NPs. Thus, modulating the reduction potential serves as an effective strategy to tune the decomposition of Cu(II)‐bis(DTCs). Further, CuS NPs with different morphologies generate different absorption patterns in the NIR region (Figures S32–S35), with the wavelength of the maximum absorbance differing (Table 2). This highlights the shape‐dependent nature of the LSPR effect for CuS NPs, and the NPs’ absorption characteristics could be tailored by adjusting the reduction potential of the Cu(II) pre‐SSP for bespoke applications.

### 2.9 Hydrothermal Decomposition of Cu(I) SSPs: Monitoring CuS Growth via NIR Spectroscopy

**FIGURE 8:**
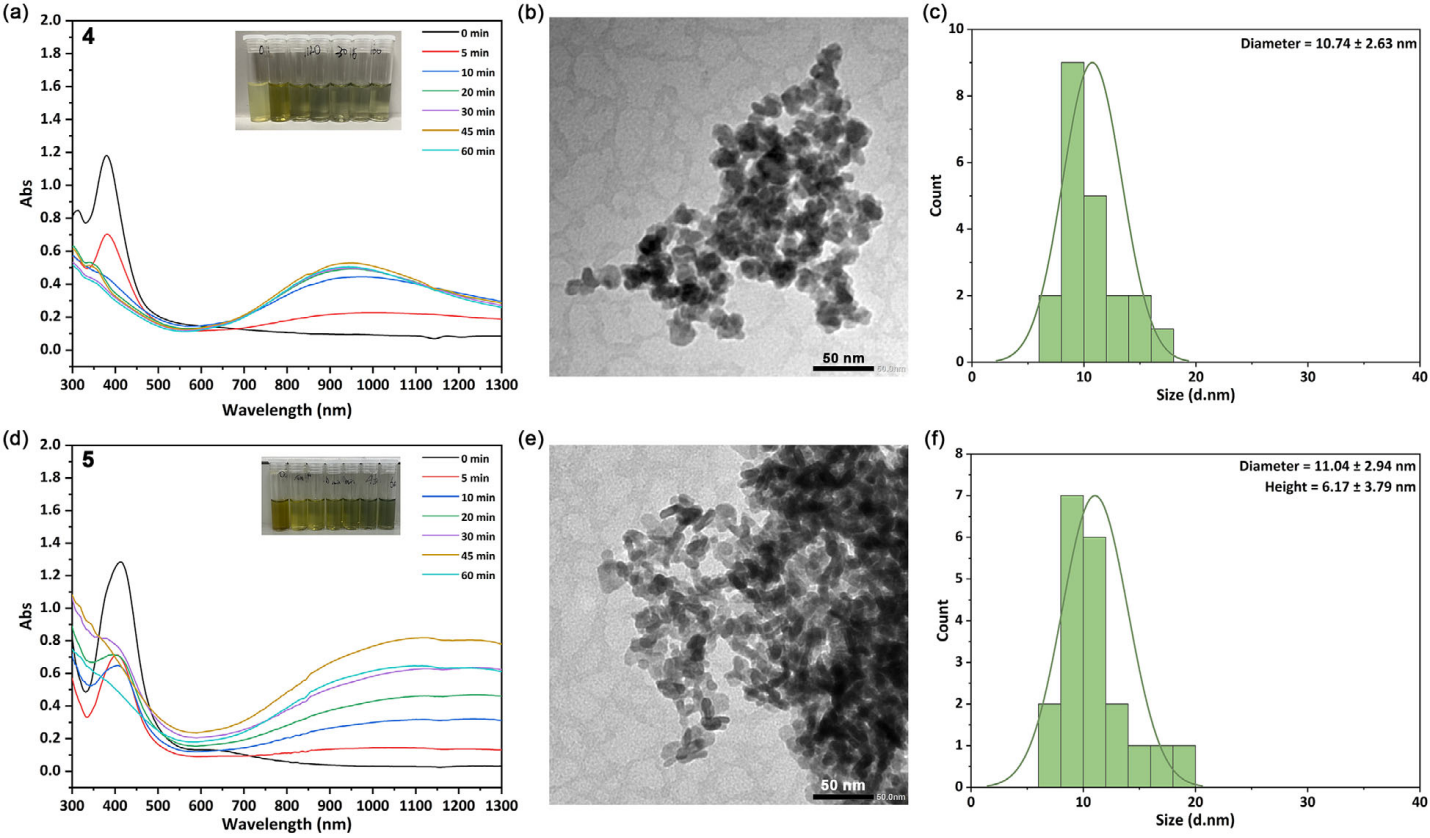
Decomposition of Cu(I)‐DTCs at 90°C: (a) UV–vis‐NIR spectra recording the decomposition of **4**; (b) TEM image of the NPs from the decomposition of **5**; (c) size distribution of the NPs from the decomposition of **4**; (d) UV–vis‐NIR spectra recording the decomposition of **5**; (e) TEM image of the NPs from the decomposition of **5**; (f) size distribution of the NPs from the decomposition of **5**.

Having shown that it is the reduction of Cu(II) to Cu(I) that is product‐determining for Cu(II) SSPs, we next turned our attention to the hydrothermal decomposition of Cu(I)‐DTC SSPs **4** and **5**. As with the Cu(II) work, these were carried out by hot‐injection into H_2_O at 90°C, and we also measured the growth of UV–vis‐NIR absorbances, which relate to the formation of CuS NPs (Figure 8a,d). Both **4** and **5** have low water‐solubility at ambient temperature, but the elevated temperature facilitates their dissolution, enabling the hydrothermal decomposition at 90°C. After heating for 1 h, both gave green suspensions shown to be CuS (covellite, JCPDS No. 06–0464) by PXRD (Figure S41). Concomitant with loss of the yellow coloration, associated with decomposition of the SSP, is the growth of a strong NIR absorbance peak at ca. 950 nm, which reaches a maximum after ca. 45 min, suggesting complete decomposition of the SSPs (Figure 8a,d). In contrast to the relatively slow decomposition of **1‐H** (4 h), decompositions of **4–5** are notably faster, further supporting our premise that Cu(II)‐Cu(I) reduction is rate‐limiting for the Cu(II) SSPs. TEM images (Figure 8b,e) shows that they give both smaller NPs as compared to the Cu(II) SSPs, and with a much narrower dispersity. Decomposition of **4** gave irregular NPs with an average size of 10.7 ± 2.6 nm (Figure 8b,c), while **5** gave nano‐disks with an average diameter of 11.0 ± 2.9 nm and a height of ca. 6 nm (Figure 8e,f). Thus, the significantly faster decomposition of Cu(I) SSPs leads to smaller and less dispersed CuS NPs.

### 2.10 Mechanistic Considerations

**SCHEME 3:**
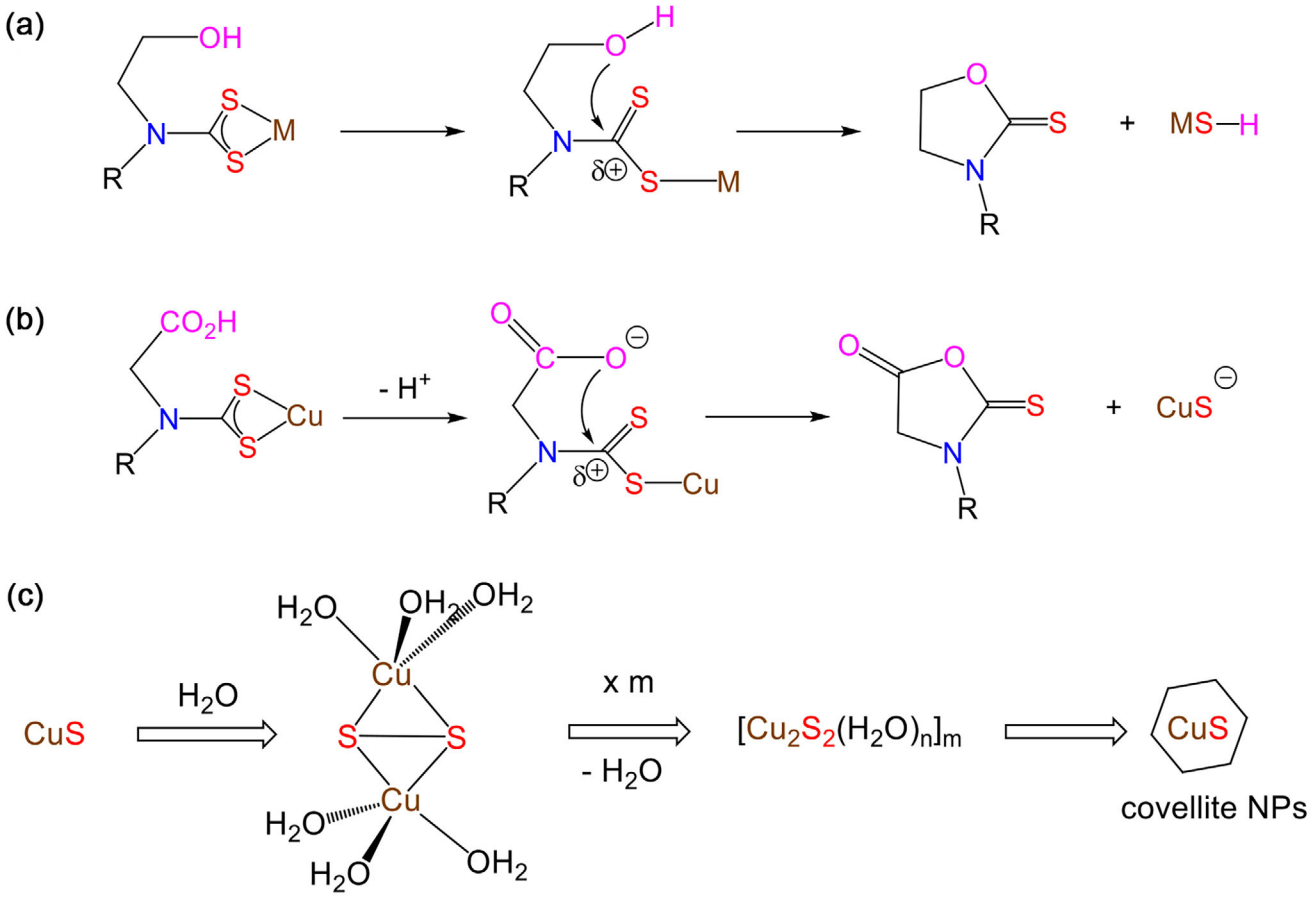
Proposed mechanism for the decomposition of (a) diethanol‐amine derived DTC complexes, (b) **4** and **5**, (c) proposed formation of covellite from Cu(I) bridging disulfide clusters.

It is striking that **4** and **5** are stable up to ca. 170°C in the solid state (Figure S28) but decompose to afford CuS at room temperature in water, a process that is fast at 90°C (Figure 8). This can be attributed to the molecular decomposition mechanism(s) (Scheme 3a,b), which are not available to simple dialkyl‐ or diaryl‐functionalized DTC complexes.

Thus, it has previously been established that diethanol‐amine derived DTC complexes decompose via an intramolecular nucleophilic attack at the electrophilic backbone carbon center (Scheme 3a), generating oxazolidine‐2‐thiones as the co‐product, which in some instances have been isolated and fully characterized [69, 70, 71, 72]. A similar process is proposed for **4** and **5** (Scheme 3b) to yield 3‐alkyl‐2‐thioxo‐5‐oxazolidinones, the greater acidity of the carboxylate group (*vs* hydroxide) leading to a thermodynamically favorable process. We have made no attempt to isolate the secondary organic product and note that such species are likely prone to hydrolysis. Importantly, the introduction of the CH_2_CO_2_H functionality on the backbone of the DTC both enhances water‐solubility, allowing rare examples of water‐soluble SSPs, but also provides a low‐energy decomposition pathway that allows NPs to be generated at significantly lower temperatures than those with non‐functionalized backbone substituents.

While we have not directly studied post‐molecular decomposition pathways leading to the aggregation of CuS monomers to form clusters and then the isolated NPs, we briefly comment on these here. Covellite can be formed from the reaction of CuSO_4_ and Na_2_S in water, and mechanistic aspects of this have been studied by Luther and co‐workers [73]. Here, Cu(II)‐containing sulfide clusters [Cu(µ‐S)(H_2_O)_2_]_3_ are proposed to initially form and then condense to give larger molecular building blocks. Importantly, reduction of Cu(II) to Cu(I), which is critical for covellite production, occurs *after* these initial cluster formations. In our process, reduction of Cu(II) to Cu(I) occurs *before* DTC decomposition and thus the small aqueous clusters initially generated contain Cu(I). Consequently, their level of hydration is likely to be much reduced as water binds more strongly to Cu(II) than Cu(I). Thus, while seemingly similar aqueous synthetic routes, they must occur via very different pathways and intermediates. We can only hypothesize on the nature of the Cu(I)‐sulfide cluster building blocks generated via DTC decomposition, but as the Cu:S ratio is 1:1, it is tempting to suggest that they contain Cu_2_S_2_ subunits, in which two Cu(I) centers are bridged by a disulfide (Scheme 3c). Such species are well‐known [74, 75, 76, 77, 78, 79], for example, the reaction of the tris(pyrazolyborate) complex [{κ^3^‐HB(3,5‐Pr^i^_2_pz)_3_}Cu(CO)] with S_8_ affords [{κ^3^‐HB(3,5‐Pr^i^_2_pz)_3_}_2_Cu_2_(µ‐κ^2^, κ^2^‐S_2_)] [75]. Thus, in aqueous solution, binuclear fragments such as [Cu_2_(µ‐κ^2^, κ^2^‐S_2_)(H_2_O)_6_] are easily invoked, which, in turn, could condense via loss of water and form larger [Cu_2_S_2_]_m_ building blocks en‐route to the isolated covellite NPs (Scheme 3c).

## 3 Conclusions

In summary, we have established the hydrothermal decomposition of easily prepared water‐soluble copper‐dithiocarbamates, Cu(II)‐DTC, as single‐source precursors for the controlled synthesis of technologically important CuS (covellite) nanomaterials. Importantly, nanoparticle tuneability is linked to a reduction‐controlled molecular decomposition pathway, which is rate‐limiting and sensitive to changes in both pH and ligand metalation. Thus, controlling the rate of intramolecular ligand‐to‐metal charge‐transfer, which can be easily followed by UV–vis spectroscopy, and concomitant formation of Cu(I)‐DTC species, which decompose rapidly, allows the rate of formation of reactive monomers to be controlled, in turn controlling nucleation and growth processes, and nanoparticle size and morphology. Via judicious choice of reaction conditions, the Cu(I)‐DTC intermediates can be isolated and fully characterized as phosphine adducts. While being significantly less water‐soluble than their Cu(II) counterparts, they also undergo hydrothermal decomposition at 90°C (or below), the rapid nature of which leads to the formation of small CuS nanomaterials, allowing their generation to be followed via their localized surface plasmon resonance in the near‐IR spectrum. Thus, the same copper‐ligand combinations can be utilized to generate a wide range of size‐shape tunable CuS nanomaterials in a cost‐effective and sustainable manner. Importantly, these nanomaterials are not capped by strongly bound surfactants and thus can be easily surface modified for biomedical applications, aspects of which we are developing in our current work, and which will be reported in due course.

## Author Contributions

X.X. contributed to data curation, formal analysis, investigation, methodology, validation, and writing the original draft, as well as writing through review and editing. S.H. contributed to data curation, investigation, and validation. D.A.K. contributed to data curation, investigation, methodology, and validation. T.J.C. contributed to data curation, investigation, and validation. Y.X. contributed to data curation, investigation, and validation. J.C.S. contributed to data curation, investigation, and validation. D.P. contributed to data curation and validation and participated in writing through review and editing. C.A.D. contributed to funding acquisition, resources, supervision, and writing through review and editing. G.H. contributed to conceptualization, data curation, formal analysis, funding acquisition, investigation, methodology, project administration, resources, supervision, visualization, and writing the original draft, as well as writing through review and editing.

## Conflicts of Interest

The authors declare no conflicts of interest.

## Supporting information




**Supporting File**: smll73141‐sup‐0001‐SuppMat.docx.

## Data Availability

The data that support the findings of this study are available in the supplementary material of this article.
