# Supplementary material for: Reduction‐Controlled Tunable Synthesis of Covellite (CuS) Nanoparticles from Water‐Soluble Single‐Source Precursors
Source: Small. 2026 Mar 18;22(27):e14339. doi: 10.1002/smll.202514339 (PMC13173312; doi:10.1002/smll.202514339)

**Supporting Information**

**Reduction-Controlled Tunable Synthesis of Covellite (CuS) Nanoparticles from Water-Soluble Single-Source Precursors**

*Xiang Xu**,^[a,b]^ Siqiao Huang,^[a]^ Desmond A. Koomson,^[a]^ Tannith-Jade Cole,^[a]^ Yuanyang Xie,^[c]^ Jagodish C. Sarker,^[d]^ David Pugh,^[a]^ Cécile A. Dreiss,^[b]^ and Graeme Hogarth^[a]*^*

[a] Department of Chemistry, King's College London, Britannia House, 7 Trinity Street, London SE1 1DB, UK

[b] Institute of Pharmaceutical Sciences, King's College London, Franklin Wilkins Building, Stamford Street, London SE1 9NH, UK

[c] Department of Physics and London Centre for Nanotechnology, King's College London, London, WS2R 2LS, UK

[d] Department of Chemistry, Jagannath University, Dhaka 1100, Bangladesh

Table of Contents

1. Experimental Section 4

**1.1** **General Information** 4

**1.2** **Synthesis of [NaS_2_CN(CH_2_CO_2_Na)_2_] (Imd-Na-DTC)** 5

**1.3** **Synthesis of [Cu{κ^2^-S_2_CN(CH_2_CO_2_Na)_2_}_2_]** (**1-Na**) 5

**1.4** **Synthesis of [Cu{κ^2^-S_2_CN(CH_2_CO_2_H)_2_}_2_]** (**1-H**) 6

**1.5** **Synthesis of [NaS_2_CNMeCH_2_CO_2_Na] (Sar-Na-DTC)** 6

**1.6** **Synthesis of [Cu{κ^2^-S_2_CNMe(CH_2_CO_2_Na)}_2_] (2-Na)** 6

**1.7** **Synthesis of [Cu{κ^2^-S_2_CNMe(CH_2_CO_2_H)}_2_] (2-H)** 7

**1.8** **Synthesis of [Cu{κ^2^-S_2_CN(CH_2_CH_2_OH)_2_}_2_] (3)** 7

**1.9** **Saturated Solubility Measurement** 7

**1.10** **Isolation of [Cu{κ^2^-S_2_CN(CH_2_CO_2_H)_2_}]_n_ (4) and [Cu{κ^2^-S_2_CNMe(CH_2_CO_2_H)}]_n_ (5)** 8

**1.11** **Synthesis of 4 and 5 using Dithiothreitol** 9

**1.12** **Synthesis of [Cu{κ^2^-S_2_CN(CH_2_CO_2_H)_2_}(PPh_3_)_2_] (4-PPh_3_) and [Cu{κ^2^-S_2_CNMe(CH_2_CO_2_H)}(PPh_3_)_2_] (5-PPh_3_)]** 9

**1.13** **Room Temperature Decomposition via UV-Vis-NIR** 10

**1.14** **Cyclic Voltammetry** 10

**1.15** **Effect of Temperature on the Decomposition of 1-Na** 11

**1.16** **Decomposition of Cu(II)-Imd DTCs at Different pH via Hot-Injection** 11

**1.17** **Decomposition of Cu(II)-Sar DTCs at Different pH via Hot-Injection** 12

**1.18** **Decomposition of Cu(II) Complexes with Different Cations via Hot-Injection** 12

**1.19** **Time-dependent UV-Vis-NIR Spectra of Hot Decomposition** 13

**1.20** **Decomposition of Cu(I)-DTC Complex via Hot-Injection** 13

2. Supplementary Results 14

**Figure S1.** IR spectra of (a) Imd-derived DTCs and (b) Sar-derived DTCs 14

**Figure S2.** IR spectrum of **3** 15

**Figure S3.** (a) Saturated solubility chart of SSPs in H_2_O at different pH; (b) Saturated solubility curve of **3** in H_2_O at different pH (NaOH and HCl were used to adjust the pH). Temperature = 25 ℃. n=3, data are presented as mean ± standard deviation 15

**Figure S4.** ^1^H NMR (400 MHz) spectrum of Imd-Na-DTC in D_2_O 16

**Figure S5.** ^13^C{^1^H} NMR (400 MHz) spectrum of Imd-Na-DTC in D_2_O 16

**Figure S6.** ^1^H NMR (400 MHz) spectrum of Sar-Na-DTC in D_2_O 17

**Figure S7.** ^13^C{^1^H} NMR (400 MHz) spectrum of Sar-Na-DTC in D_2_O 17

**Figure S8.** ESI(-)-MS spectrum of Imd-Na-DTC in H_2_O. *m/z* = 251.942, [S_2_CN(CH_2_CO_2_)_2_Na_2_]^-^ 18

**Figure S9.** ESI(-)-MS spectrum of Sar-Na-DTC in MeOH. *m/z =* 185.977, [S_2_CNMeCH_2_CO_2_Na]^-^ 18

**Figure S10.** ESI(-)-MS spectrum of **1-Na** in H_2_O 19

**Figure S11.** ESI(+)-MS spectrum of **1-H** in MeOH 19

**Figure S12.** ESI(-)-MS spectrum of **2-Na** in MeOH 20

**Figure S13.** ESI(+)-MS spectrum of **2-H** in MeOH 20

**Figure S14**. (a) Plots showing the decrease of Abs_430_ as a function of time (days) at various concentrations of **1-H** (25 ℃), ns: not significantly different; (b) Images showing the reduction of **1-H** after being wrapped with foil 21

**Figure S15.** ^1^H NMR (400 MHz) spectrum of the decomposition residue from **1-Na** in D_2_O 21

**Figure S16.** ^13^C{^1^H} NMR (400 MHz) spectrum of the decomposition residue from **1-Na** in D_2_O 22

**Figure S17.** ^1^H NMR (400 MHz) spectrum of the decomposition residue from **2-Na** in D_2_O 22

**Figure S18.** ^13^C{^1^H} NMR (400 MHz) spectrum of the decomposition residue from **2-Na** in D_2_O 23

**Figure S19.** ESI(-)-MS spectrum of the isolated **4** in CH_3_CN 23

**Figure S20.** ESI(-)-MS spectrum of the isolated **5** in MeOH 24

**Figure S21.** IR spectra of synthesized (a) **4-PPh_3_** and (b) **5-PPh_3_** 24

**Figure S22.** ^1^H NMR (400 MHz) spectrum of **4-PPh_3_** in CDCl_3_ 25

**Figure S23.** ^13^C{^1^H} NMR (400 MHz) spectrum of **4-PPh_3_** in CDCl_3_ 25

**Figure S24.** ^31^P{^1^H} NMR (400 MHz) spectrum of **4-PPh_3_** in CDCl_3_ 26

**Figure S25.** ^1^H NMR (400 MHz) spectrum of **5-PPh_3_** in DMSO-*d_6_* 26

**Figure S26.** ^13^C{^1^H} NMR (400 MHz) spectrum of **5-PPh_3_** in DMSO-*d_6_* 27

**Figure S27.** ^31^P{^1^H} NMR (400 MHz) spectrum of **5-PPh_3_** in DMSO-*d_6_* 27

**Figure S28.** Room temperature decomposition of Cu(II)-DTCs in H_2_O: UV-Vis-NIR spectra showing the absorbance change against time and inserted images show the color change, (a) **1-H** (pH 2.9); (b) **1-Na** (pH 7.8); (c) **1-Na** (pH 11.0); (d) **2-Na** (pH 7.1); (e) **2-Na** (pH 10.4). 28

**Figure S29**. PXRD patterns of (a) **4** and (b) **5** isolated from the heating method. CuS (Covellite, JCPDS No. 06-0464) is used as a reference 29

**Figure S30.** Size distribution of NPs obtained from **1-Na** (pH 7.8) at different temperatures. NPs from 90 ℃ are presented in **Figure S31 (1-Na** pH 7.8**)**. Microwave-assisted decompositions were abbreviated as “mw” 30

**Figure S31.** Decomposition studies of **1** at different pH: UV-Vis-NIR spectra and size distribution (90 ℃) 31

**Figure S32.** Decomposition studies of **1** with different cations: UV-Vis-NIR spectra and size distribution (90 ℃) 32

**Figure S33.** Decomposition studies of **2** at different pH: UV-Vis-NIR spectra and size distribution (90 ℃) 33

**Figure S34.** Decomposition studies of **2** with different cations: UV-Vis-NIR spectra and size distribution (90 ℃) 34

**Figure S35.** PXRD patterns of NPs obtained from **1-Na** at different temperatures. Microwave-assisted decompositions were abbreviated as “mw” 35

**Figure S36.** PXRD patterns of NPs obtained from **1** at different pH (90 ℃) 35

**Figure S37.** PXRD patterns of NPs obtained from **1** with different alkali cations (90 ℃) 36

**Figure S38.** PXRD patterns of NPs obtained from **2** at different pH (90 ℃) 36

**Figure S39.** PXRD patterns of NPs obtained from **2** with different cations (90 ℃) 37

**Figure S40.** PXRD patterns of NPs from decomposing (a) **4** and (b) **5** at 90 ℃. CuS (Covellite, JCPDS No. 06-0464) is used as a reference 37

**Table S1.** Selected IR band positions ν (in cm^-1^) and the decomposition temperatures for DTCs and Cu-DTC precursors 38

**Table S2.** Magnetic susceptibility table of Cu-DTC complexes 39

**Appendix** 1

# Experimental Section

- 1. **General Information**

All reagents and solvents were purchased from Sigma Aldrich or Flurochem and used without further purification. H_2_O used was deionized water (18.2 MΩ·cm), purified by Duo™ II.I Two-In-One Water Purification System.

**Nuclear Magnetic Resonance Spectra** (NMR): ^1^H NMR, ^13^C{^1^H} NMR, and ^31^P{^1^H} NMR spectra were recorded on a Bruker Avance III 400 MHz spectrometer at ambient temperature, and chemical shifts are reported in ppm with the solvent resonance as the internal standard (D_2_O, DMSO-*d_6_*, or CDCl_3_).

**Attenuated Total Reflectance Fourier Transform Infrared Spectroscopy** (ATR-FTIR) analyses were conducted using a Bruker INVENIO R spectrometer equipped with a diamond ATR unit.

**High-Resolution Mass Spectrometry** (HRMS) was measured on a UPLC-Xevo G2 XS QToF High-Resolution Mass Spectrometer applying the ESI-positive and the ESI-negative modes.

**Powder X-ray Diffraction** (PXRD) spectra were recorded by a Bruker D8 Advance diffractometer with glass sample holders using Cu Kα1 radiation. Diffraction patterns obtained were compared to database standards.

**Transmission Electron Microscope** (TEM) images were obtained using a JEM-1400 Flash microscope at 100 kV, equipped with a tungsten filament and Matataki Flash high-sensitivity sCMOS camera (King’s Centre for Ultrastructural Imaging). Two 30 μL droplets of nanoparticle suspension (MeOH) were placed on a holey carbon-coated copper TEM grid and allowed to evaporate in air under ambient laboratory conditions. The size distribution based on TEM images was analyzed by the software Image J.

**Elemental Analysis** was conducted by London Metropolitan University with a FLASH 2000 CHNS/O Analyzer, equipped with a thermal conductivity detector.

**Ultraviolet-Visible-Near Infrared (UV-Vis-NIR)** **spectra** were recorded by a U4100 spectrometer, Hitachi High Technologies, water being used as the reference for all the samples.

**Thermal Gravimetric Analysis-Differential Scanning Calorimetry** (TGA-DSC) was conducted with the Discovery SDT-650, TA Instruments. Nitrogen was used as the protective gas and the compressed air for air-cooling.

**Magnetic Susceptibility** was measured by a Sherwood Scientific MK 1 Magnetic Susceptibility Balance.

**Crystallographic data** (Single Crystal X-ray) were collected by the EPSRC UK National Crystallography Service at the University of Southampton.

A Biotage Initiator+ microwave synthesizer was used for the microwave-assisted decomposition and the Beckman J6-MI from Beckman Coulter Ultracentrifuge (Rotor ID-25.50) was exploited for the separation of nanoparticles (NPs).

- 1. **Synthesis of [****NaS_2_CN(CH_2_CO_2_Na)_2_] (Imd-Na-DTC)**

NaOH (2.80 g, 70 mmol) and iminodiacetic acid (Imd, 2.66 g, 20 mmol) were dissolved in MeOH (100 mL) with vigorous stirring. After the decline of the exothermic reaction, CS_2_ (2.0 mL, 30 mmol) was added at room temperature, and stirring was continued for 2 h. MeOH was removed by rotary evaporation and a copious amount of EtOH (3×100 mL) was used to wash the product. After drying in vacuum, a pale white powder (Imd-Na-DTC) formed (2.92 g, 53%). Characterizing data: Anal. Calc. for [NaS_2_CN(CH_2_CO_2_Na)_2_].2H_2_O (Mw=311 g/mol): C, 19.29; H, 2.57; N, 4.5; S, 20.57. Found: C, 18.99; H, 2.67; N, 4.69; S, 18.55. IR (cm^-1^) 3539, 3436, 3311, 1594 (C=O), 1415 (C-N), 1384, 1311,1207 (C-S), 1170, 993, 952, 771, 727, 605, 555, 509.^1^H NMR (400 MHz, D_2_O): δ 4.66 (s, 2H, CH_2_). ^13^C{^1^H} NMR (D_2_O): ẟ 211.69 (-CSS-), 176.54, 59.16, 57.46. ESI(-)-MS in H_2_O: *m/z* = 251.942, [S_2_CN(CH_2_CO_2_)_2_Na]^-^.

- 1. **Synthesis of [Cu{κ^2^-S_2_CN(CH_2_CO_2_Na)_2_}_2_]** (**1-Na**)

To a solution of Imd-Na-DTC (2.75 g, 10 mmol) in MeOH (100 mL), CuSO_4_·5H_2_O (1.25 g, 5 mmol) in H_2_O (5 mL) was added dropwise with vigorous stirring. A dark brown precipitate formed immediately, and it was further stirred for 30 min. After vacuum filtration, the solid was washed successively with cold MeOH (3×30 mL) and dried in vacuum at 40 ℃ overnight to produce **1-Na** (2.32 g, 82%). Characterizing data: Anal. Calc. for [Cu{κ^2^-S_2_CN(CH_2_CO_2_Na)_2_}_2_].4H_2_O (Mw=639 g/mol): C, 18.77; H, 2.50; N, 4.38; S, 20.03. Found: C, 16.14; H, 1.91; N, 3.20; S, 20.68. IR (cm^-1^) 1590, 1484 (C-N), 1390, 1333, 1305, 1263, 1213 (C-S), 1105, 999, 957, 912, 810, 728, 621, 553, 482. µ_eff_: 1.42 BM.

- 1. **Synthesis of [Cu{κ^2^-S_2_CN(CH_2_CO_2_H)_2_}_2_]** (**1-H**)

To a solution of Imd-Na-DTC (2.76 g, 10 mmol) in H_2_O (15 mL), CuSO_4_·5H_2_O (1.25 g, 5 mmol) in H_2_O (5 mL) was added dropwise with vigorous stirring. The solution immediately turned dark brown, and it was further stirred for 30 min, followed by adding HCl (3 M) until the supernatant was clear. The solid was separated via centrifugation (4300 ×g, 10 min) and washed with ice-cold H_2_O (3×30 mL) before being freeze-dried for 48 h, affording dark brown **1-H** (1.69 g, 70%). Characterizing data: Anal. Calc. for [Cu{κ^2^-S_2_CN(CH_2_CO_2_H)_2_}_2_] (Mw=479 g/mol): C, 24.95; H, 2.70; N, 5.82; S, 26.61. Found: C, 24.00; H, 2.71; N, 5.31; S, 26.12. IR (cm^-1^) 3135 (O-H), 2924 (-CH_2_-), 2621, 2534((CO)O-H), 1724 (C=O), 1686, 1642, 1486 (C-N), 1457, 1386, 1360, 1332, 1296, 1234, 1203 (C-S), 1175, 1028, 956, 884, 812, 698, 627, 537. µ_eff_: 1.39 BM. ESI(+)-MS in MeOH: *m/z* 478.88 ([Cu(S_2_CNC_2_H_4_C_2_O_4_H_2_)_2_]^+^);

- 1. **Synthesis of [****NaS_2_CNMeCH_2_CO_2_Na] (Sar-Na-DTC)**

NaOH (2.80 g, 70 mmol) and sarcosine (Sar, 1.78 g, 20 mmol) were dissolved in MeOH (100 mL) with vigorous stirring. After the decline of the exothermic reaction, CS_2_ (2.0 mL, 30 mmol) was added at room temperature, and stirring was continued for 2 h. MeOH was removed by rotary evaporation, and a copious amount of acetone was used to wash the product until the acetone became colorless. After drying in a vacuum, a pale white powder (Sar-Na-DTC) formed (1.58 g, 38%). Characterizing data: Anal. Calc. for [NaS_2_CNMeCH_2_CO_2_Na].2H_2_O (Mw=246 g/mol): C, 19.51; H, 3.66; N, 5.69; S, 26.02. Found: C, 19.29; H, 3.70; N, 5.50; S, 26.80. IR (cm^-1^) 3492, 3311, 2929, 2096, 1589 (C=O), 1481, 1402 (C-N), 1396, 1253, 1193 (C-S), 1089, 997, 956, 730, 609, 551, 486, 430.^1^H NMR (400 MHz, D_2_O): δ 4.68 (s, 2H, CH_2_), δ 3.48 (s, 3H, CH_3_). ^13^C{^1^H} NMR (D_2_O): ẟ 209.70 (-CSS-), 176.37, 60.48, 44.04. ESI(-)-MS spectrum in MeOH: *m/z =* 185.977, [S_2_CNMeCH_2_CO_2_Na]^-^

- 1. **Synthesis of [Cu{κ^2^-S_2_CNMe(CH_2_CO_2_Na)}_2_] (2-Na)**

To a solution of Sar-Na-DTC (2.10 g, 10 mmol) in MeOH (20 mL), CuSO_4_·5H_2_O (1.25 g, 5 mmol) in H_2_O (5 mL) was added dropwise with vigorous stirring. A dark brown precipitate formed immediately, and it was further stirred for 30 min. After vacuum filtration, the solid was washed successively with ice-cold MeOH (3×30 mL) and dried in vacuum at 40 ℃ overnight to give **2-Na** (1.28 g, 59%). Characterizing data: Anal. Calc. for [Cu{κ^2^-S_2_CNMe(CH_2_CO_2_Na)}_2_].4H_2_O (Mw=508 g/mol): C, 18.89; H, 3.54; N, 5.51; S, 25.19. Found: C, 17.64; H, 3.34; N, 4.68; S, 24.42. IR (cm^-1^) 3556, 3402, 3122, 2920, 1610 (C=O), 1519, 1403 (C-N), 1374, 1296,1213 (C-S), 1098, 969, 901, 775, 705, 575, 500. µ_eff_: 1.16 BM.

- 1. **Synthesis of [Cu{κ^2^-S_2_CNMe(CH_2_CO_2_H)}_2_] (2-H)**

To a solution of Sar-Na-DTC (2.10 g, 10 mmol) in H_2_O (15 mL), CuSO_4_·5H_2_O (1.25 g, 5 mmol) in H_2_O (5 mL) was added dropwise with vigorous stirring. The solution immediately turned dark brown, and it was further stirred for 30 min, followed by adding HCl (3 M) until the supernatant was clear. The solid was separated via centrifugation (4300 ×g, 10 min) and washed with ice-cold H_2_O (3×30 mL) before being freeze-dried for 48 h, resulting in dark brown **2-H** (1.84 g, 94%). Characterizing data: Anal. Calc. for [Cu{κ^2^-S_2_CNMe(CH_2_CO_2_H)}_2_] (Mw=392 g/mol): C, 24.49; H, 3.06; N, 7.14; S, 32.65. Found: C, 23.37; H, 3.04; N, 6.69; S, 31.83. IR (cm^-1^) 3566, 2922 (-CH_2_-), 2494((CO)O-H), 2116, 1710 (C=O), 1550, 1499 (C-N), 1394, 1277, 1207 (C-S), 1099, 1010, 967, 872, 680, 607, 495. µ_eff_: 0.88 BM. ESI(+)-MS in MeOH: *m/z* 390.72 ([Cu(S_2_CNC_2_H_5_CO_2_H)_2_]^+^).

- 1. **Synthesis of** **[Cu{κ^2^-S_2_CN(CH_2_CH_2_OH)_2_}_2_] (3)**

NaOH (1.20 g, 30 mmol) and diethanolamine (2.12 g, 20 mmol) were dissolved in EtOH (100 mL) with vigorous stirring. After the decline of the exothermic reaction, CS_2_ (2.0 mL, 30 mmol) was added at room temperature, and stirring was continued for 2 h. To this stirred solution, CuSO_4_·5H_2_O (2.5 g, 10 mmol) in H_2_O (10 mL) was then added dropwise. The solution immediately turned dark brown, and it was further stirred for 30 min, followed by adding HCl (3 M) until the pH turned 7~8. After vacuum filtration, the brown solid was collected and washed with ice-cold EtOH (3×30 mL) and dried in vacuum at 40 ℃ overnight to give **3** (2.56 g, 60%). Characterizing data: IR (cm^-1^) 3223, 2931, 2872, 2118, 1722, 1487, 1406, 1350 (C-N), 1215 (C-S), 1051, 991, 889, 613.

- 1. **Saturated Solubility Measurement**

The UV-Vis standard curve method was exploited to quantify the saturated solubility of Cu(II)-DTCs. The standard solutions at pre-determined concentrations for **1-H**, **2-Na**, and **3** were prepared in MeOH with volumetric flasks and measured for absorbance at 430 nm by a UV-Vis spectrometer. Respective standard curves were graphed and fitted in Origin.

To quantify the saturated solubility, 20 mg of each compound (**1-H**, **2-H**, and **3**) was separately dispersed in 5 mL of deionized water with different pH (pH was adjusted with NaOH or HCl), and stirred for 24 h at 25 ℃. After being filtered through a 0.22 µm cellulose membrane, the obtained solution was measured for absorbance at 430 nm, followed by calculating the saturated solubility via the corresponding standard curves.

- 1. **Isolation of [Cu{κ^2^-S_2_CN(CH_2_CO_2_H)_2_}]_n_ (4) and [Cu{κ^2^-S_2_CNMe(CH_2_CO_2_H)}]_n_ (5)**

A concentrated suspension of **1-H** (4 mg/mL, 50 mL) was heated at 80 ℃ for 2 h under stirring, resulting in a yellow suspension. The yellow solid was then collected by centrifugation (4300 ×g, 10 min), washed with cold MeOH (3 × 50 mL), and finally freeze-dried for 48 h to give **4**. Characterizing data: IR (cm^-1^) 3434 2938, 2109, 1907, 1712 (C=O), 1519, 1472, 1387 (C-N), 1206 (C-S), 1167, 939, 877, 717, 592, 526. ESI(-)-MS in CH_3_CN: *m/z* 269.839 [Cu{κ^2^-S_2_CN(CH_2_CO_2_)_2_H}]^-^.

**2-H** is sparingly soluble in H_2_O, and **2-Na** was thus used instead to extract the intermediate. First, a concentrated **2-Na** solution (4 mg/mL, 50 mL) was heated at 80 ℃ for 4 h with stirring, and then it was cooled to ambient temperature and stirred for another 3 d to give a light brown suspension (it is important to avoid heating the **2-Na** solution for too long as it decomposes into CuS). The pH was adjusted to 2~3 by slowly adding 1 M HCl solution to protonate the carboxylate groups, and the subsequent precipitate was collected by centrifugation (4300 ×g, 10 min), washed with cold MeOH (3 × 50 mL), and finally freeze-dried for 48 h to give **5**. Characterizing data: IR (cm^-1^) 3458, 2931, 2106, 1897, 1726 (C=O), 1486, 1380 (C-N), 1195 (C-S), 1091, 948, 873, 688, 599,491, 435. ESI(-)-MS in MeOH: *m/z* 225.912 [Cu{κ^2^-S_2_CNMeCH_2_CO_2_}]^-^.

Attempts to get NMR spectra of **4** and **5** were unsuccessful as they have very poor solubility in D_2_O and DMSO-*d_6_*, and thus the corresponding PPh_3_ adducts were synthesized in **Section 1.12**.

- 1. **Synthesis of 4 and 5 using Dithiothreitol**

We first tried directly adding Cu(I) salt into DTC solutions under nitrogen to prepare **4** and **5**, but only mixtures of Cu(I)-DTCs and Cu(II)-DTCs were obtained, indicating that some oxidation occurs during complexation. We next screened a range of reducing agents and found that dithiothreitol (DTT) was reducing enough to reduce the Cu(II) centre. The synthesis is detailed below:

**1-H** (240 mg, ~0.5 mmol) was homogenously dispersed in 15 mL of HCl solution (1 M) by sonication, which gave a dark brown suspension. After purging with N_2_ for 5 min, a dithiothreitol solution (0.3 M, 2 mL) was added to the suspension, which was then stirred vigorously for 1 h under N_2_ atmosphere and converted into a yellow suspension. The product was isolated by centrifugation (4300 ×g, 5 min), and the resulting bright yellow precipitate was washed with deionized water (3 × 50 mL) and lyophilized for 48 h. Characterizing data: Anal. Calc. for [Cu{κ^2^-S_2_CNCH_2_(CO_2_H)_2_}].2H_2_O (Mw=308 g/mol): C, 19.48; H, 3.25; N, 4.55; S, 20.78. Found: C, 19.01; H, 2.17; N, 3.98; S, 20.84.

**2-H** (60 mg, ~0.15 mmol) was homogenously dispersed in 150 mL of HCl solution (1 M) by sonication, which gave a dark brown suspension. After purging with N_2_ for 5 min, a dithiothreitol solution (0.6 M, 2 mL) was added to the suspension, which was then stirred vigorously for 1 h under N_2_ atmosphere and converted into a yellow suspension. The product was isolated by centrifugation (4300 ×g, 5 min), and the resulting bright yellow precipitate was washed with deionized water (3 × 50 mL) and lyophilized for 48 h. Characterizing data: Anal. Calc. for Cu{κ^2^-S_2_CNMe(CH_2_CO_2_H)}.4H_2_O (Mw=291 g/mol): C, 16.49; H, 4.81; N, 4.81; S, 21.99. Found: C, 16.53; H, 2.63; N, 1.75; S, 22.02.

- 1. **Synthesis of [Cu{κ^2^-S_2_CN(CH_2_CO_2_H)_2_}(PPh_3_)_2_] (4-PPh_3_) and [Cu{κ^2^-S_2_CNMe(CH_2_CO_2_H)}(PPh_3_)_2_] (5-PPh_3_)]**

The intermediates (250 mg, Ca.1 mmol) isolated from **Section 1.10** were dispersed in MeOH (50 mL), and to this dispersion PPh_3_ (400 mg, ca. 1.5 mmol) was added, whereafter the mixture was stirred overnight. After centrifugation (4300 ×g, 10 min), washing with MeOH (3 × 50 mL), and drying atop the oven, the light yellow PPh_3_-adducts were collected. Characterizing data:

**4-PPh_3_**: 266.22 mg, yield = 67%. Anal. Calc. for [Cu{κ^2^-S_2_CNCH_2_(CO_2_H)_2_}(PPh_3_)_2_] (Mw=796 g/mol): C, 61.76; H, 4.68; N, 1.76; S, 8.04. Found: C, 62.72; H, 4.56; N, 1.34; S, 6.38. IR (cm^-1^) 3053, 2981, 1758, 1722 (C=O), 1583, 1479, 1432 (C-N), 1371, 1315 (C-S), 1182 (O-H), 1093, 1020, 744, 694, 507. ^1^H NMR (400 MHz, CDCl_3_): δ 4.28 (s, 2H, CH_2_), 7.35-7.60 (m, 35H, Ph). ^13^C{^1^H} NMR (D_2_O): ẟ 213.75 (-CSS-), 173.32, 133.84-128.49 (Ph), 58.59, 57.46. ^31^P{^1^H} NMR (CDCl_3_) δ -1.41.

**5-PPh_3_**: 223.05 mg, yield = 59%. Anal. Calc. for [Cu{κ^2^-S_2_CNMe(CH_2_CO_2_H)}(PPh_3_)_2_] (Mw=752 g/mol): C, 63.77; H, 4.95; N, 1.86; S, 8.51. Found: C, 61.82; H, 4.62; N, 1.66; S, 9.00. IR (cm^-1^) 3047, 2669, 1726 (C=O), 1701, 1475, 1432, 1375, 1328 (C-S), 1251, 1211 (O-H), 1093, 966, 742, 692, 628, 501. ^1^H NMR (400 MHz, DMSO-*d_6_*): δ 3.36 (s, 3H, CH_3_), 4.64 (s, 2H, CH_2_), 7.26-7.40 (m, 35H, Ph). ^13^C{^1^H} NMR (400 MHz, DMSO-*d_6_*): ẟ 210.46 (-CSS-), 170.13, 134.57-128.93 (Ph), 55.63, 41.88. ^31^P{^1^H} NMR (DMSO-*d_6_*) δ -2.67.

- 1. **Room Temperature Decomposition via UV-Vis-NIR**

For the Imd-SSPs, 10 mg of **1-Na** was dissolved in 40 mL of deionized water, and the pH was adjusted to 11.0, 7.8, and 4.5 separately by 0.1 M NaOH or 0.1 M HCl. 10 mg of **1-H** was dissolved in 40 mL of deionized water to give the **1-H** (pH 2.9) solution. These solutions were sealed and stored at ambient temperature for 20 days. UV-Vis-NIR spectra were recorded after 0, 1, 2, 3, 4, 6, 8, 10, 12, 14, 16, 18, and 20 days.

For the Sar-SSPs, 10 mg of **2-Na** complexes were dissolved in 50 mL of deionized water, and the pH was adjusted to 10.4, 7.1, and 5.0 separately by 0.1 M NaOH or 0.1 M HCl. These solutions were sealed and stored at ambient temperature for 24 days. UV-Vis-NIR spectra were recorded after 0, 2, 4, 8, 12, 16, 20, and 24 days.

- 1. **Cyclic Voltammetry**

The Imd-SSPs solutions were prepared by dissolving **1-Na** (120 mg, ca. 0.2 mmol) in 40 mL of deionized water, followed by adjusting the pH to 11.0, 7.8, 4.5, and 2.9 with either 0.1 M NaOH or 0.1 M HCl. To **1-H** solutions LiOH·H_2_O (60 mg/mL, ca. 100 μL) and KOH (280 mg/mL, ca. 120 μL) were added separately to adjust the pH to neutral, affording the **1-Li** and **1-K** solutions.

The Sar-SSPs solutions were prepared by dissolving **2-Na** (100 mg, ca. 0.2 mmol) in 60 mL of deionized water, followed by adjusting the pH to 10.4, 7.1, and 5.0 with either 0.1 M NaOH or 0.1 M HCl. To **2-H** dispersions, LiOH·H_2_O (60 mg/mL, ca. 40 μL) and KOH (280 mg/mL, ca. 75 μL) were added separately to adjust the pH to neutral, affording the **2-Li** and **2-K** solutions.

Cyclic voltammograms (CV) were recorded using a three-electrode setup, including a glassy carbon working electrode, a platinum counter electrode, and a Ag/AgCl reference electrode (scan rate: 100 mV/s, 25 ℃). Each sample was made and measured in triplicate.

- 1. **Effect of Temperature on the Decomposition of 1-Na**

For comparison, the temperature effect was studied on **1-Na**.

**Hot-injection decomposition: 1-Na** (0.21 mmol) was dispersed in 20 mL of H_2_O to give a brown solution, which was then slowly injected into 40 mL of H_2_O at 80 ℃, 90 ℃, and 100 ℃, respectively, with stirring under N_2_. Aliquots of 200 μL were taken at different timepoints and diluted into 1800 μL of H_2_O for UV-Vis-NIR characterization. The reaction was then cooled to room temperature. NPs were isolated through the addition of an equal volume of MeOH and collected via centrifugation (22700 ×g, 10 min), followed by washing with H_2_O (2×50 mL) and MeOH (2×50 mL).

**Microwave-assisted decomposition**: In a microwave reaction kit (10-20 mL), **1-Na** (0.07 mmol) was dissolved in 20 mL of deionized water, forming a dark brown solution. The kit was heated by a microwave synthesizer (250 W) to 110, 120, and 140 ℃ for 20 min, respectively. The resulting NPs were collected and washed using the aforementioned procedures.

- 1. **Decomposition of Cu(II)-Imd DTCs at Different pH via Hot-Injection**

Precursor solutions with different pH were made as follows (pH here represents the final pH of the decomposition solution after injection):

- pH 11.0: **1-Na** (0.21 mmol) was dissolved in 20 mL of H_2_O to give a dark brown solution whose pH was then adjusted by adding 520 μL of 0.1 M NaOH solution.
- pH 7.8: **1-Na** (0.21 mmol) was dissolved in 20 mL of H_2_O to give a dark brown solution.
- pH 6.2: **1-Na** (0.21 mmol) was dissolved in 20 mL of H_2_O to give a dark brown solution whose pH was then adjusted by adding 1.2 mL of 0.1 M HCl solution.
- pH 4.5: **1-Na** (0.21 mmol) was dissolved in 20 mL of H_2_O to give a dark brown solution whose pH was then adjusted by adding 2.5 mL of 0.1 M HCl solution.
- pH 2.9: **1-H** (0.21 mmol) was dissolved in 20 mL of H_2_O to give a dark brown solution.

The above precursor solutions were prewarmed to ca. 70 ℃ and then slowly injected into 40 mL of 90 ℃ H_2_O separately, whereafter the temperature was maintained for 4 h under N_2_. The NPs were washed with H_2_O (2×50 mL) and MeOH (2×50 mL). All purified NPs were dried in an oven (80 ℃) overnight.

- 1. **Decomposition of Cu(II)-Sar DTCs at Different pH via Hot-Injection**

Precursor solutions with different pH were made as follows (pH here represents the final pH of the decomposition solution after injection):

- pH 10.4: **2-Na** (0.21 mmol) was dissolved in 20 mL of H_2_O to give a dark brown solution whose pH was then adjusted by adding 120 μL of 0.1 M NaOH solution.
- pH 7.1: **2-Na** (0.21 mmol) was dissolved in 20 mL of H_2_O to give a dark brown solution.
- pH 5.0: **2-Na** (0.21 mmol) was dissolved in 20 mL of H_2_O to give a dark brown solution whose pH was then adjusted by adding 240 μL of 0.1 M HCl solution.

The above precursor solutions were prewarmed to ca. 70 ℃ and then slowly injected into 40 mL of 90 ℃ H_2_O separately, whereafter the temperature was maintained for 4 h under N_2_. The resulting NPs were collected and washed using the aforementioned procedures.

- 1. **Decomposition of Cu(II) Complexes with Different Cations via Hot-Injection**

To **1-H** or **2-H** (0.21 mmol in 20 mL H_2_O) dispersions, LiOH·H_2_O (60 mg/mL) and KOH (280 mg/mL) were added separately to adjust the pH to 7~8, affording the **1-Li,** **1-K, 2-Li,** and **2-K** solutions. The individual precursor solution was then prewarmed to ca. 70 ℃ and slowly injected into H_2_O (40 mL, 90 ℃) with vigorous stirring under N_2_. After 4 h, the reaction was cooled to room temperature before isolating NPs through the addition of an equal volume of MeOH and collecting them via centrifugation (22700 ×g, 10 min). The NPs were washed with H_2_O (2×50 mL) and MeOH (2×50 mL). All purified NPs were dried in an oven (80 ℃) overnight.

- 1. **Time-dependent UV-Vis-NIR Spectra of Hot Decomposition**

For each hot-injection decomposition above, 200 μL of aliquots were taken at pre-determined time points and then diluted into 1.8 mL H_2_O, which also cooled and quenched the hot decomposition of the aliquots. The diluted samples were measured for the UV-Vis-NIR absorbance, using H_2_O as the reference, to graph the time-dependent spectra for each decomposition. Each decomposition was recorded in triplicate to calculate the kinetics.

- 1. **Decomposition of Cu(I)-DTC Complex via Hot-Injection**

**4-H** (30 mg, ca. 0.11 mmol) was dispersed in 5 mL of H_2_O to give a yellow suspension which was then slowly injected into 25 mL of 90 ℃ H_2_O with stirring under N_2_. Aliquots of 200 μL were taken and diluted into 1800 μL of H_2_O after 0, 5, 10, 20, 30, 45, and 60 min for UV-Vis-NIR characterization. The reaction was then cooled to room temperature before isolating NPs through the addition of an equal volume of MeOH and collecting them via centrifugation (22700 ×g, 10 min) followed by washing with H_2_O (2×50 mL) and MeOH (2×50 mL).

**5-H** (30 mg, ca. 0.13 mmol) was dispersed in 5 mL of H_2_O to give a light orange suspension which was then slowly injected into 25 mL of 90 ℃ H_2_O with stirring under N_2_. Aliquots of 200 μL were taken and diluted into 1800 μL of H_2_O after 0, 5, 10, 20, 30, 45, and 60 min for UV-Vis-NIR characterization. The reaction was then cooled to room temperature before isolating NPs through the addition of an equal volume of MeOH and collecting them via centrifugation (22700 ×g, 10 min) followed by washing with H_2_O (2×50 mL) and MeOH (2×50 mL).

# Supplementary Results


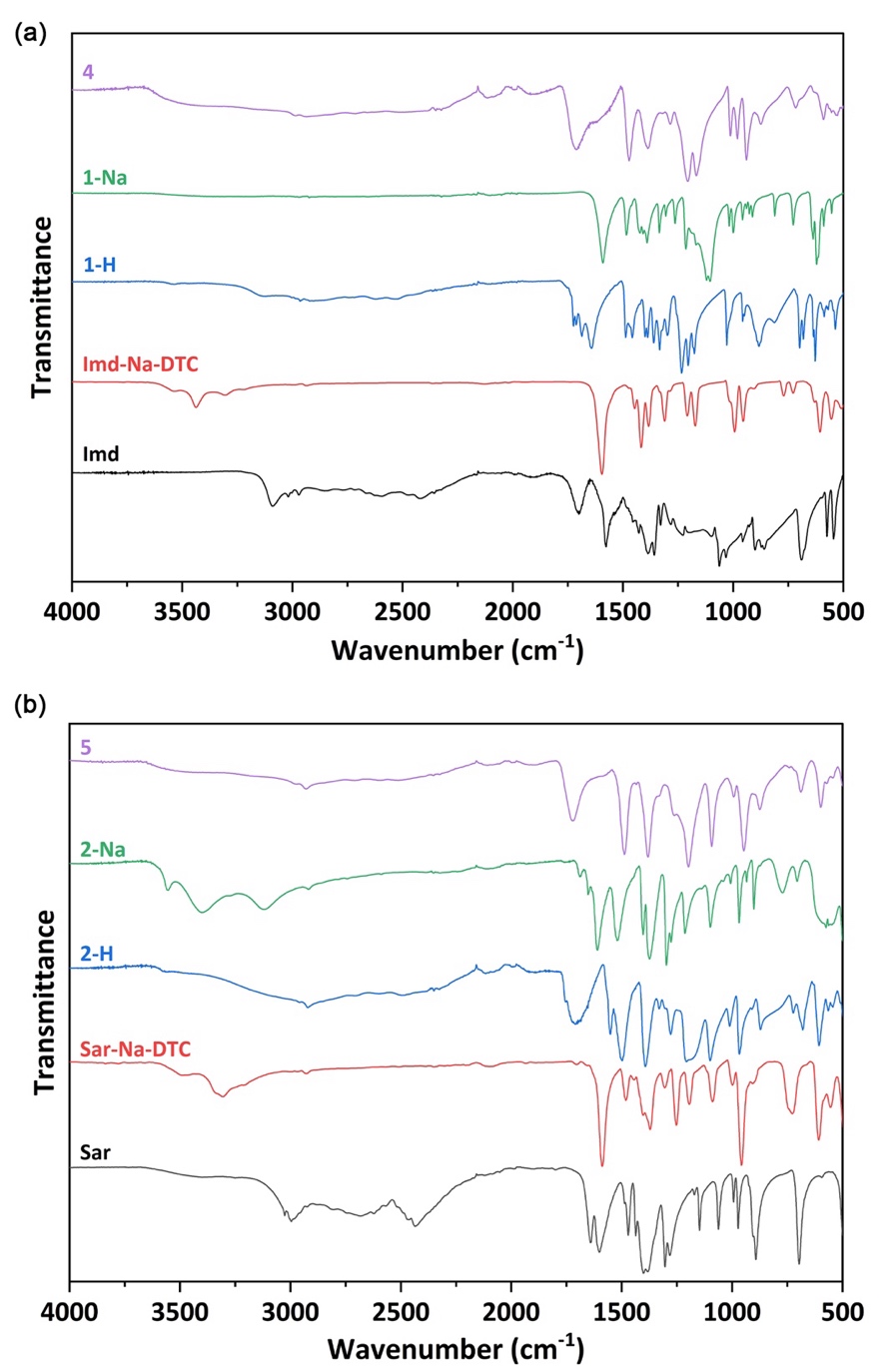


## **Figure S1.** IR spectra of (a) Imd-derived DTCs and (b) Sar-derived DTCs


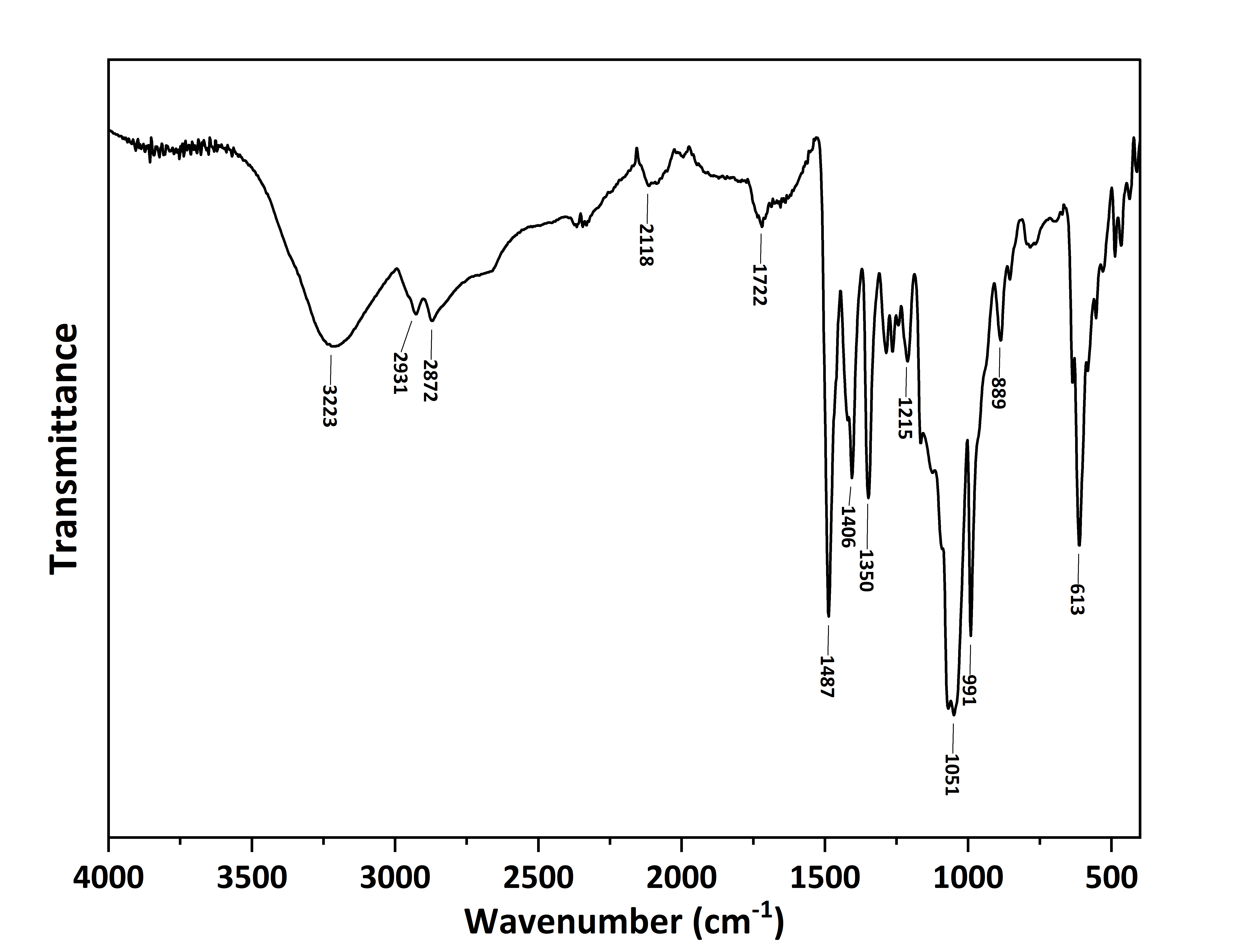


## **Figure S2.** IR spectrum of **3**


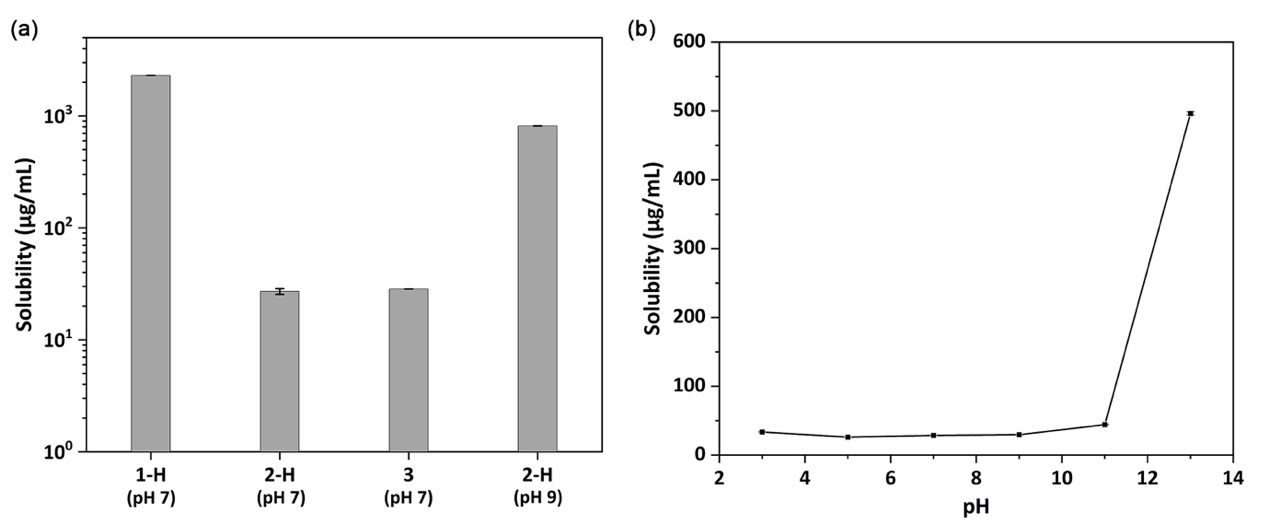


## **Figure S3.** (a) Saturated solubility chart of SSPs in H_2_O at different pH; (b) Saturated solubility curve of **3** in H_2_O at different pH (NaOH and HCl were used to adjust the pH). Temperature = 25 ℃. n=3, data are presented as mean ± standard deviation

**
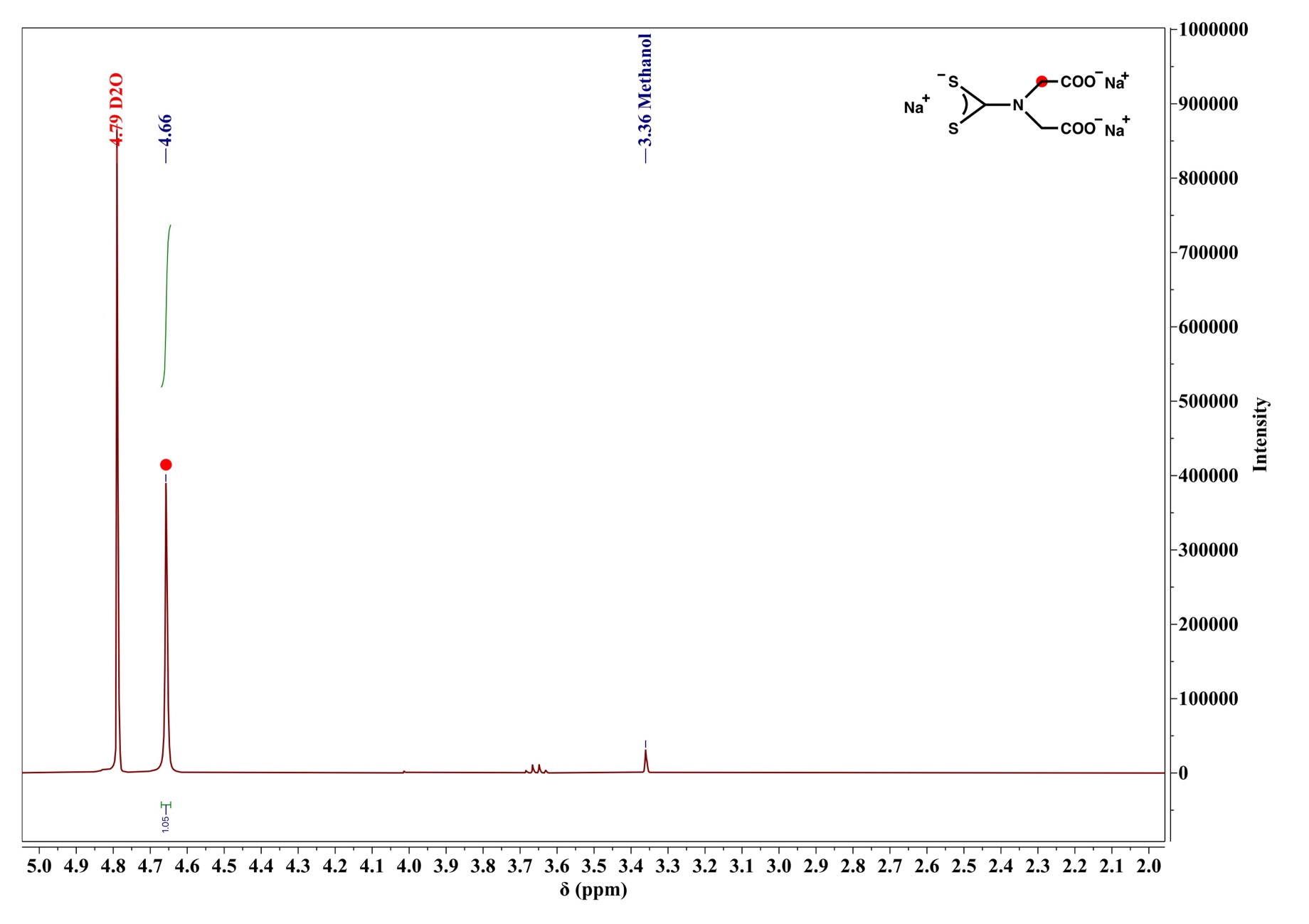
**

## **Figure S4.** ^1^H NMR (400 MHz) spectrum of Imd-Na-DTC in D_2_O

**
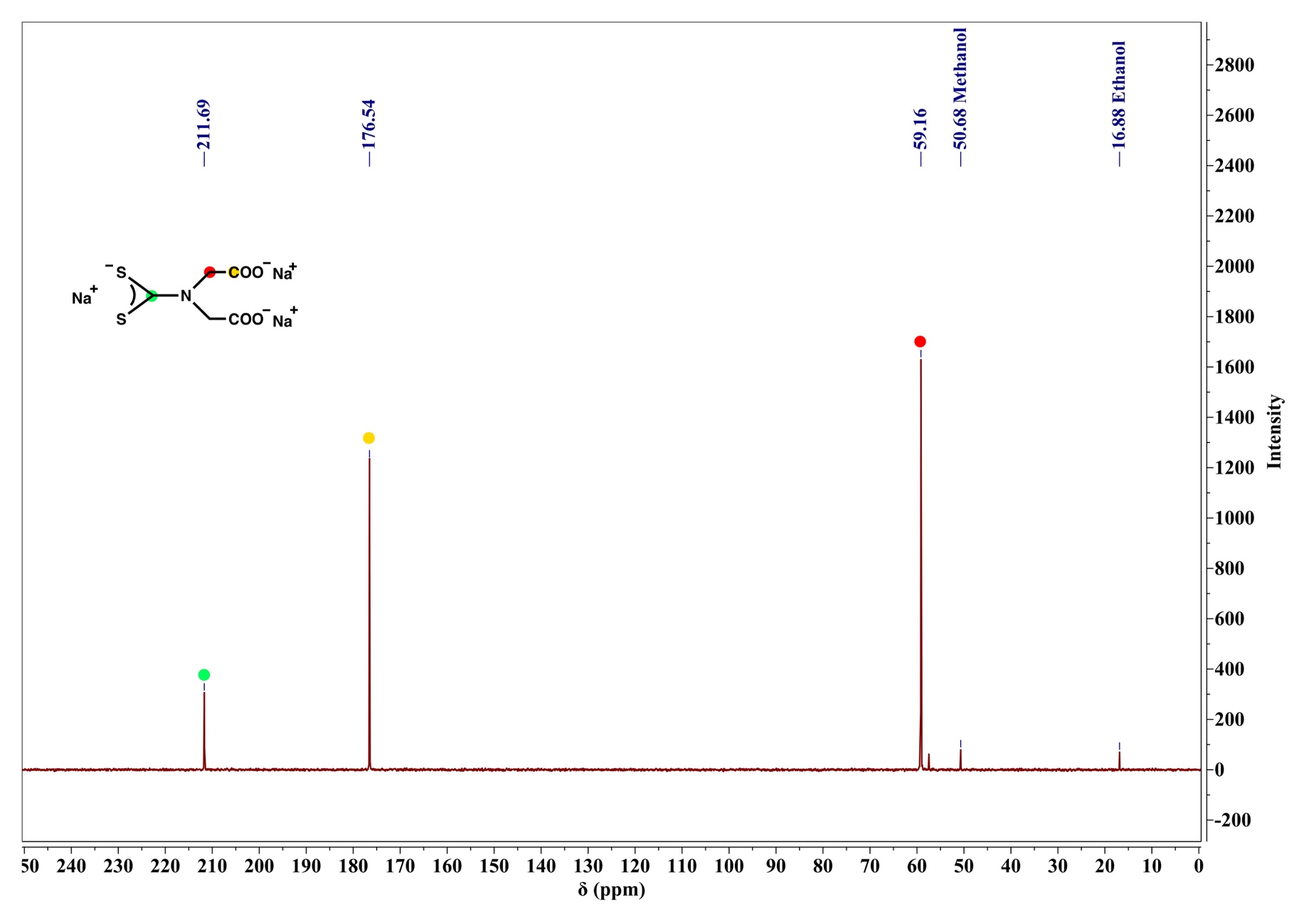
**

## **Figure S5.** ^13^C{^1^H} NMR (400 MHz) spectrum of Imd-Na-DTC in D_2_O

**
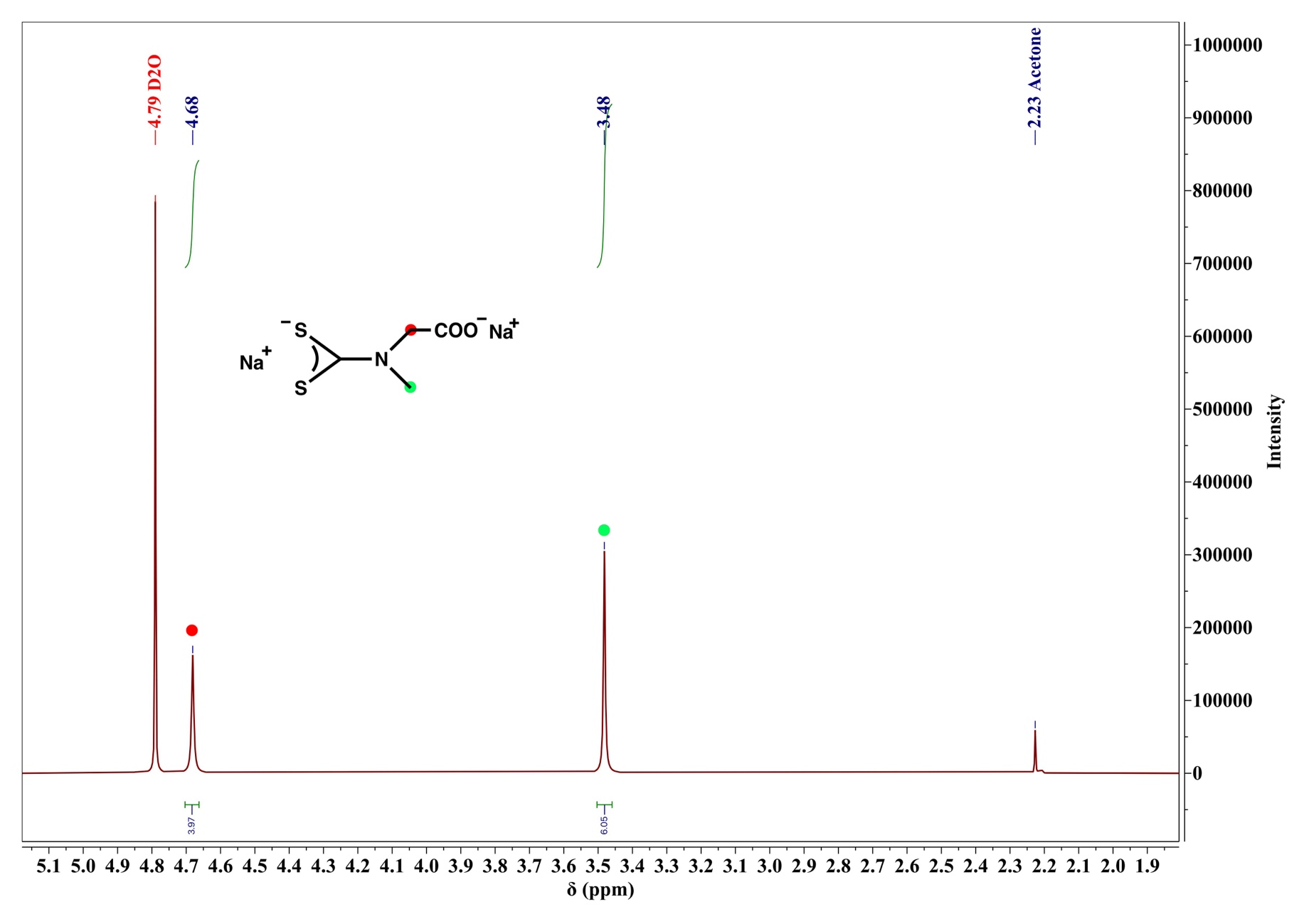
**

## **Figure S6.** ^1^H NMR (400 MHz) spectrum of Sar-Na-DTC in D_2_O


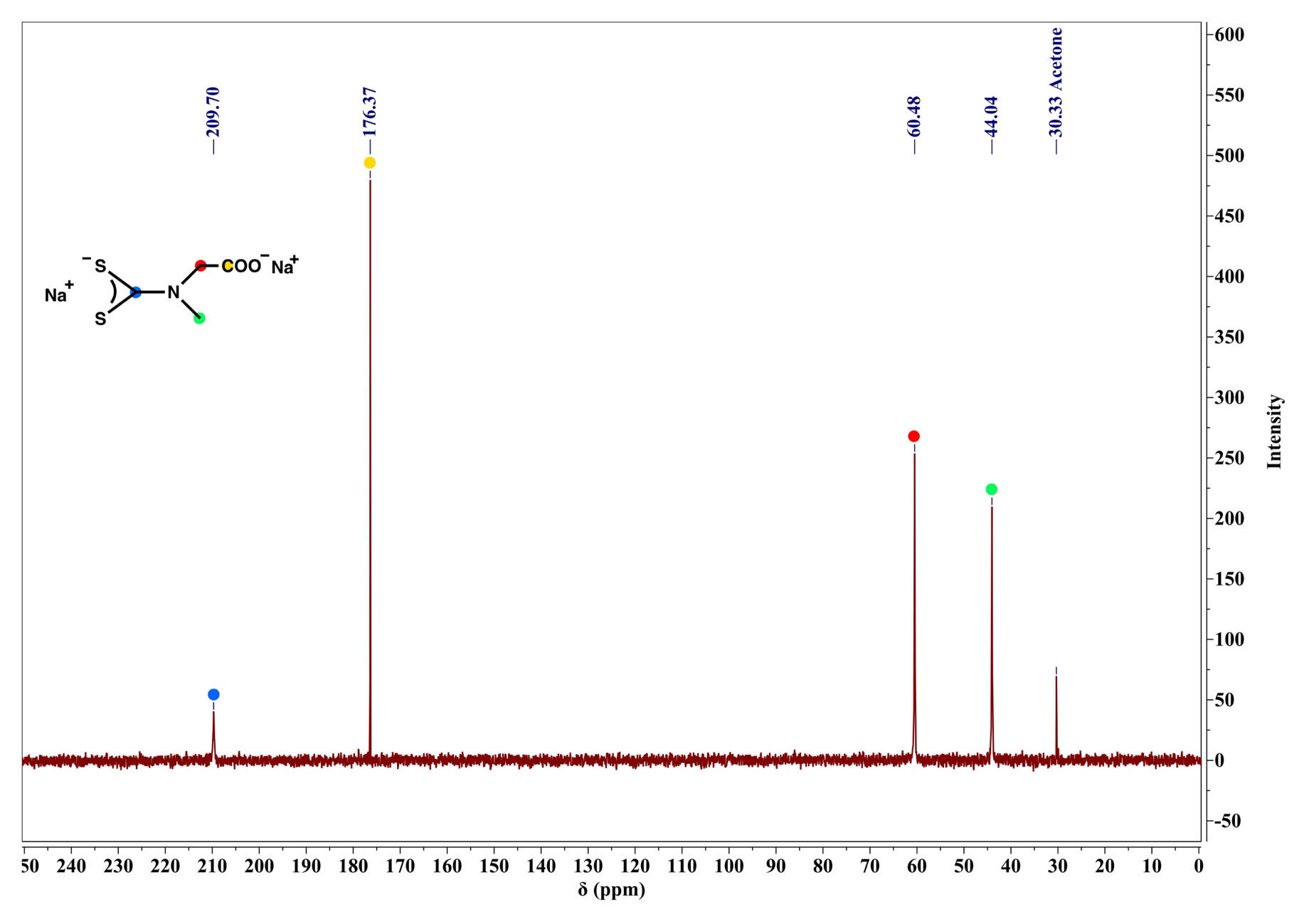


## **Figure S7.** ^13^C{^1^H} NMR (400 MHz) spectrum of Sar-Na-DTC in D_2_O


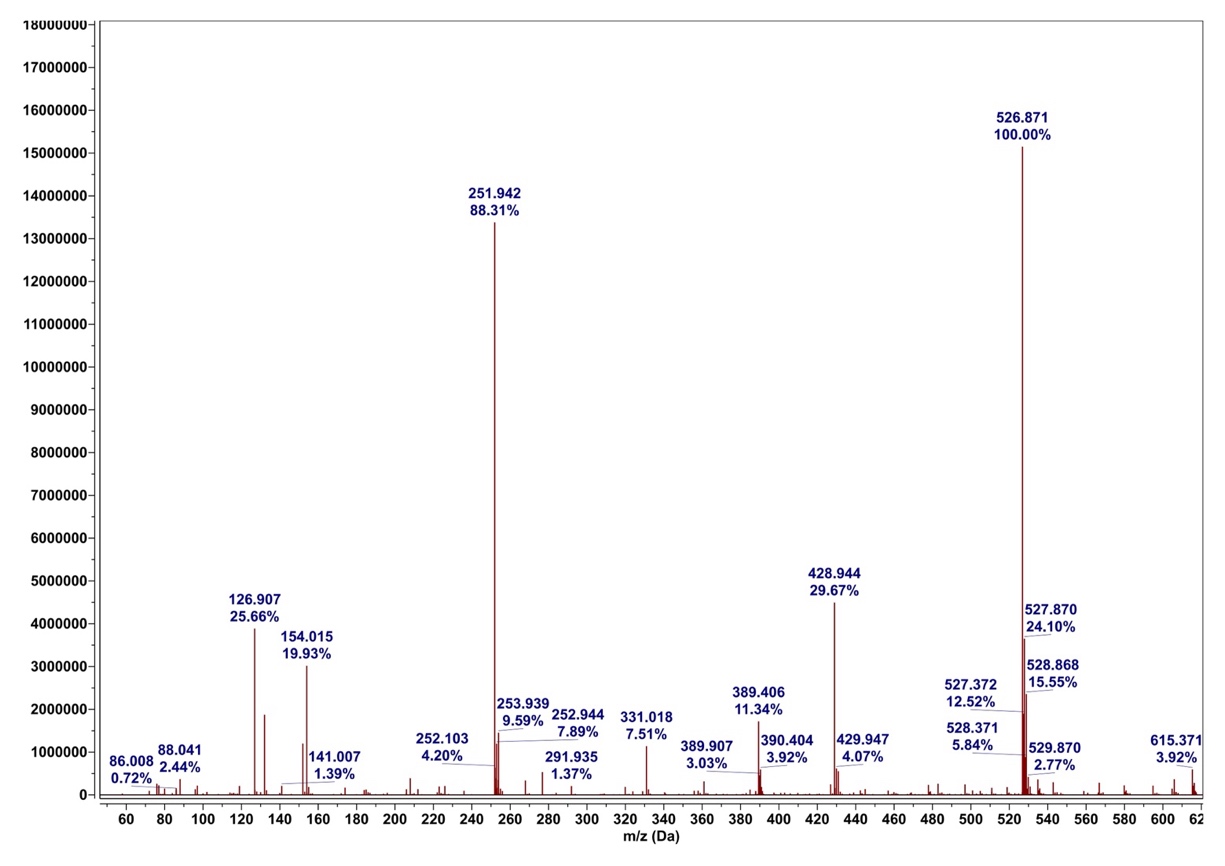


## **Figure S8.** ESI(-)-MS spectrum of Imd-Na-DTC in H_2_O. *m/z* = 251.942, [S_2_CN(CH_2_CO_2_)_2_Na_2_]^-^


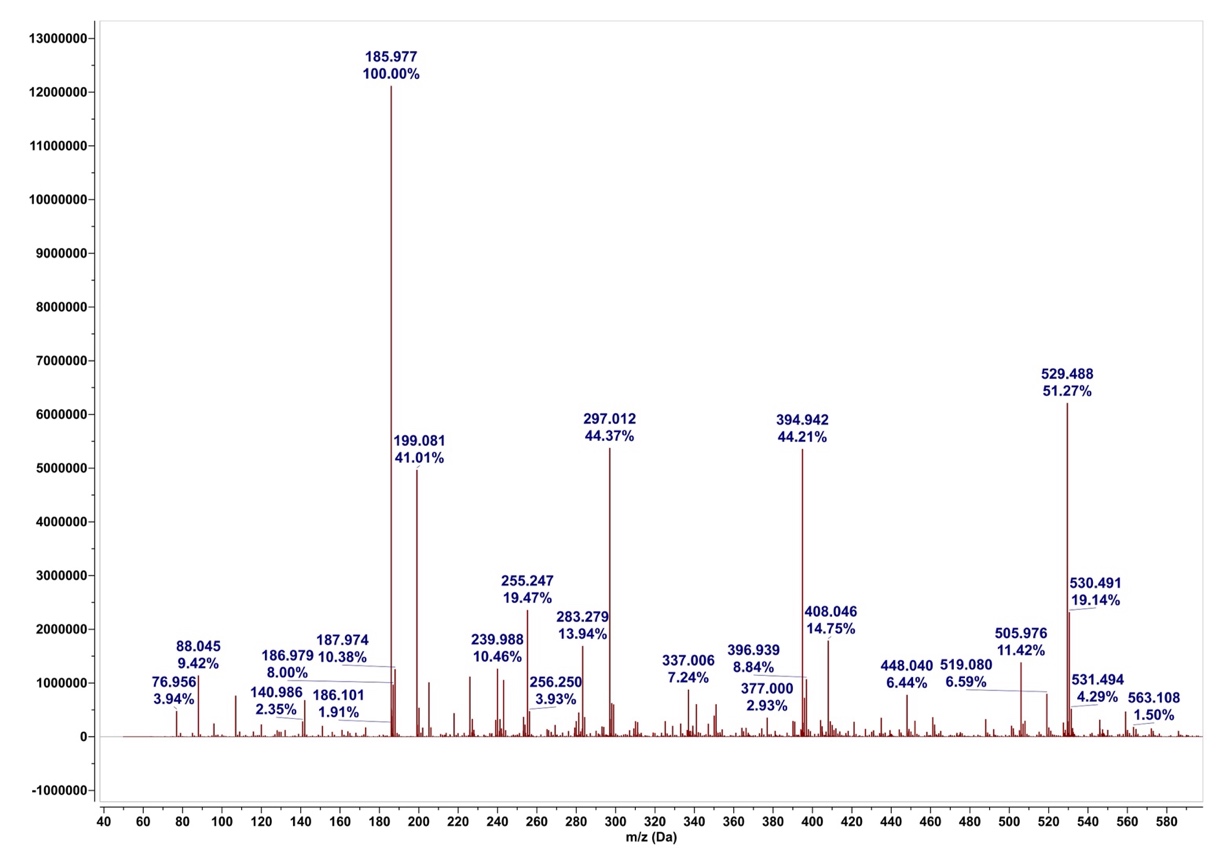


## **Figure S9.** ESI(-)-MS spectrum of Sar-Na-DTC in MeOH. *m/z =* 185.977, [S_2_CNMeCH_2_CO_2_Na]^-^


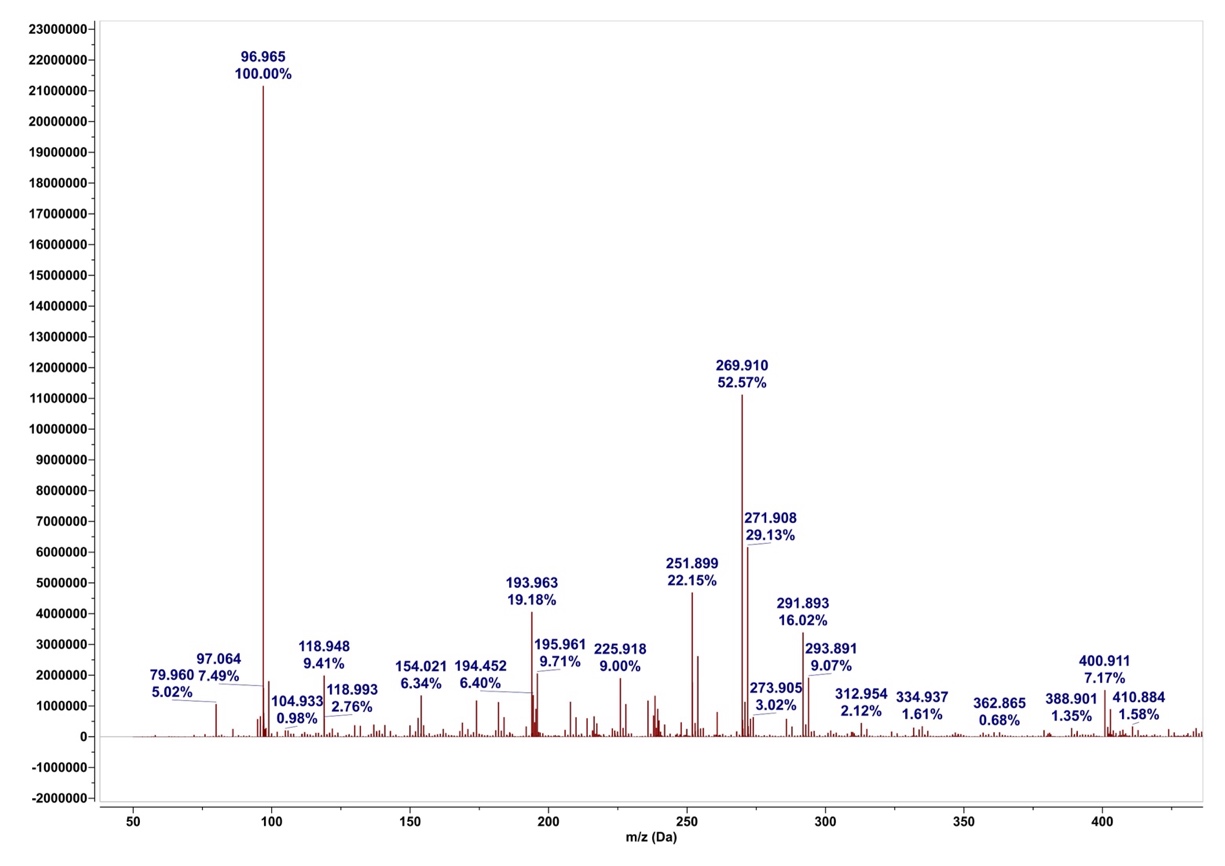


## **Figure S10.** ESI(-)-MS spectrum of **1-Na** in H_2_O


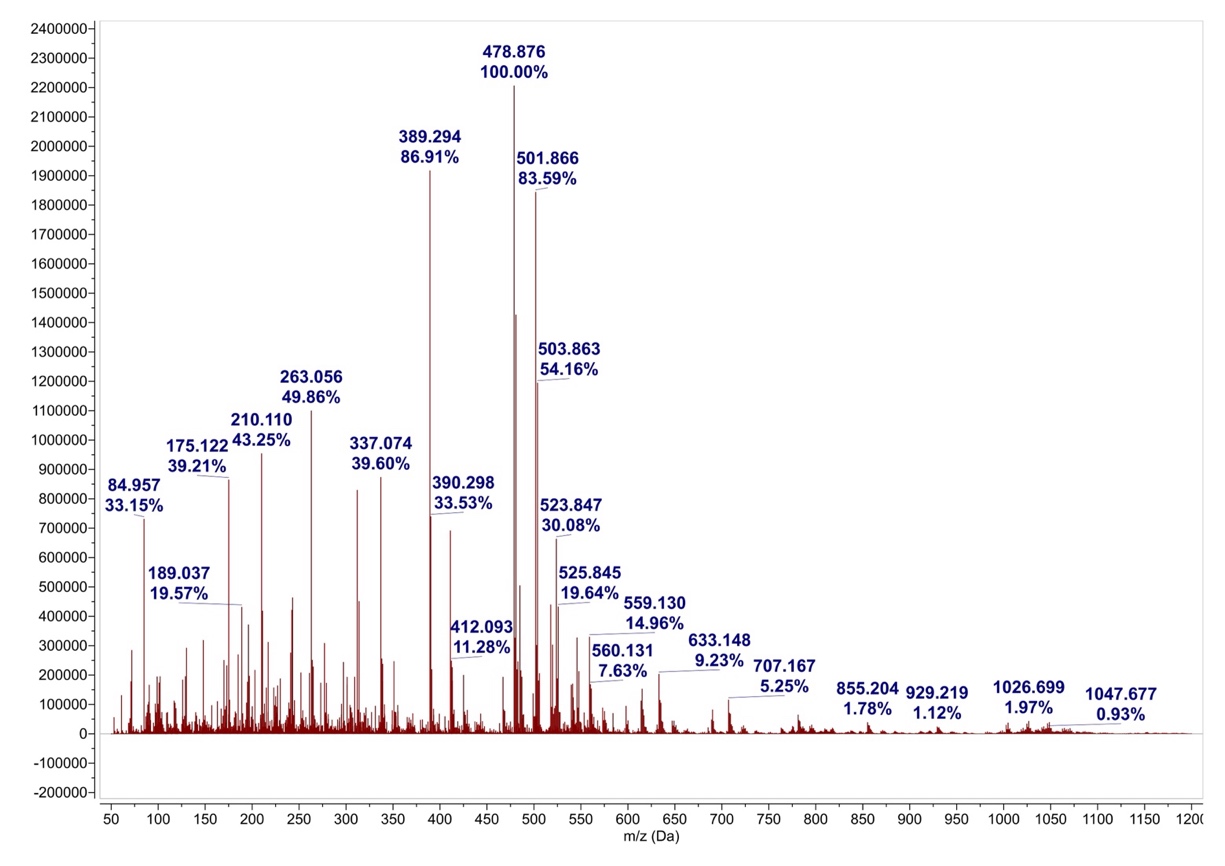


## **Figure S11.** ESI(+)-MS spectrum of **1-H** in MeOH


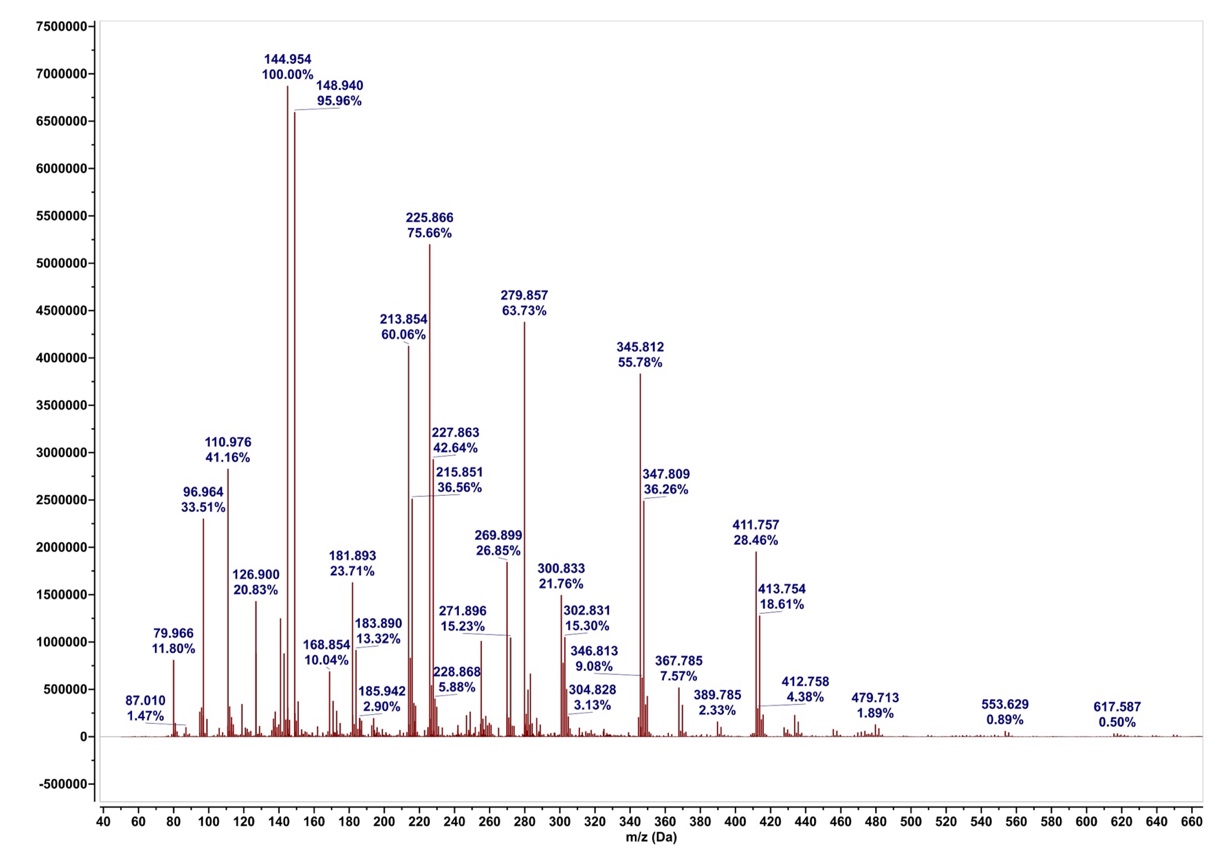


## **Figure S12.** ESI(-)-MS spectrum of **2-Na** in MeOH


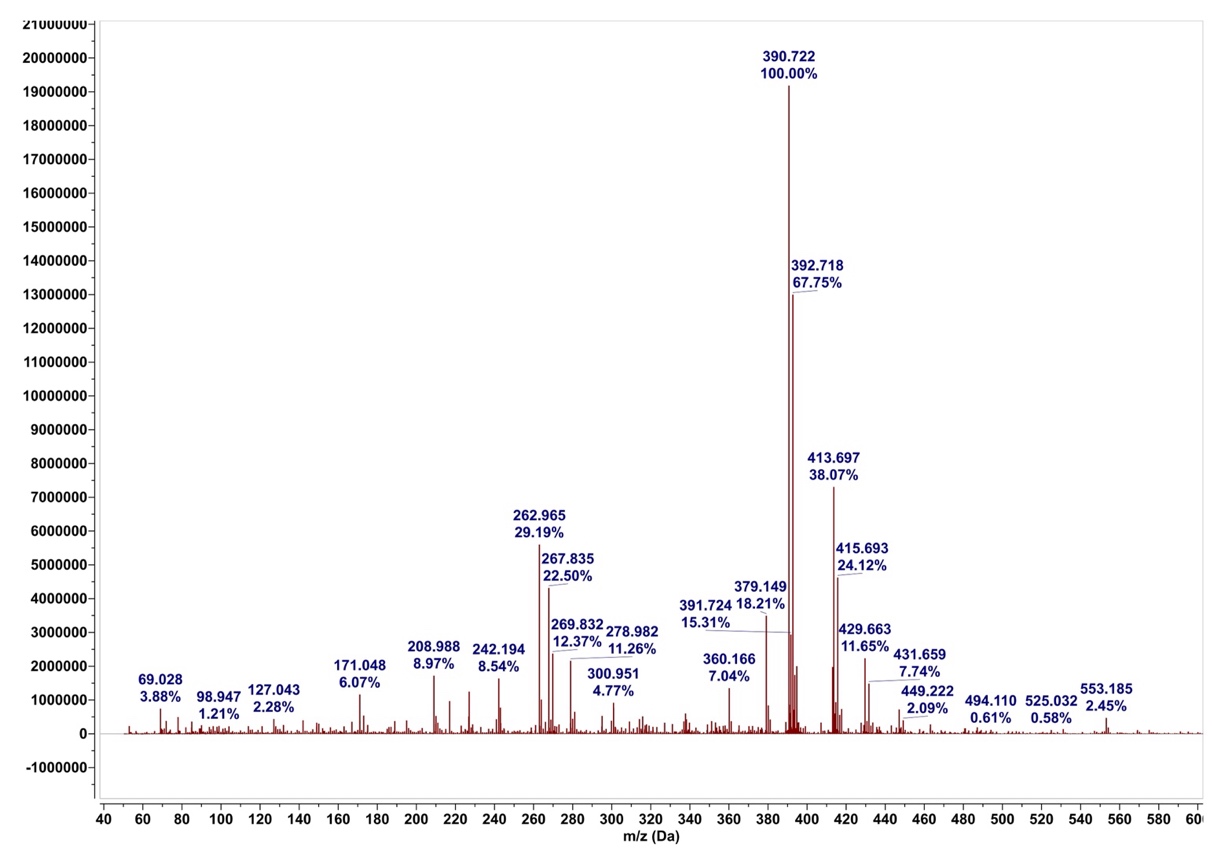


## **Figure S13.** ESI(+)-MS spectrum of **2-H** in MeOH


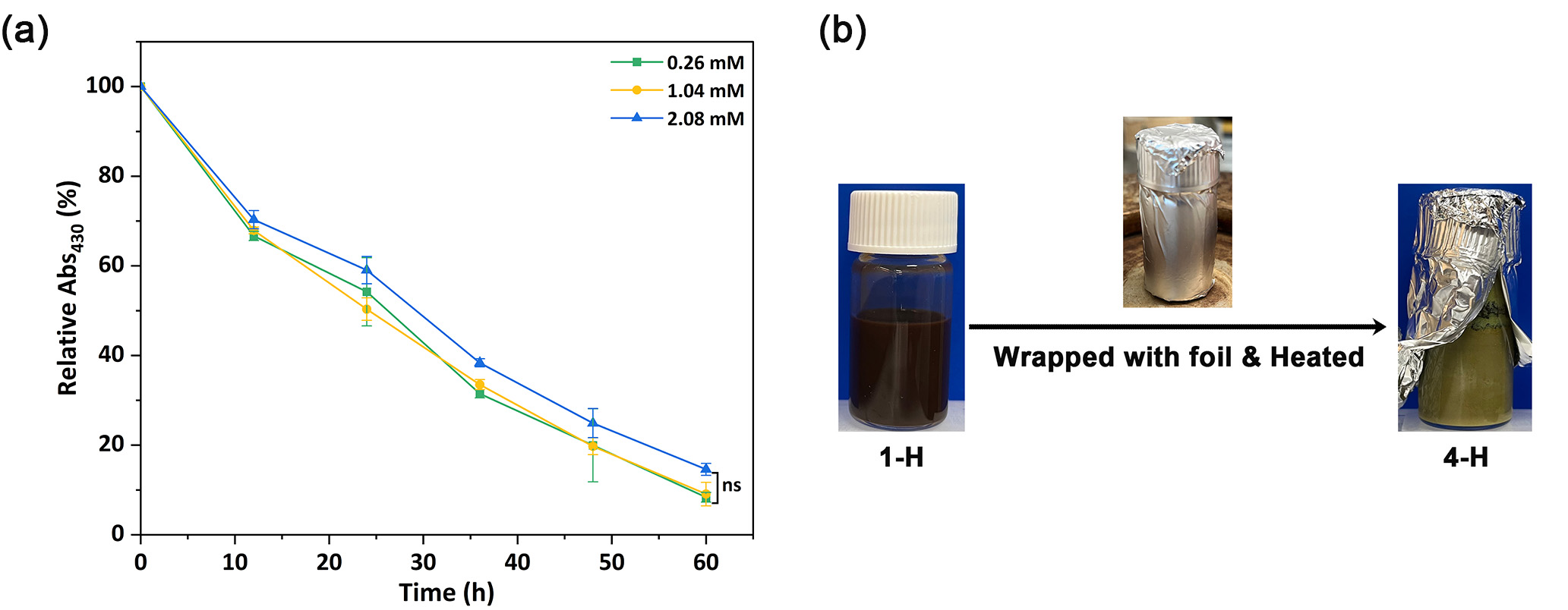


## **Figure S14**. (a) Plots showing the decrease of Abs_430_ as a function of time (days) at various concentrations of **1-H** (25 ℃), ns: not significantly different; (b) Images showing the reduction of **1-H** after being wrapped with foil


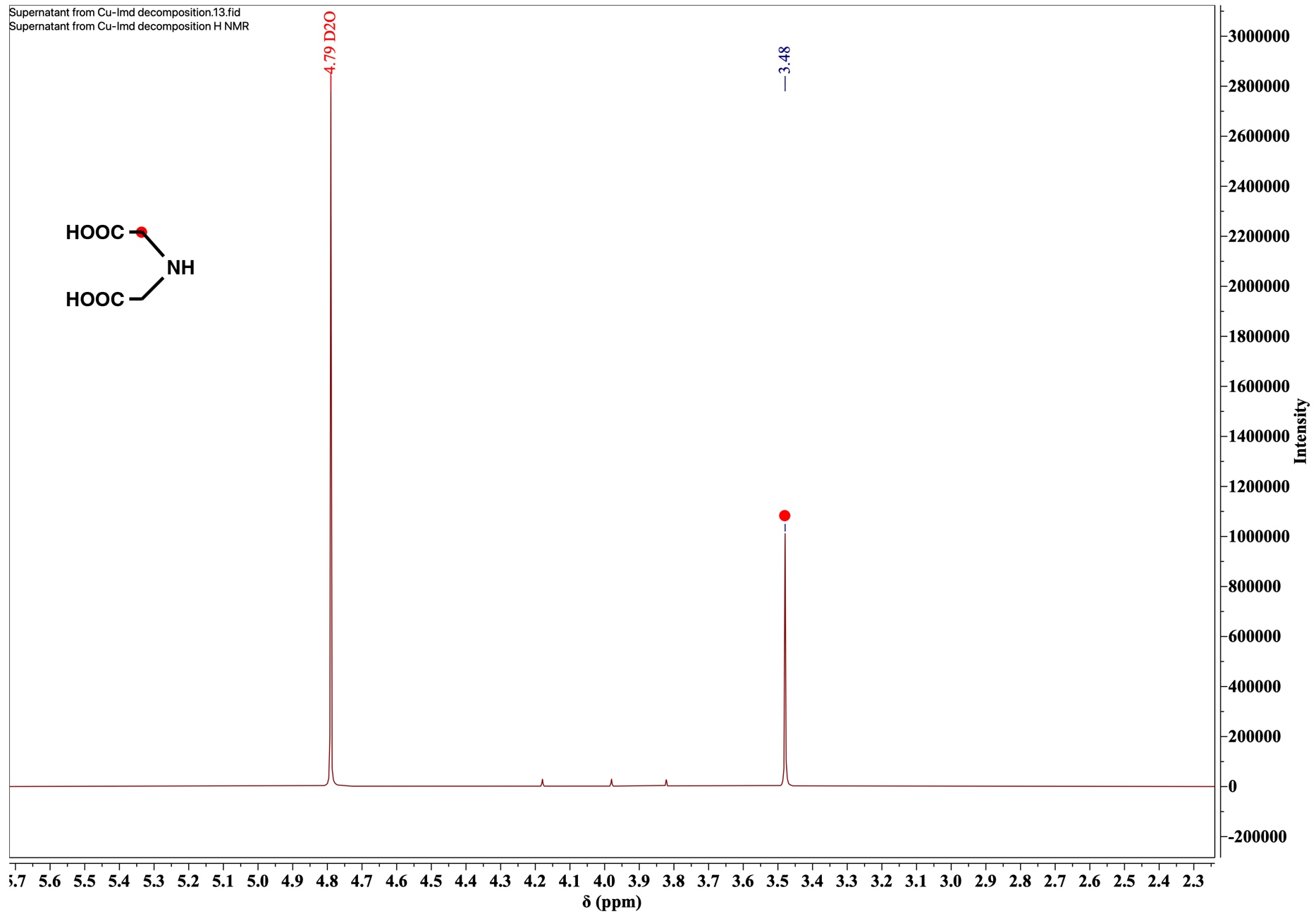


## **Figure S15.** ^1^H NMR (400 MHz) spectrum of the decomposition residue from **1-Na** in D_2_O


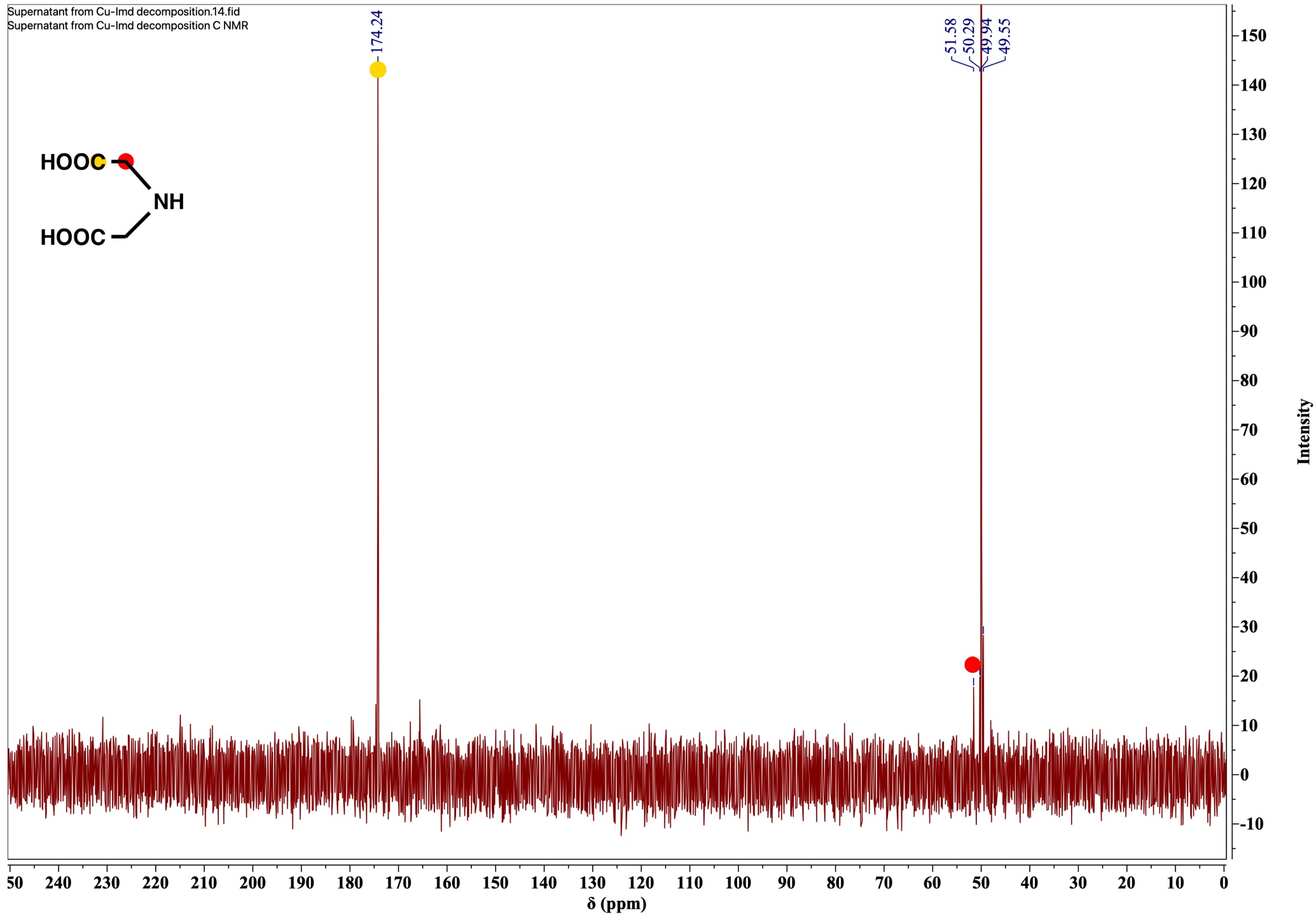


## **Figure S16.** ^13^C{^1^H} NMR (400 MHz) spectrum of the decomposition residue from **1-Na** in D_2_O


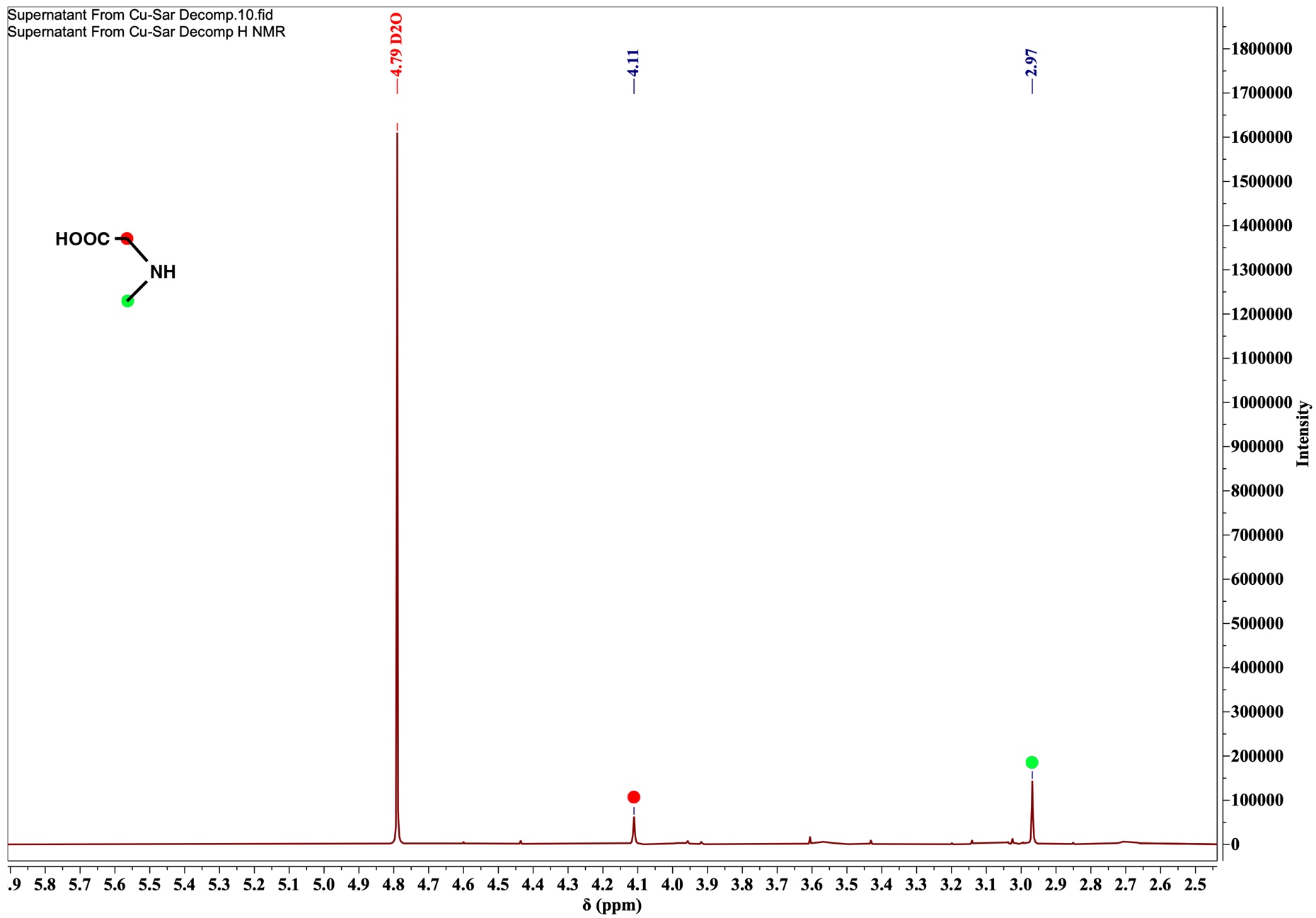


## **Figure S17.** ^1^H NMR (400 MHz) spectrum of the decomposition residue from **2-Na** in D_2_O


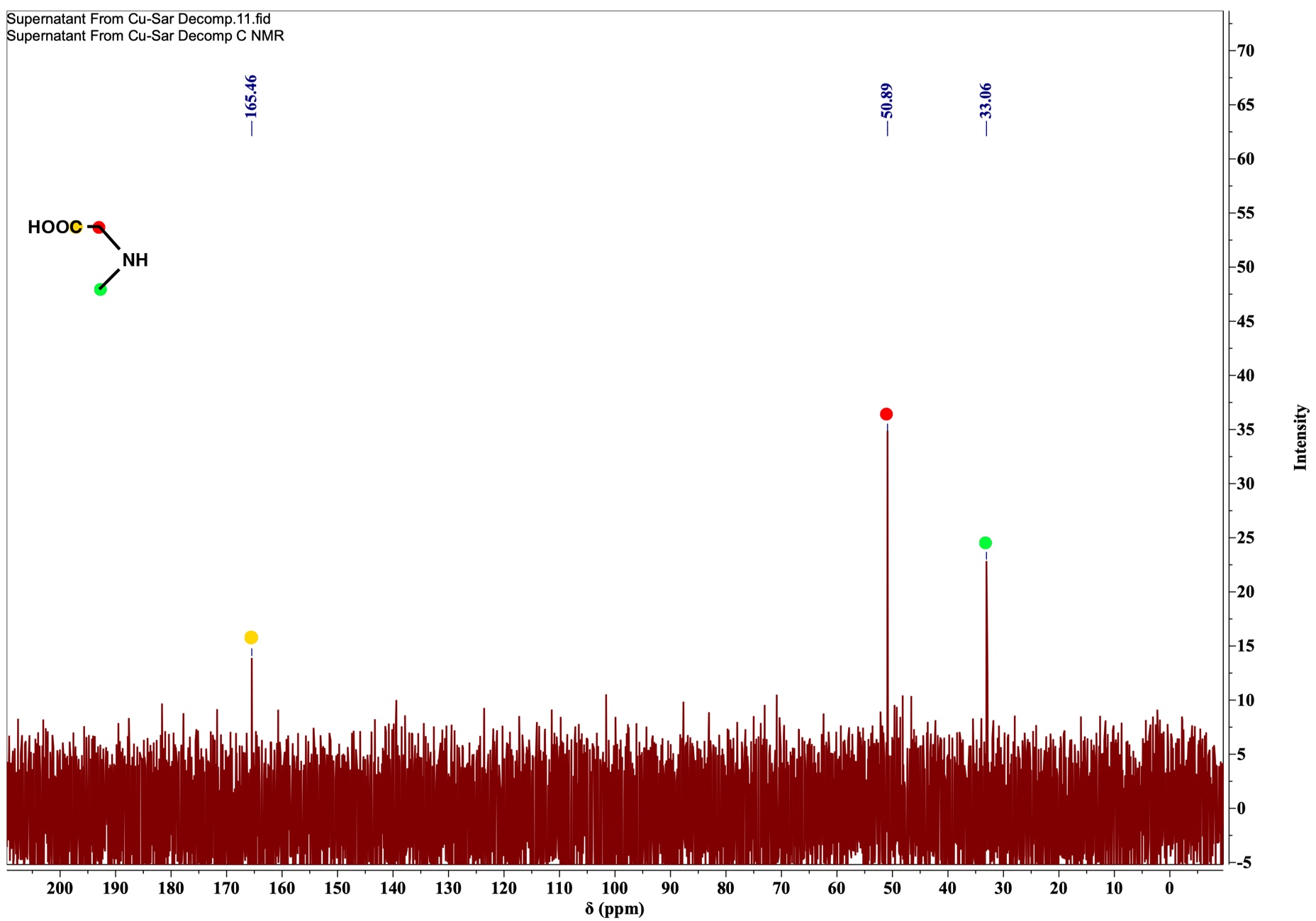


## **Figure S18.** ^13^C{^1^H} NMR (400 MHz) spectrum of the decomposition residue from **2-Na** in D_2_O

**
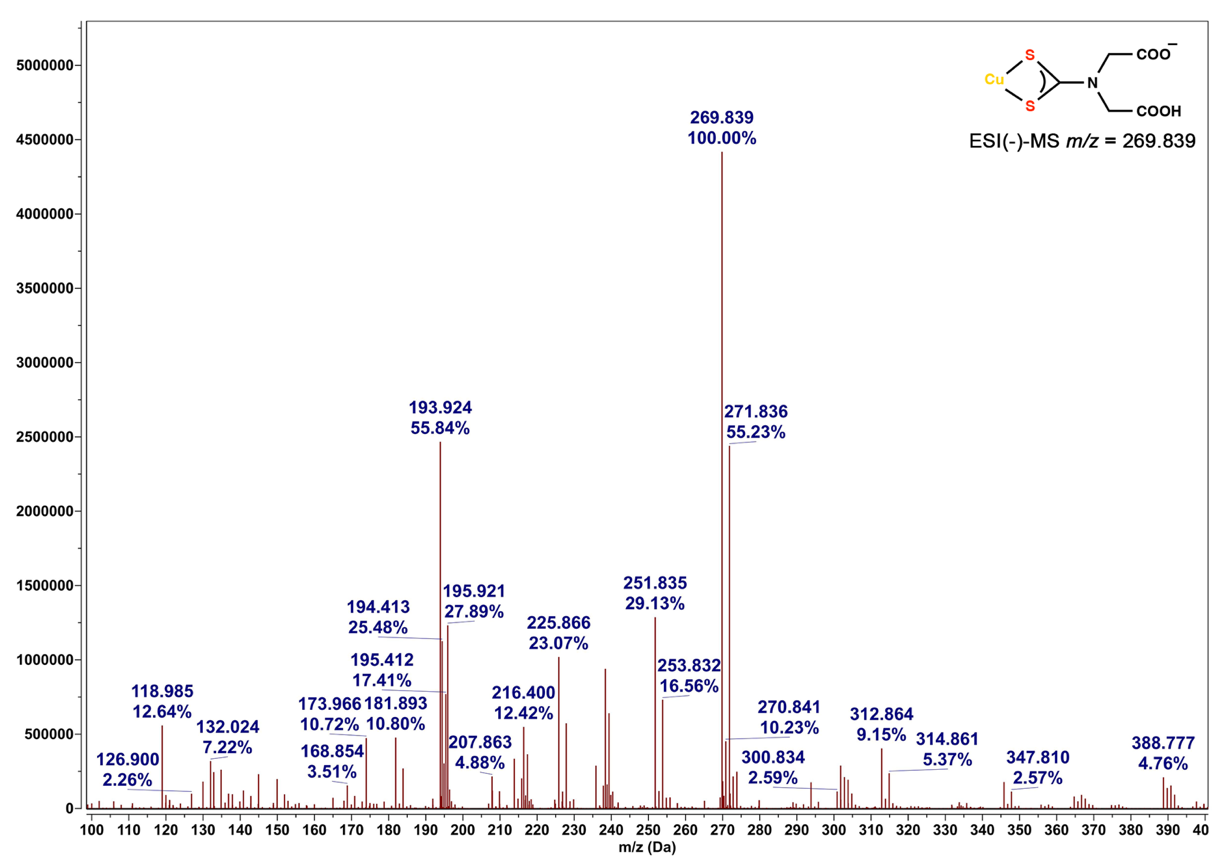
**

## **Figure S19.** ESI(-)-MS spectrum of the isolated 4 in CH_3_CN

**
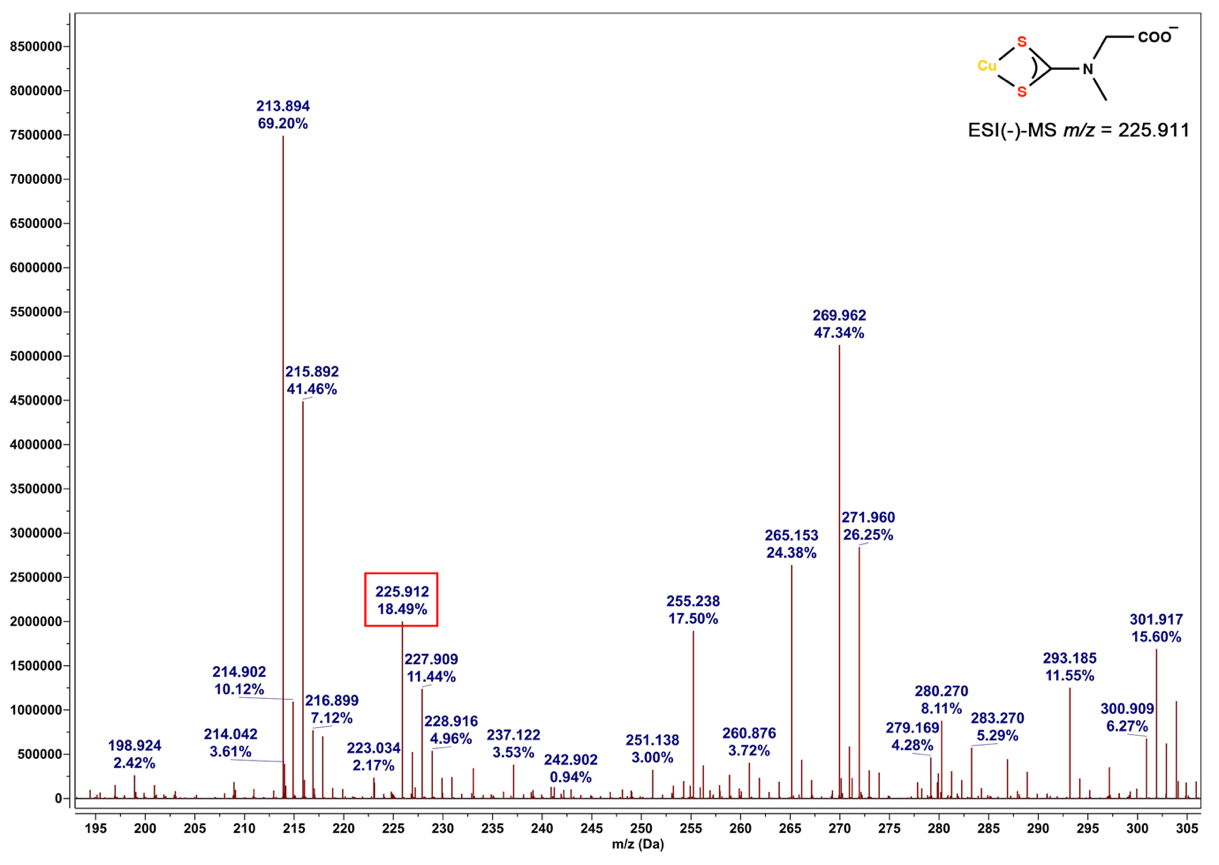
**

## **Figure S20.** ESI(-)-MS spectrum of the isolated 5 in MeOH

**
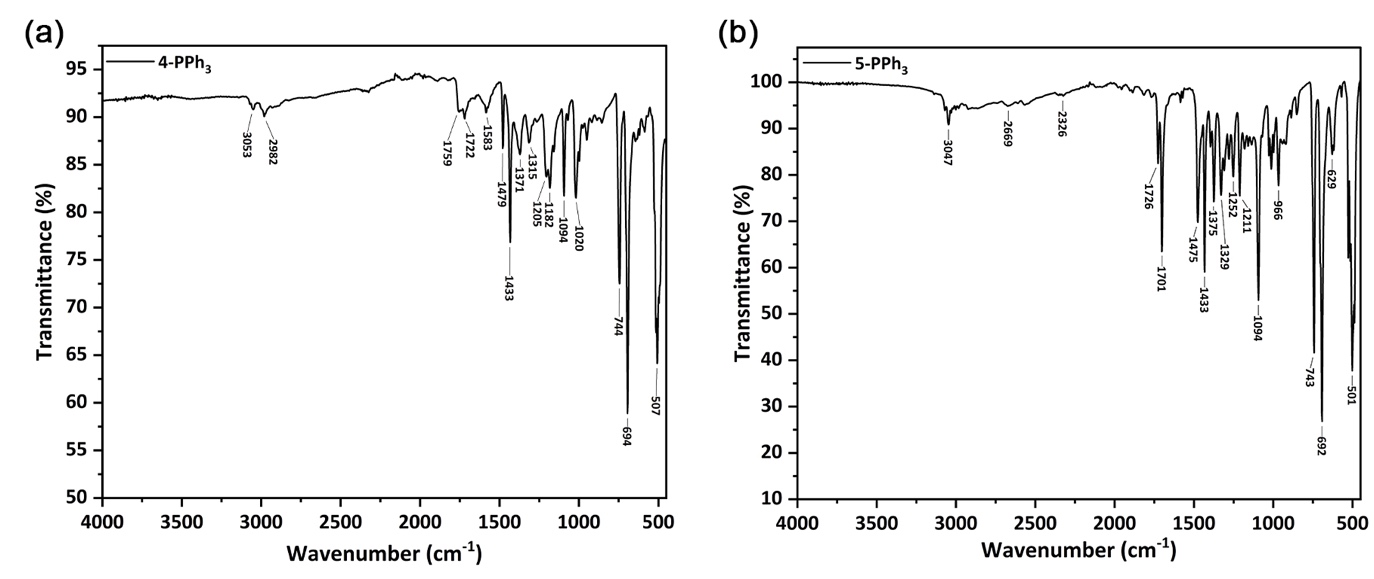
**

## **Figure S21.** IR spectra of synthesized (a) 4-PPh_3_ and (b) 5-PPh_3_


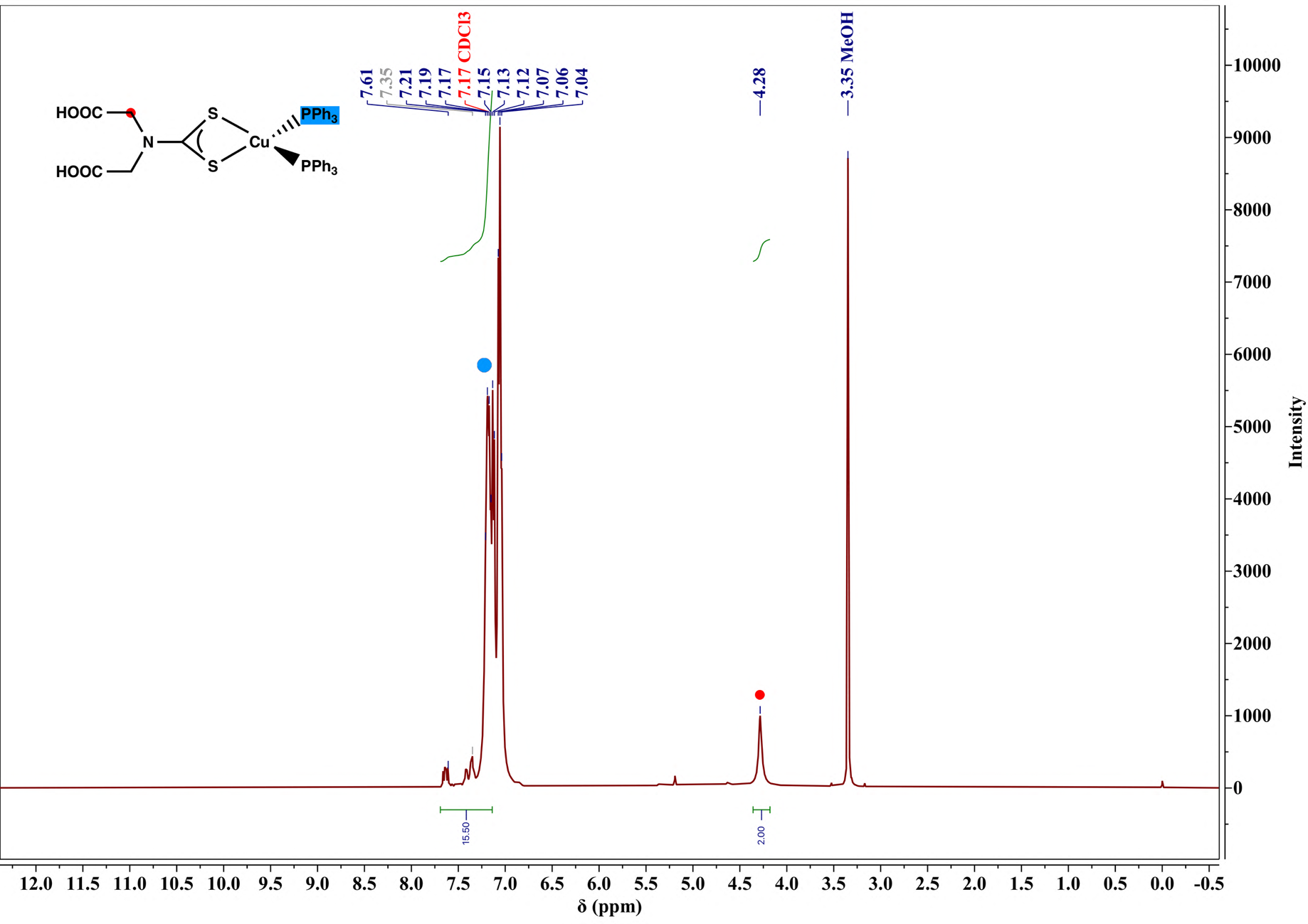


## **Figure S22.** ^1^H NMR (400 MHz) spectrum of **4-PPh_3_** in CDCl_3_


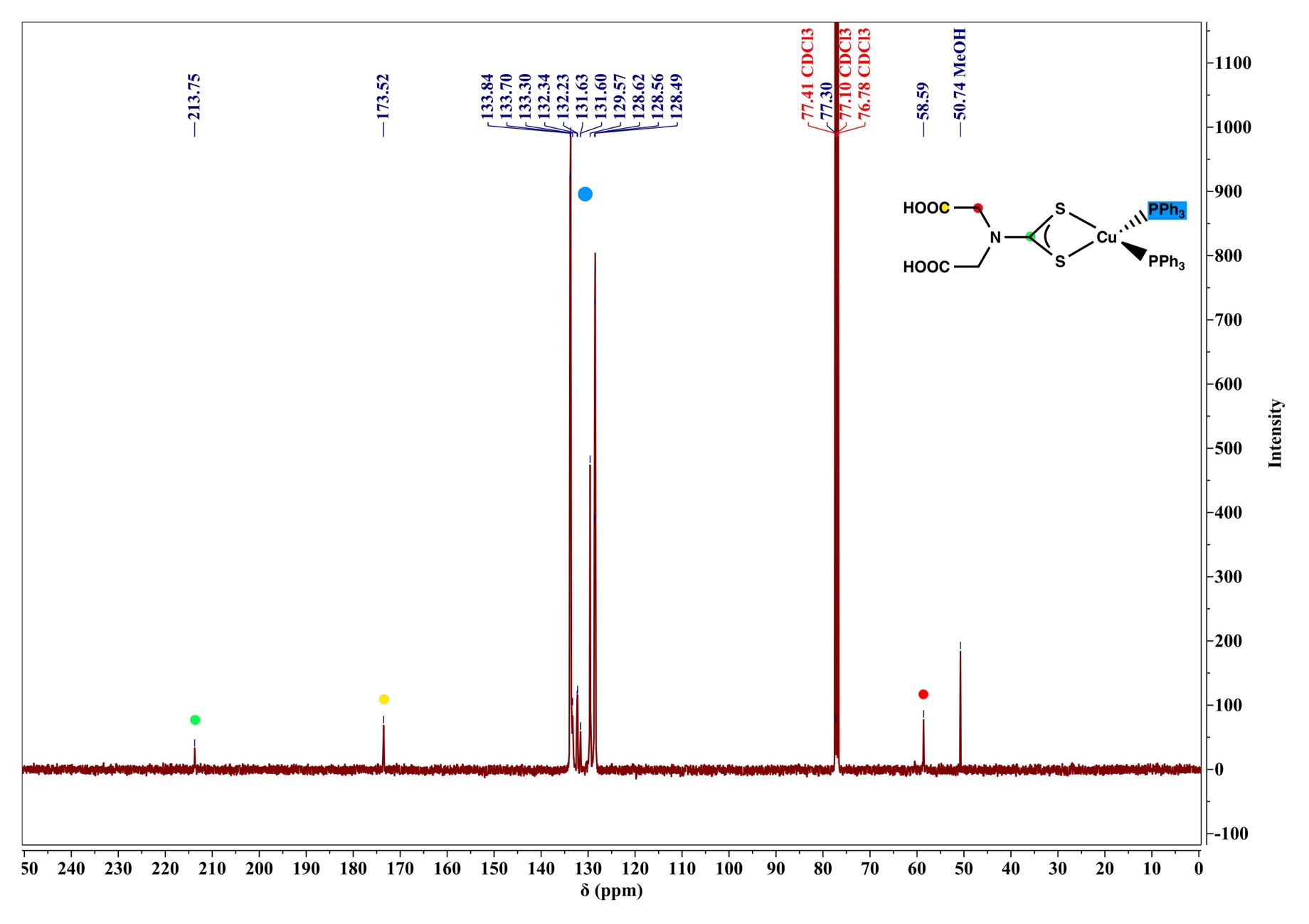


## **Figure S23.** ^13^C{^1^H} NMR (400 MHz) spectrum of **4-PPh_3_** in CDCl_3_


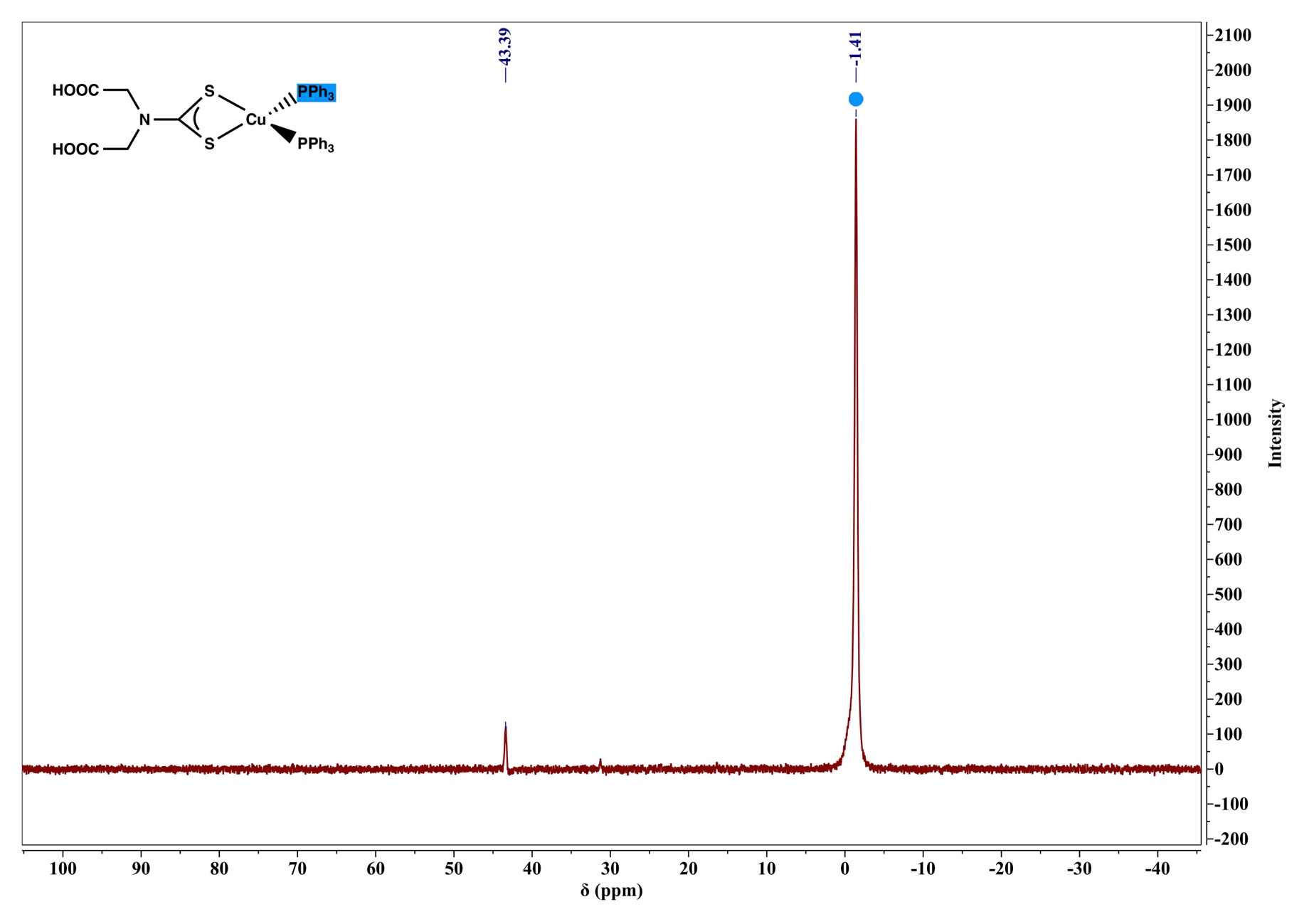


## **Figure S24.** ^31^P{^1^H} NMR (400 MHz) spectrum of **4-PPh_3_** in CDCl_3_


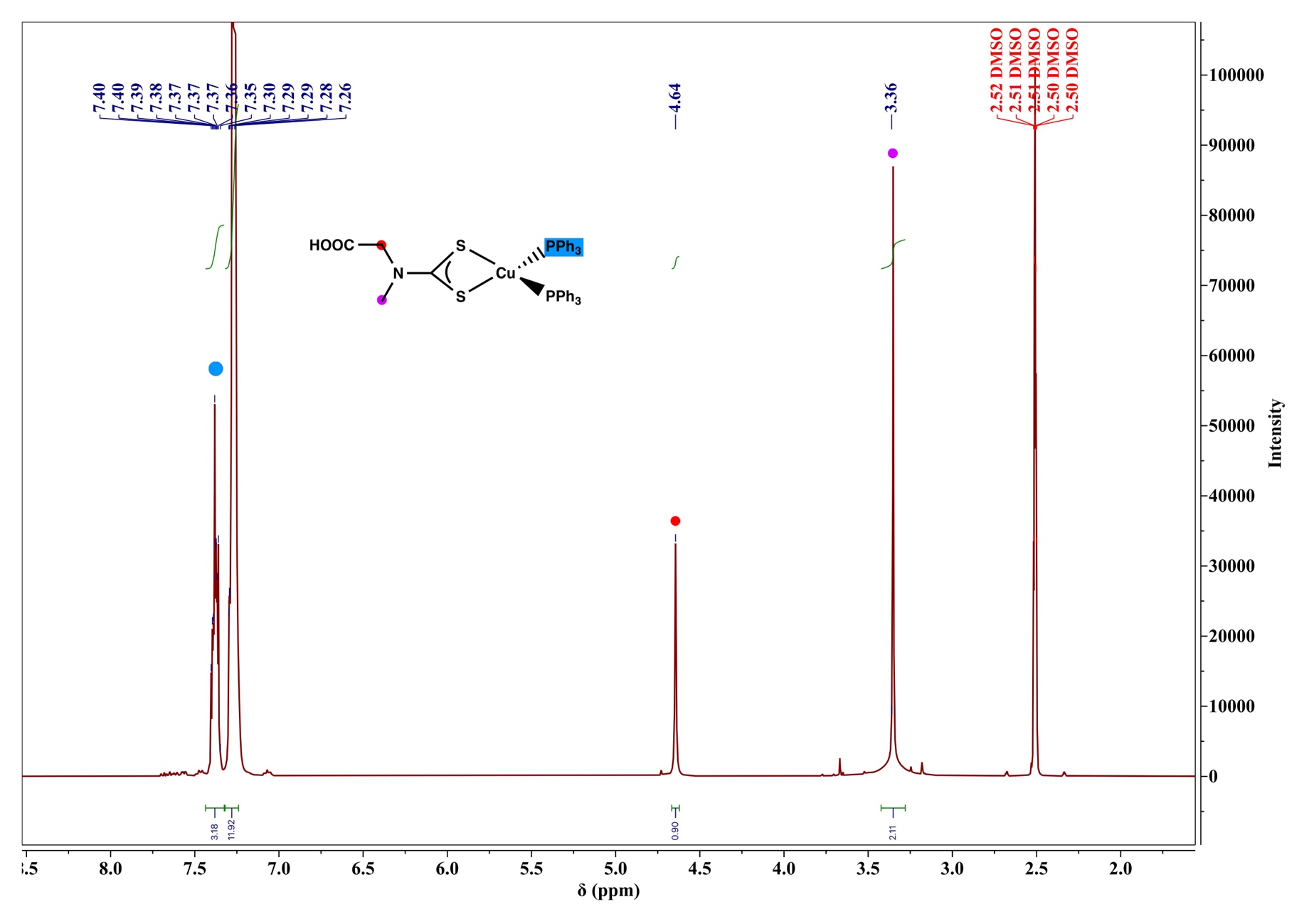


## **Figure S25.** ^1^H NMR (400 MHz) spectrum of **5-PPh_3_** in DMSO-*d_6_*


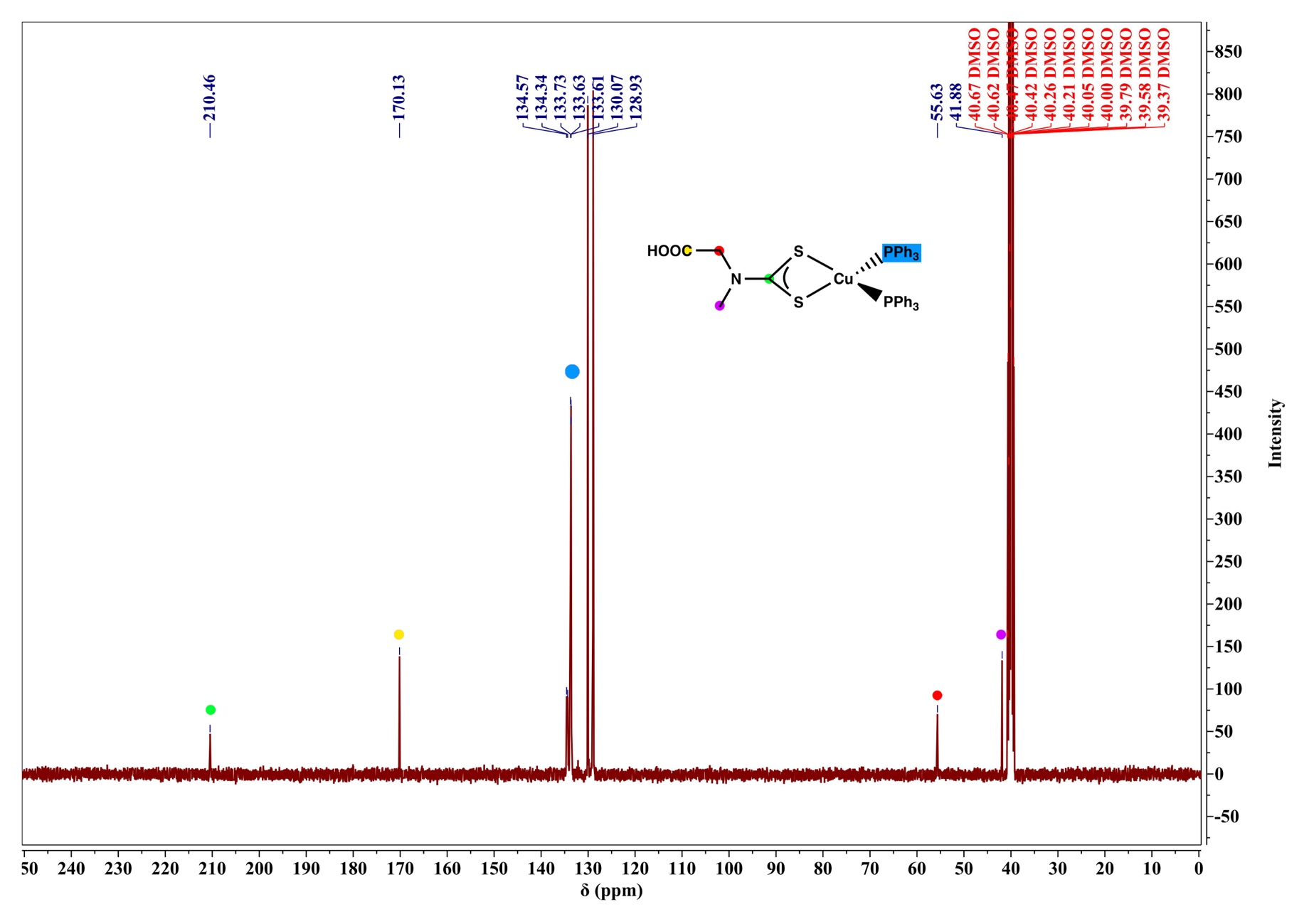


## **Figure S26.** ^13^C{^1^H} NMR (400 MHz) spectrum of **5-PPh_3_** in DMSO-*d_6_*


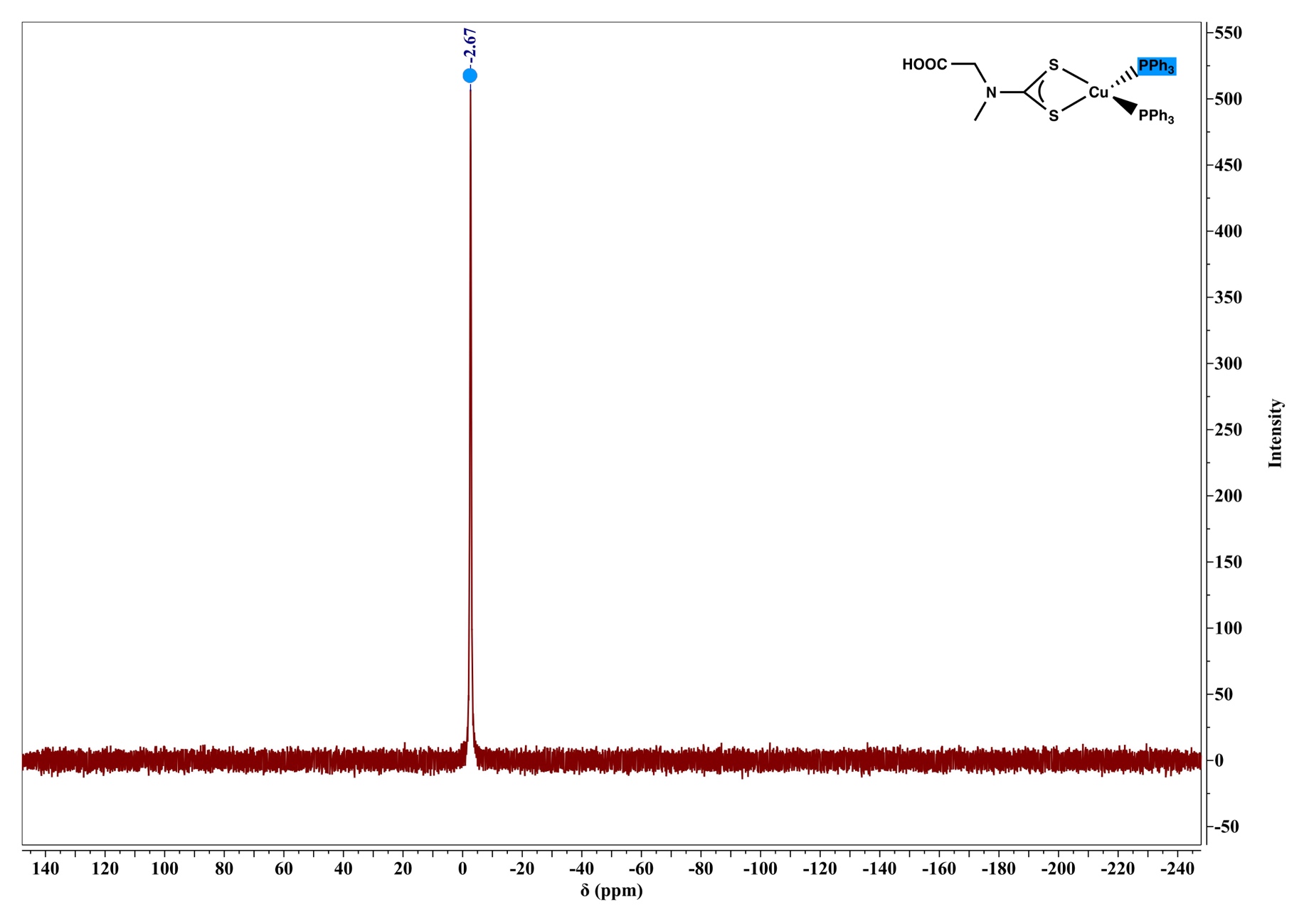


## **Figure S27.** ^31^P{^1^H} NMR (400 MHz) spectrum of **5-PPh_3_** in DMSO-*d_6_*


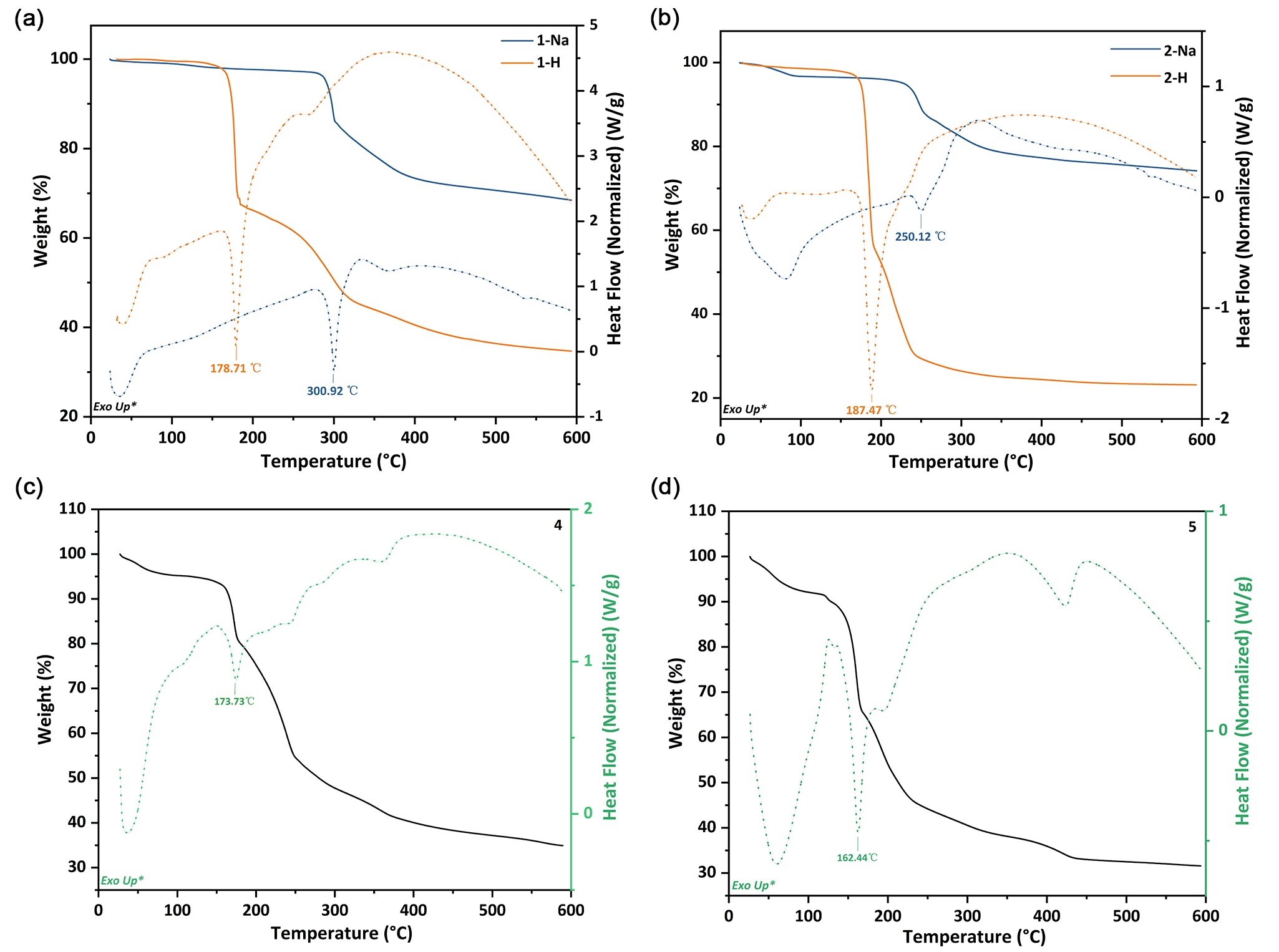


## **Figure S28.** TGA (solid lines) and DSC (dotted lines) curves of (a) **1-H** and **1-Na**, (b) **2-H** and **2-Na**, (c) **4**, and (d) **5**


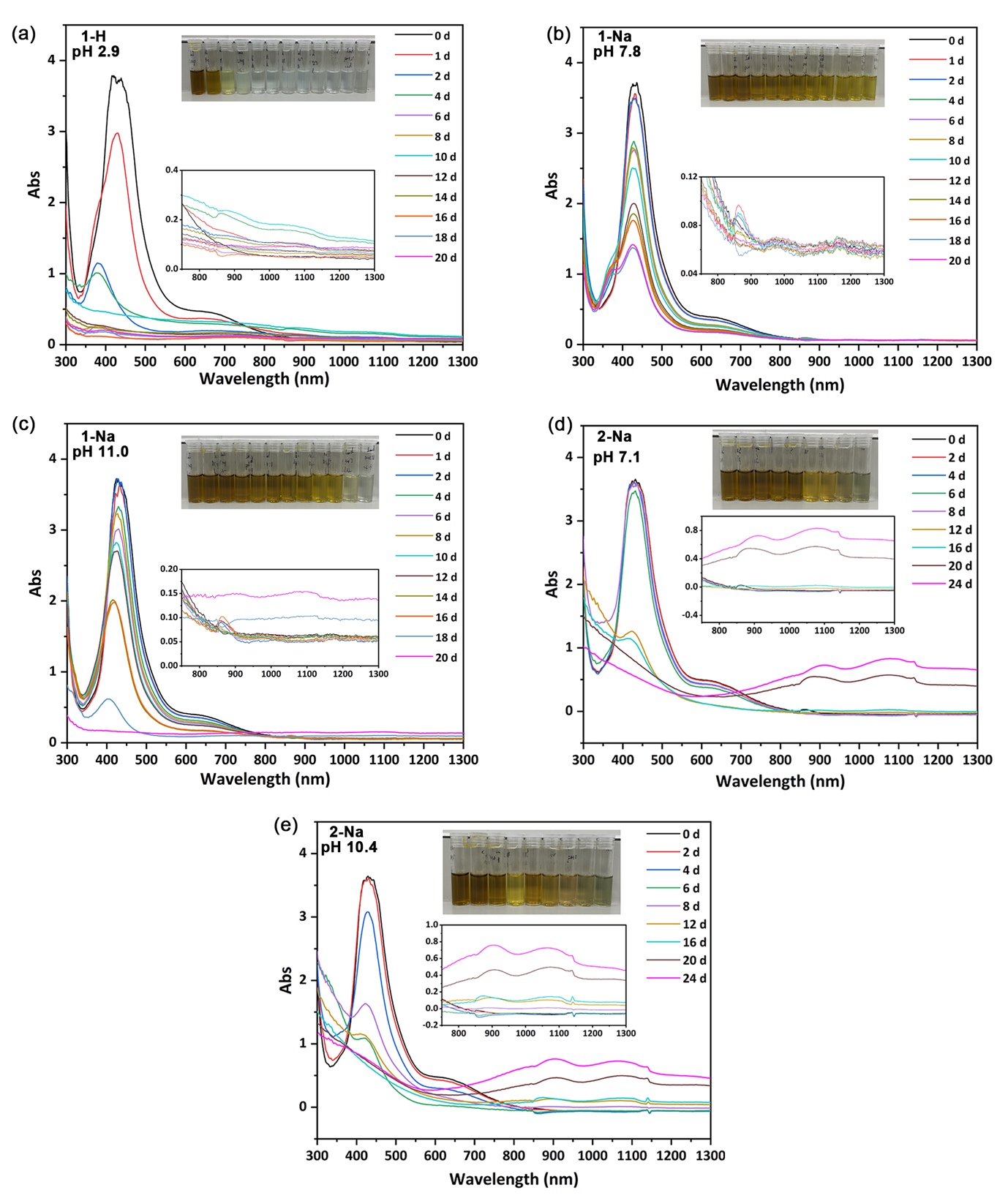


## **Figure S29.** Room temperature decomposition of Cu(II)-DTCs in H_2_O: UV-Vis-NIR spectra showing the absorbance change against time and inserted images show the color change, (a) **1-H** (pH 2.9); (b) **1-Na** (pH 7.8); (c) **1-Na** (pH 11.0); (d) **2-Na** (pH 7.1); (e) **2-Na** (pH 10.4).


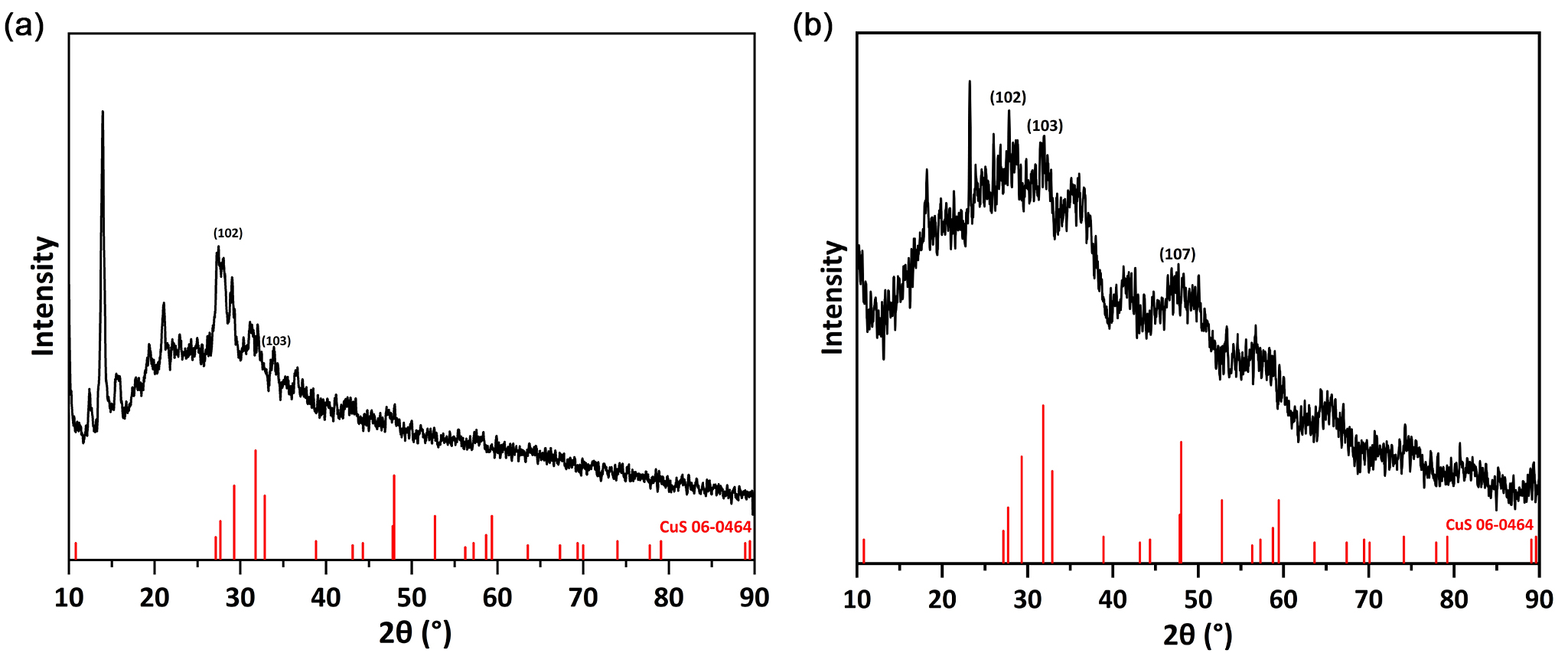


## **Figure S30**. PXRD patterns of (a) **4** and (b) **5** isolated from the heating method. CuS (Covellite, JCPDS No. 06-0464) is used as a reference


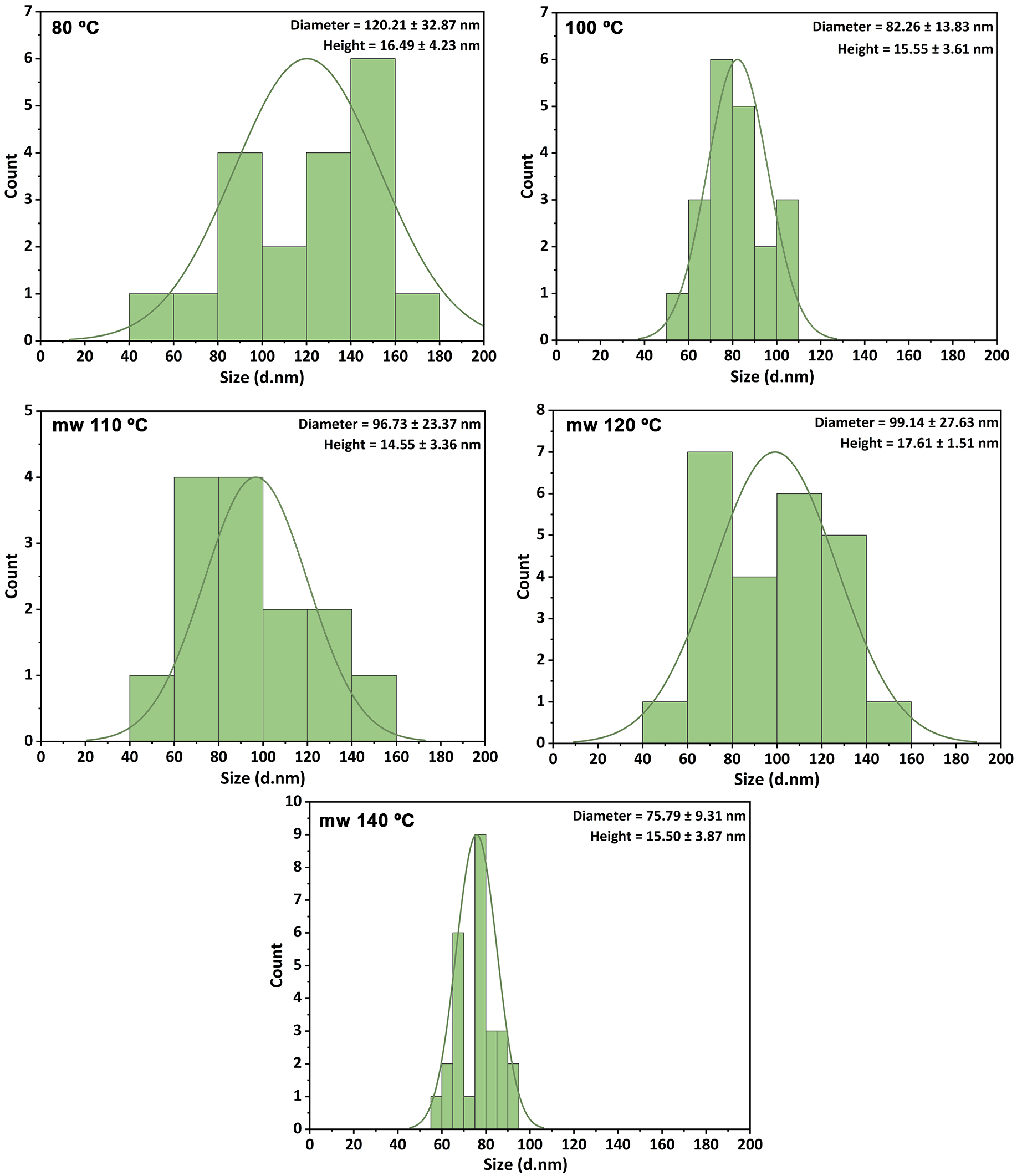


## **Figure S31.** Size distribution of NPs obtained from **1-Na** (pH 7.8) at different temperatures. NPs from 90 ℃ are presented in **Figure S31 (1-Na** pH 7.8**)**. Microwave-assisted decompositions were abbreviated as “mw”


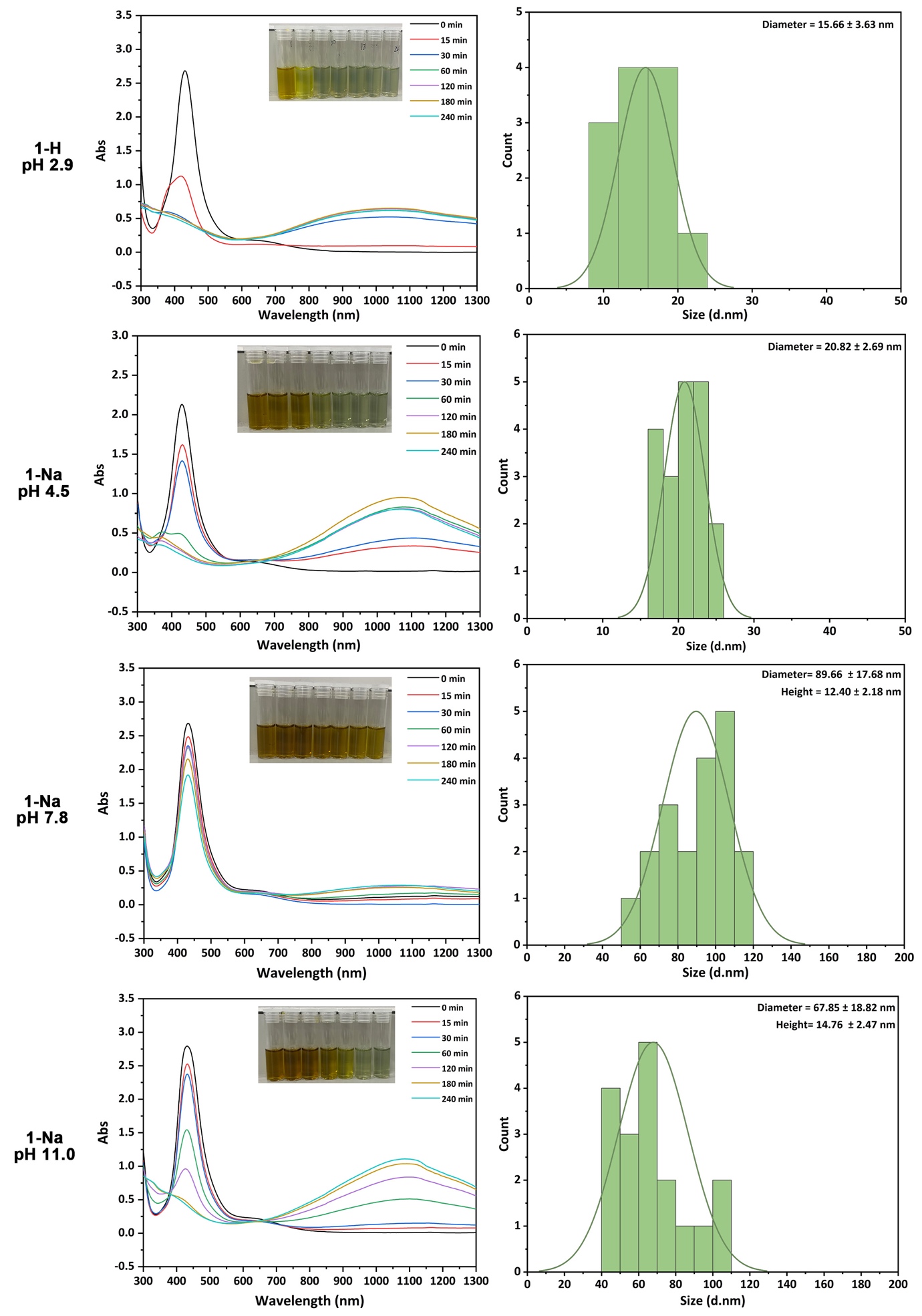


## **Figure S32.** Decomposition studies of **1** at different pH: UV-Vis-NIR spectra and size distribution (90 ℃)


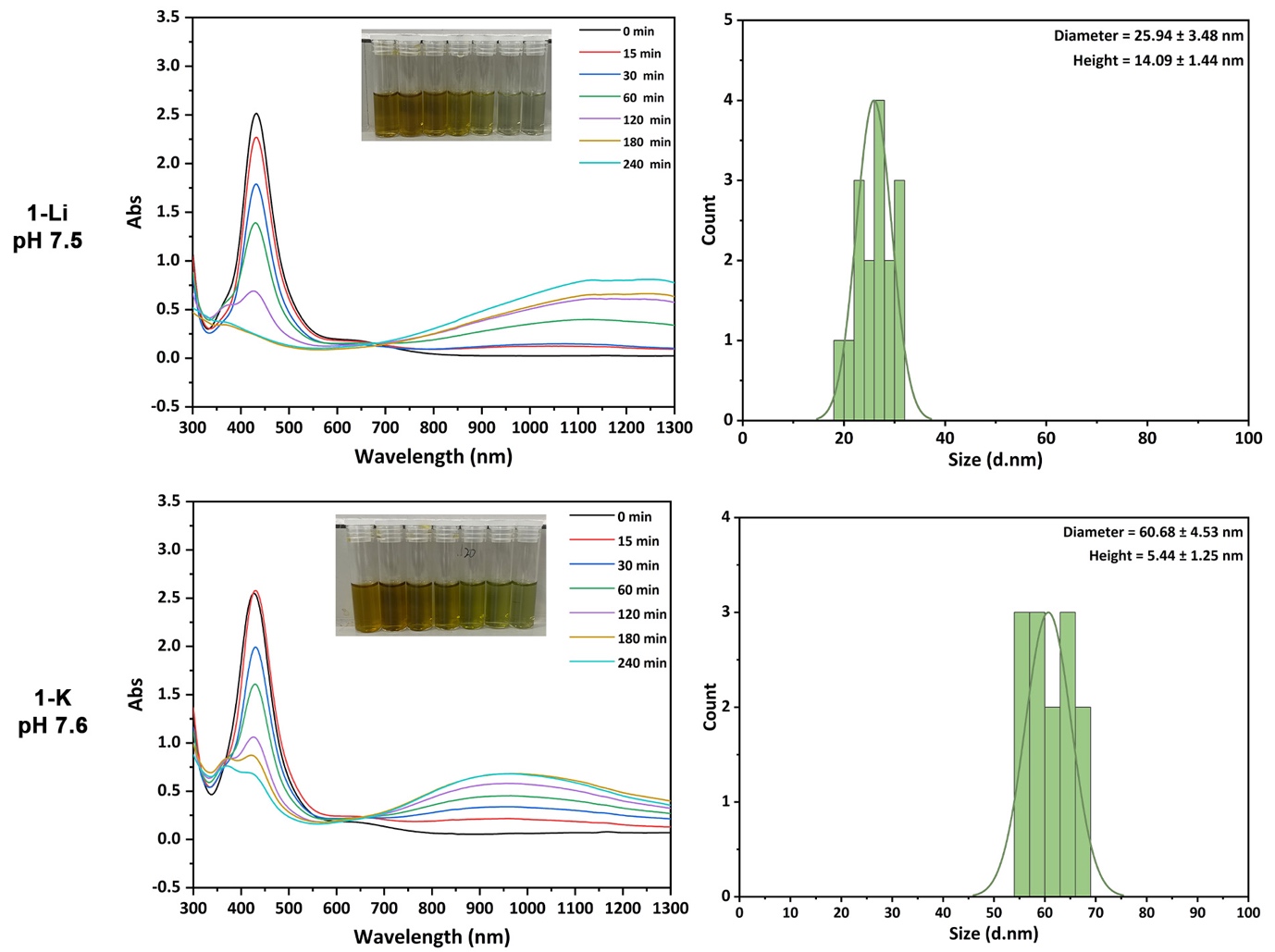


## **Figure S33.** Decomposition studies of **1** with different cations: UV-Vis-NIR spectra and size distribution (90 ℃)


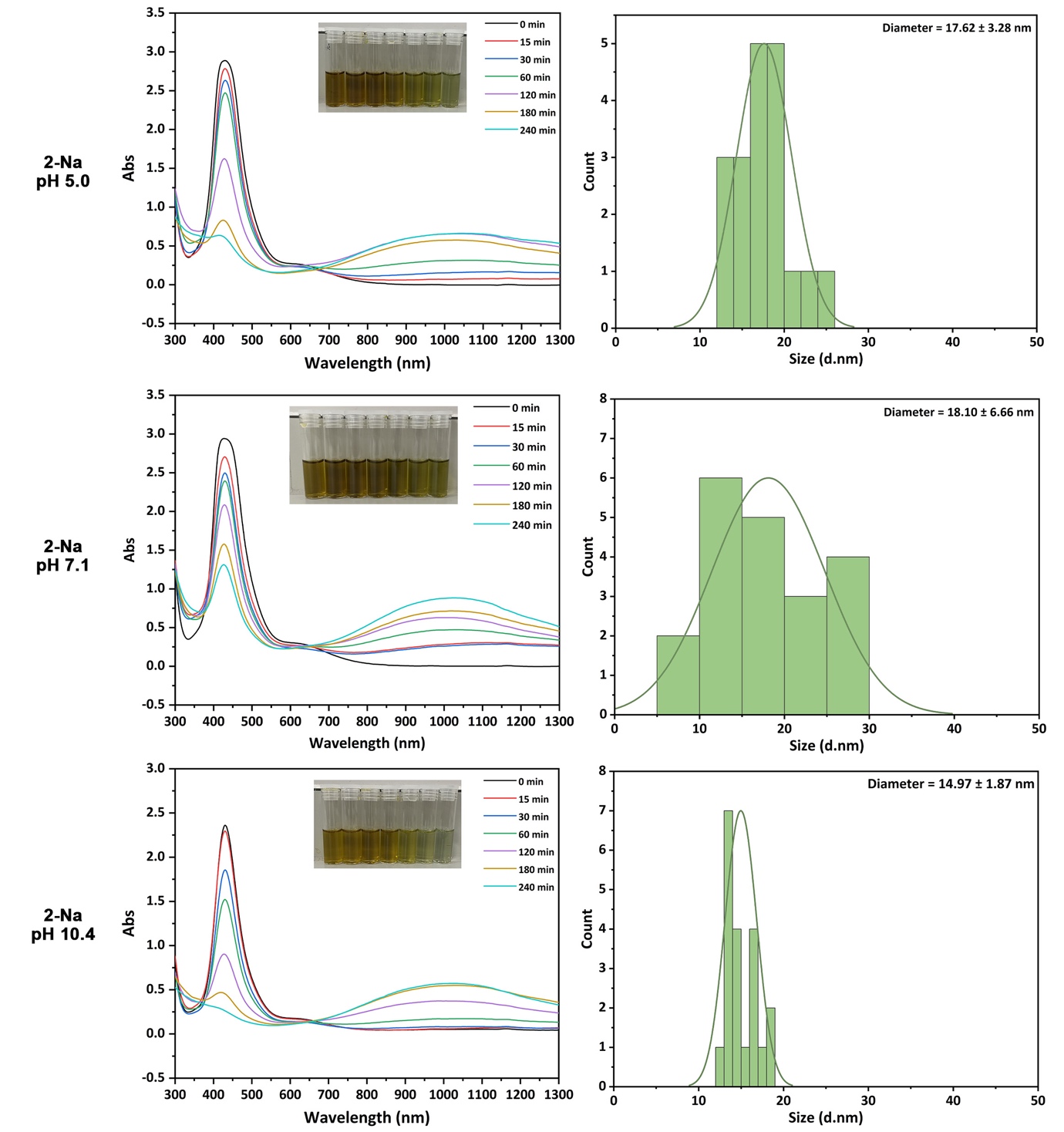


## **Figure S34.** Decomposition studies of **2** at different pH: UV-Vis-NIR spectra and size distribution (90 ℃)


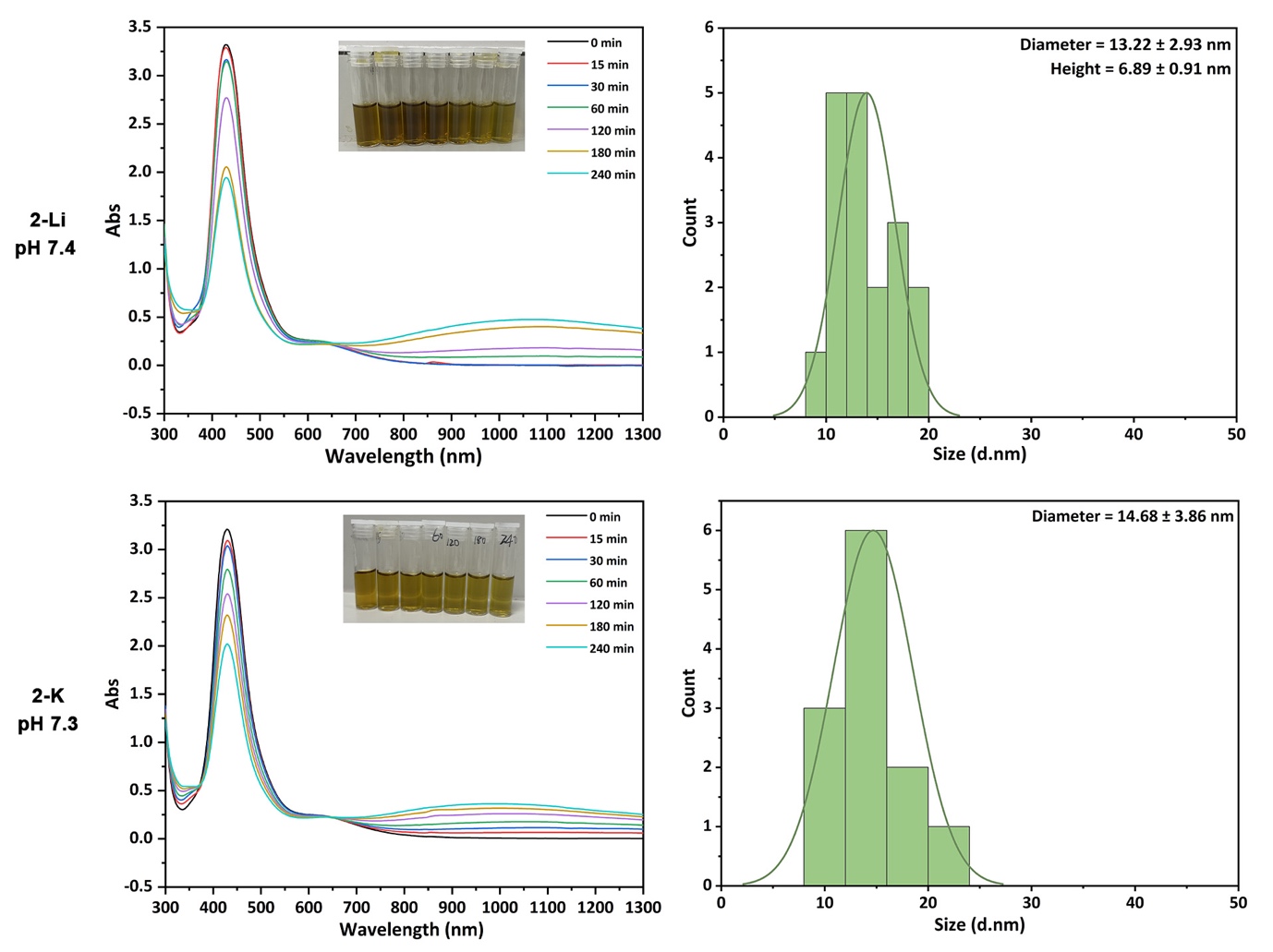


## **Figure S35.** Decomposition studies of **2** with different cations: UV-Vis-NIR spectra and size distribution (90 ℃)


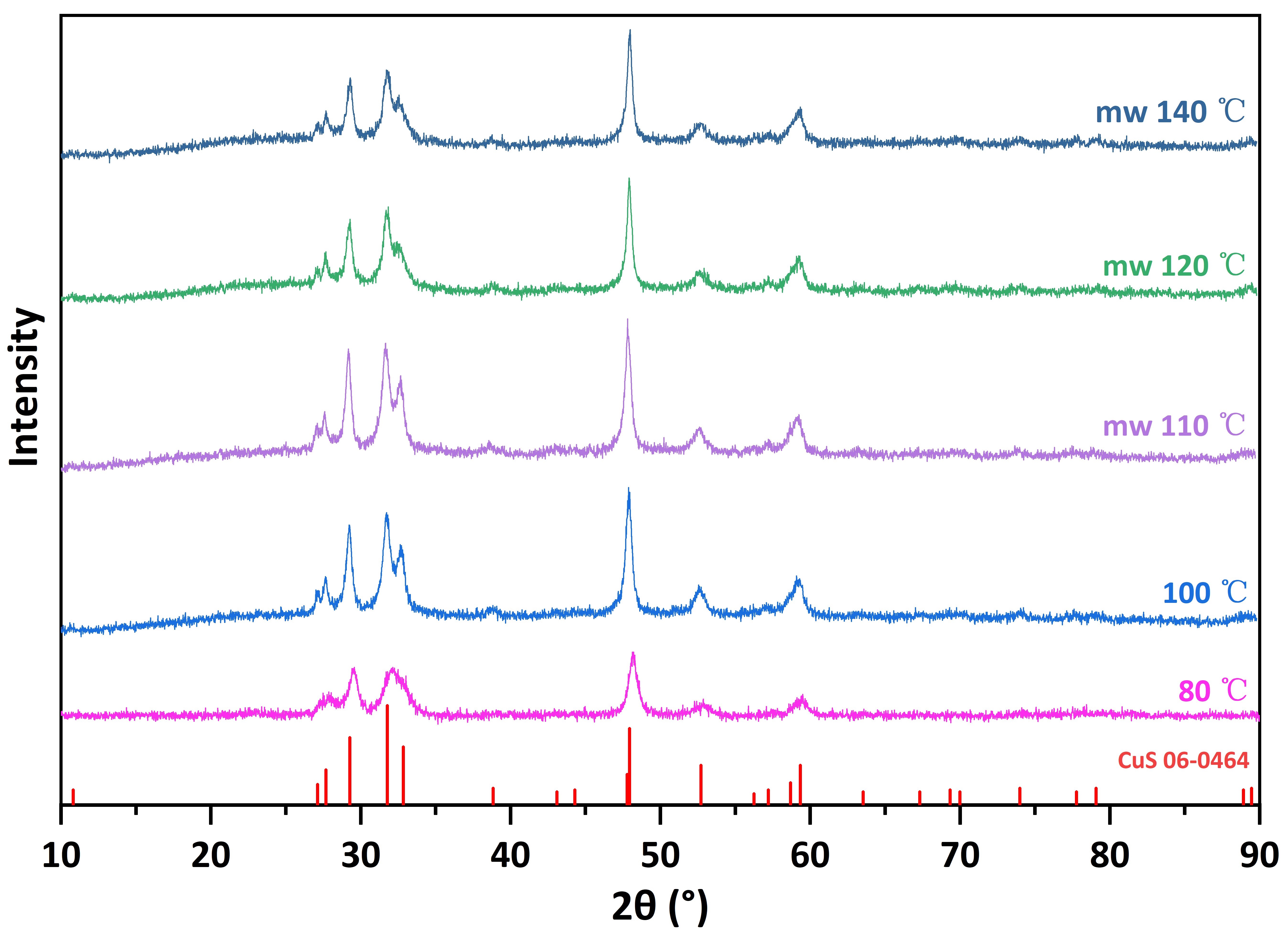


## **Figure S36.** PXRD patterns of NPs obtained from **1-Na** at different temperatures. Microwave-assisted decompositions were abbreviated as “mw”


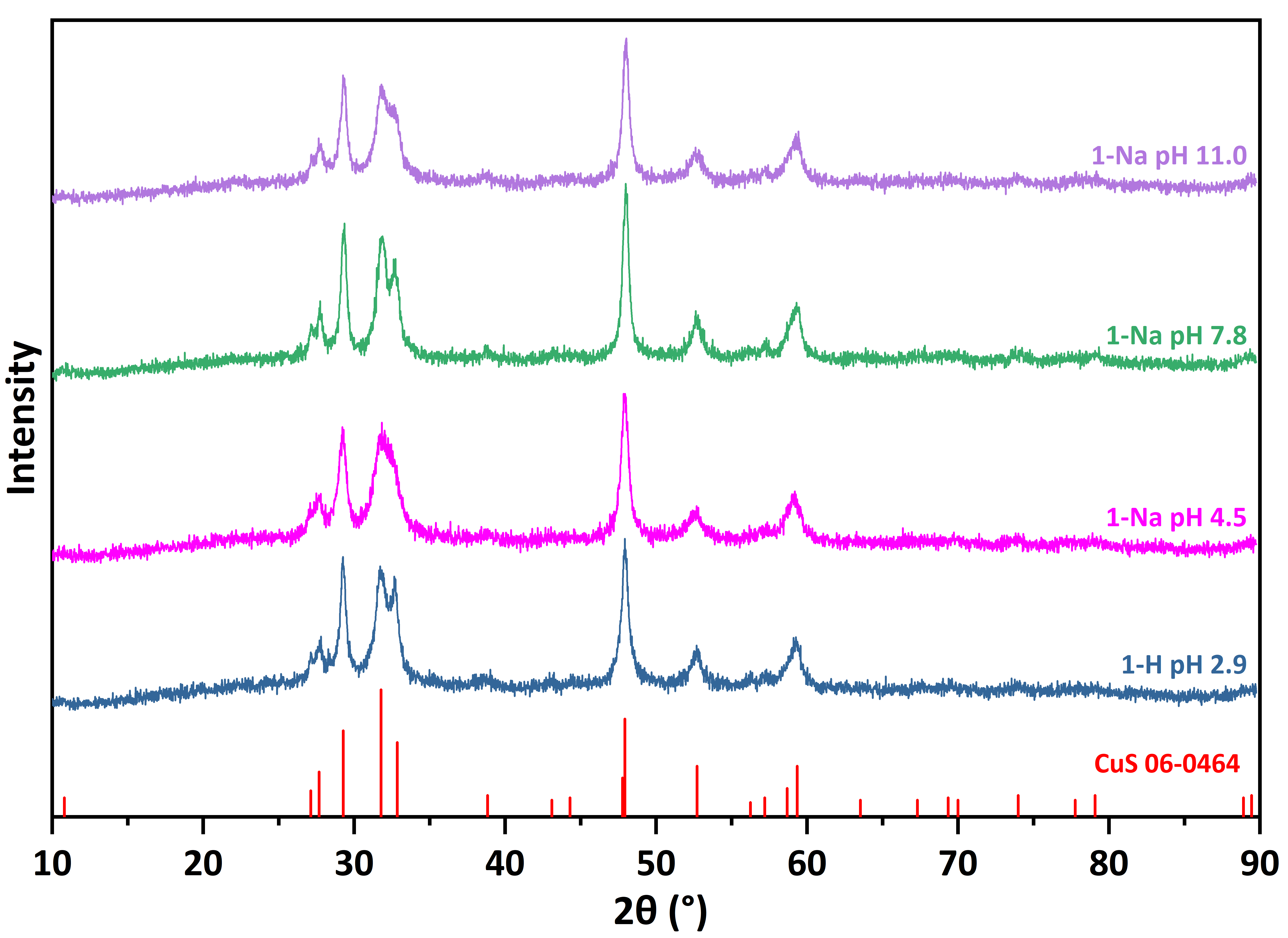


## **Figure S37.** PXRD patterns of NPs obtained from **1** at different pH (90 ℃)


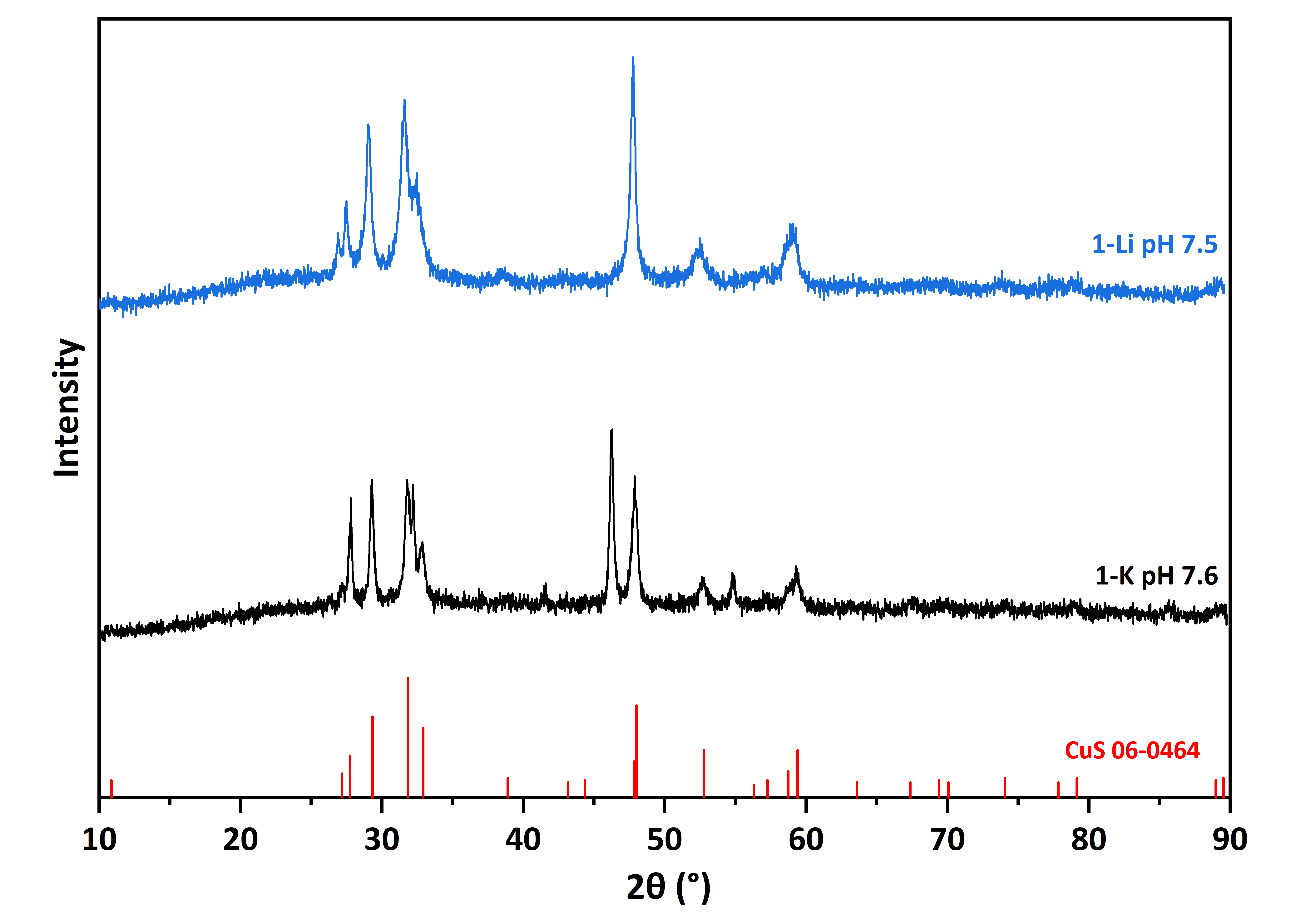


## **Figure S38.** PXRD patterns of NPs obtained from **1** with different alkali cations (90 ℃)


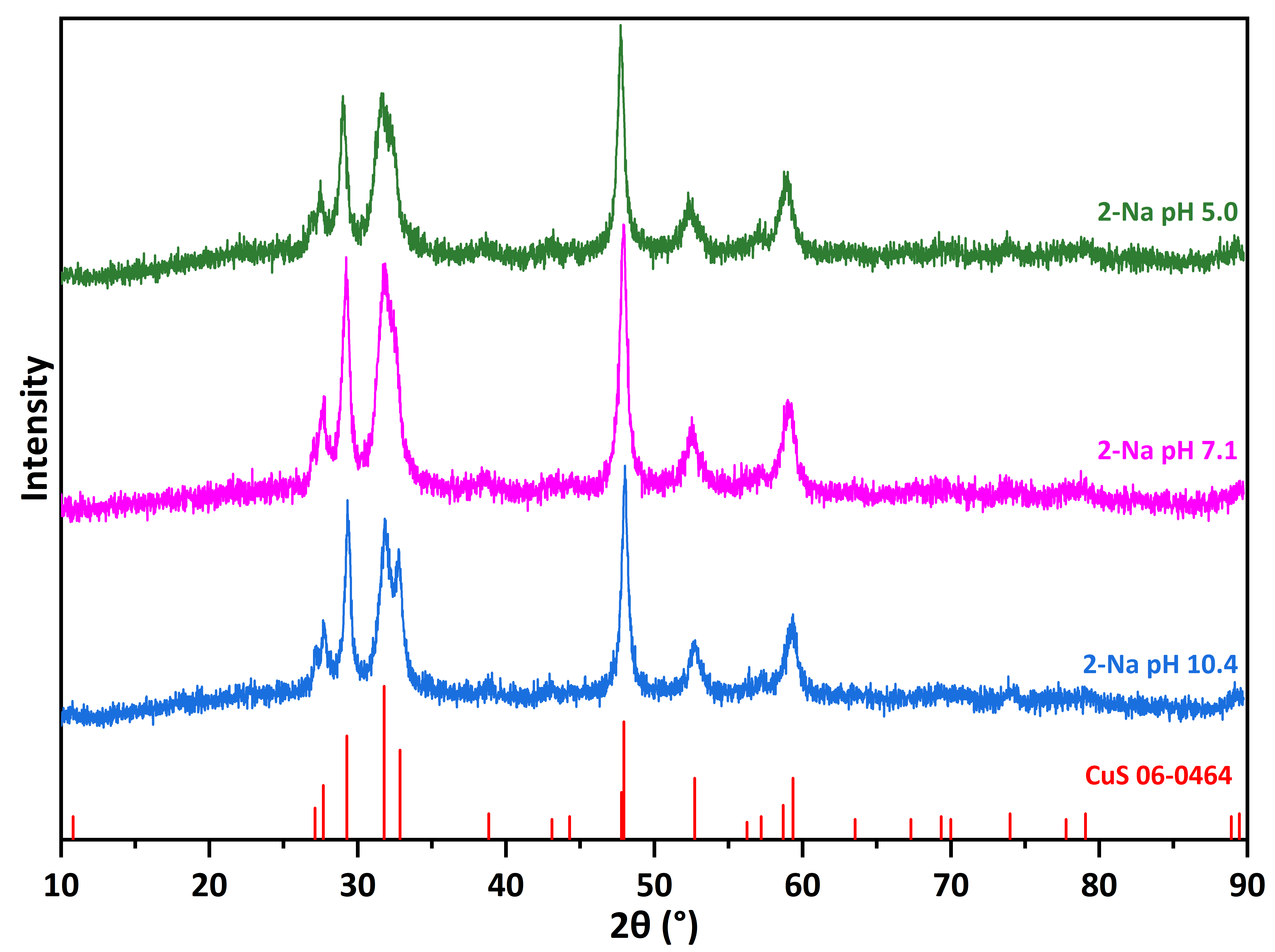


## **Figure S39.** PXRD patterns of NPs obtained from **2** at different pH (90 ℃)


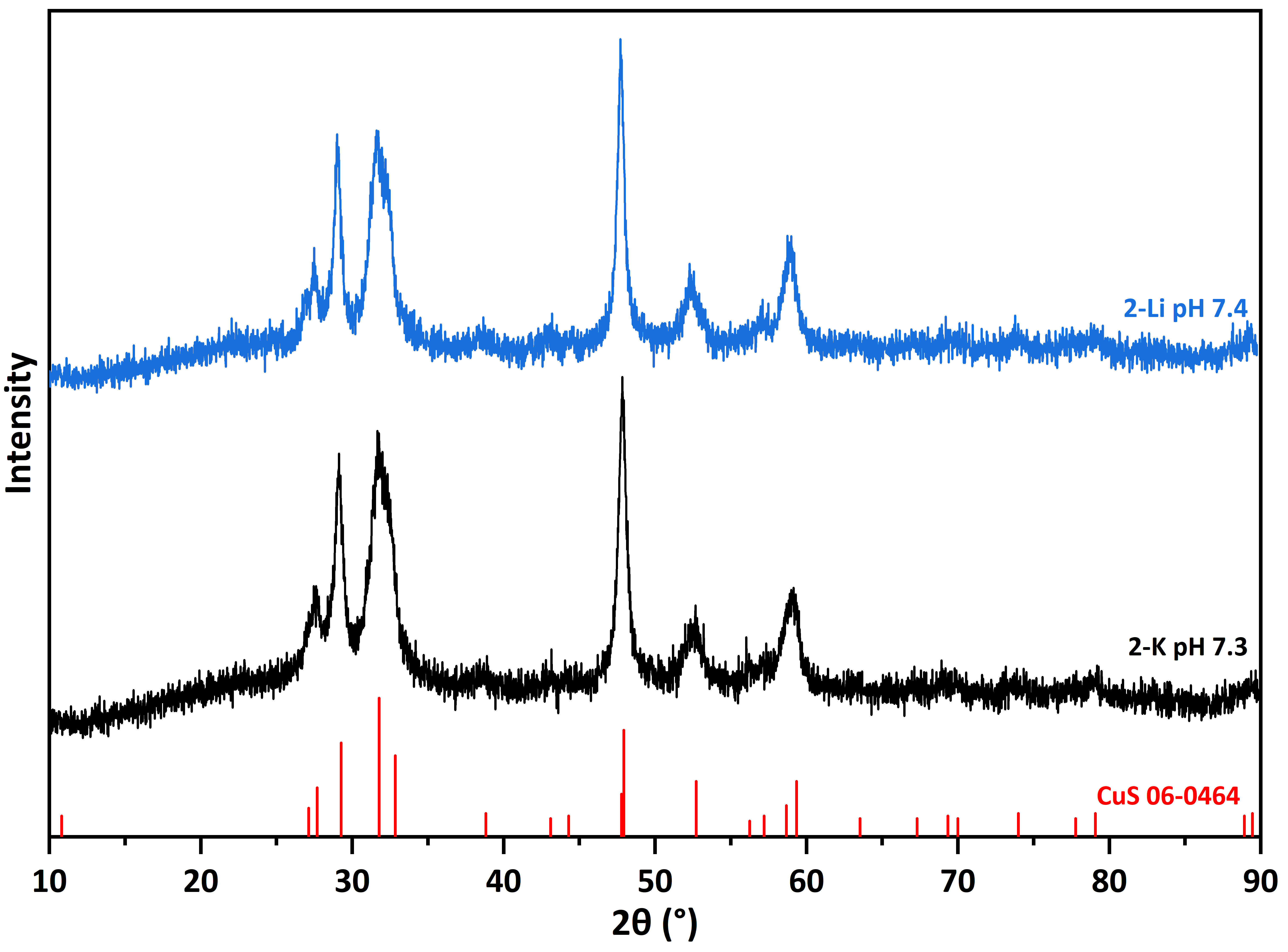


## **Figure S40.** PXRD patterns of NPs obtained from **2** with different cations (90 ℃)


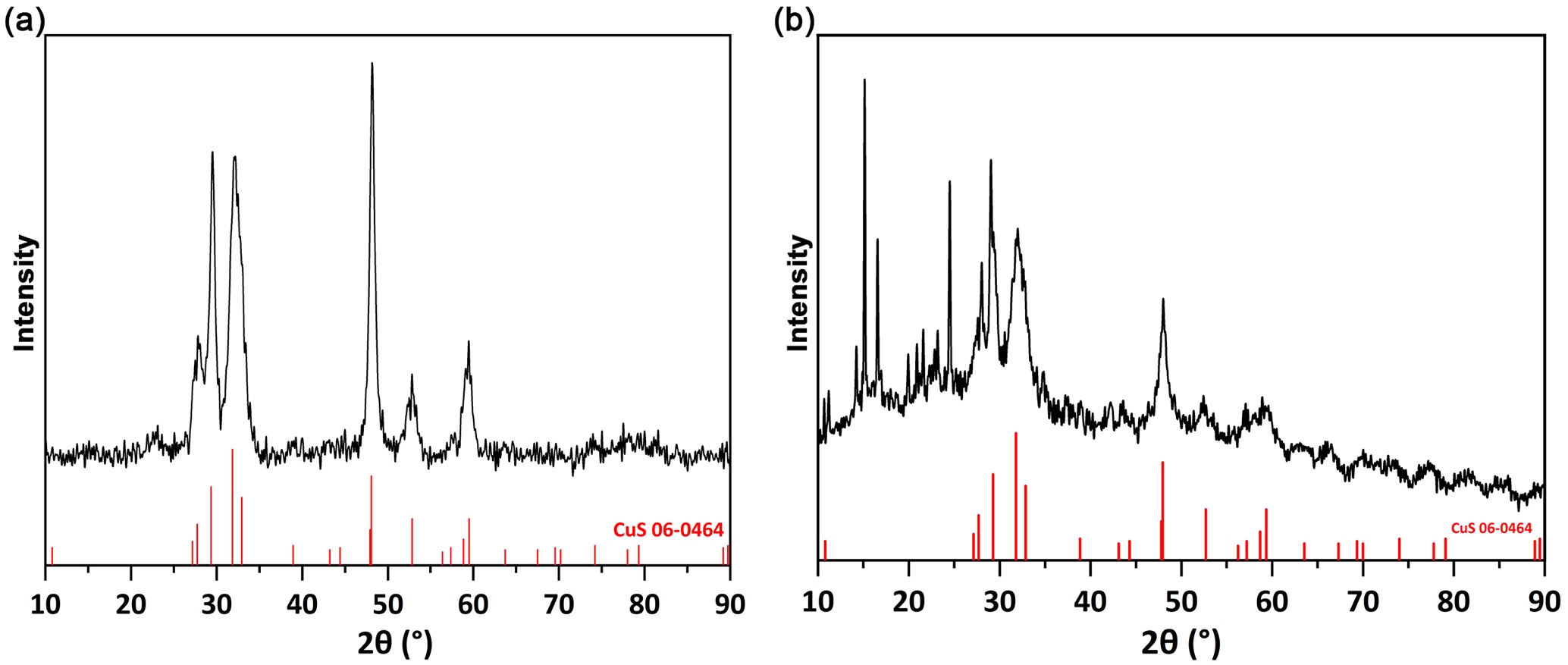


## **Figure S41.** PXRD patterns of NPs from decomposing (a) **4** and (b) **5** at 90 ℃. CuS (Covellite, JCPDS No. 06-0464) is used as a reference

## **Table S1.** Selected IR band positions ν (in cm^-1^) and the decomposition temperatures for DTCs and Cu-DTC precursors

| Compound | *ν*(C-S) | *ν*(COO) | T_dec_  (℃) |
| --- | --- | --- | --- |
| Imd-Na-DTC | 1207 | 1595 | - |
| **1-Na** | 1213 | 1592 | 301 |
| **1-H** | 1203 | 1724, 1687, 1643 | 179 |
| Sar-Na-DTC | 1193 | 1589 | - |
| **2-Na** | 1213 | 1610 | 250 |
| **2-H** | 1207 | 1755, 1707 | 187 |
| **3** | 1215 | - | - |
| **4** | 1206 | 1712 | 174 |
| **5** | 1195 | 1726 | 162 |

## **Table S2.** Magnetic susceptibility table of Cu-DTC complexes

| **Sample** | **R_0_(×1)** | **R_0_(×10)** | **R** | **L**  **(cm)** | **M_0_**  **(g)** | **M_1_**  **(g)** | ***Χ*_ρ_**  **(cm^3^/g)** | **T (K)** | **M_W_**  **(g/mol)** | ***χ*_m_**  **(cm^3^/mol)** | **Correction (×10^-6^ cm^3^/mol)** | ***χ*_a_**  **(cm^3^/mol)** | **μ_eff_**  **(Bohr magneton)** | **UPES** | **Valence** |
| --- | --- | --- | --- | --- | --- | --- | --- | --- | --- | --- | --- | --- | --- | --- | --- |
| **1-Na** | -040 | -004 | 043 | 2.0 | 0.8350 | 0.9226 | 1.89498E-06 | 293 | 456 | 0.000864 | -225.06 | 0.000639 | 1.22 | 1 | 2 |
| **2-Na** | -040 | -004 | 032 | 1.7 | 0.8302 | 0.9081 | 1.57125E-06 | 293 | 368 | 0.000578 | -189.18 | 0.000389 | 0.95 | 1 | 2 |
| **1-H** | -036 | -003 | 028 | 1.5 | 0.8314 | 0.8873 | 1.71735E-06 | 295 | 480 | 0.000824 | -164.84 | 0.000659 | 1.25 | 1 | 2 |
| **2-H** | -029 | -002 | 007 | 1.6 | 0.8110 | 0.8804 | 8.29971E-07 | 295 | 391 | 0.000325 | -157.44 | 0.000167 | 0.63 | 0~1 | Mixture |
| **4** | -023 | -002 | -20 | 0.8 | 0.8131 | 0.8503 | 6.45161E-08 | 295 | 227 | 1.46E-05 | -85.22 | -0.000057 | - | 0 | 1 |
| **5** | -034 | -003 | -043 | 1.5 | 0.8318 | 0.8664 | -3.90173E-07 | 295 | 271 | -0.000106 | -87.92 | -0.000071 | - | 0 | 1 |

(**R_0_**: readings for the empty tube; **R**: readings for the tube with sample; **L**: length of the sample in the tube; **M_0_**: mass of the empty tube; **M_1_**: mass of the tube with sample; ***Χ*_ρ_**: mass susceptibility; **T**: temperature; **Mw**: molecular weight; ***χ*_m_**: molar susceptibility; ***χ*_a_**: corrected molar susceptibility; **μ_eff_**: the effective magnetic moment; **UPES**: unpaired electrons;)

## **Appendix**

TEM images used to measure the size distribution: size (diameter) was measured in the diagonal distance

- 1-H (**pH 2.9**)


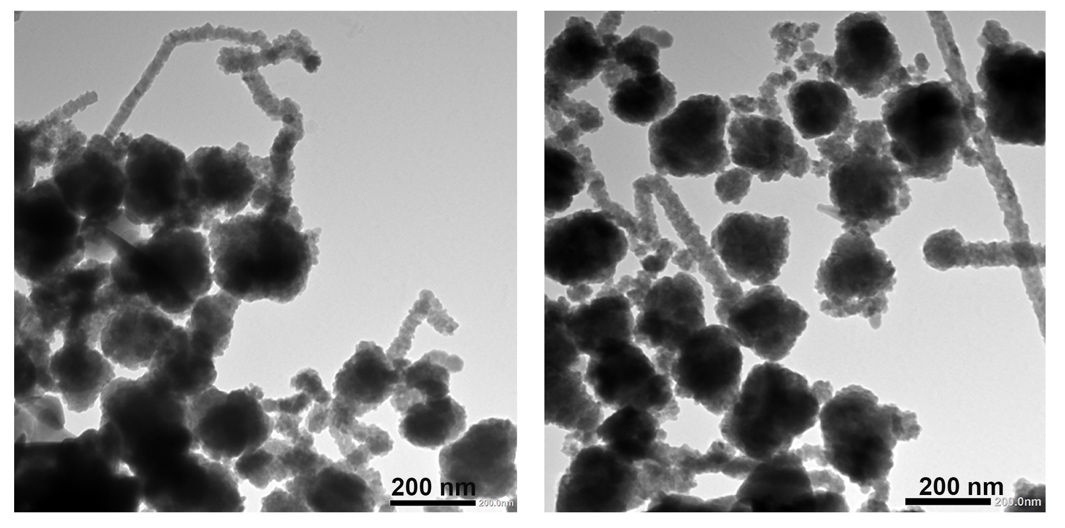


- 1-Na (**pH 4.5**)


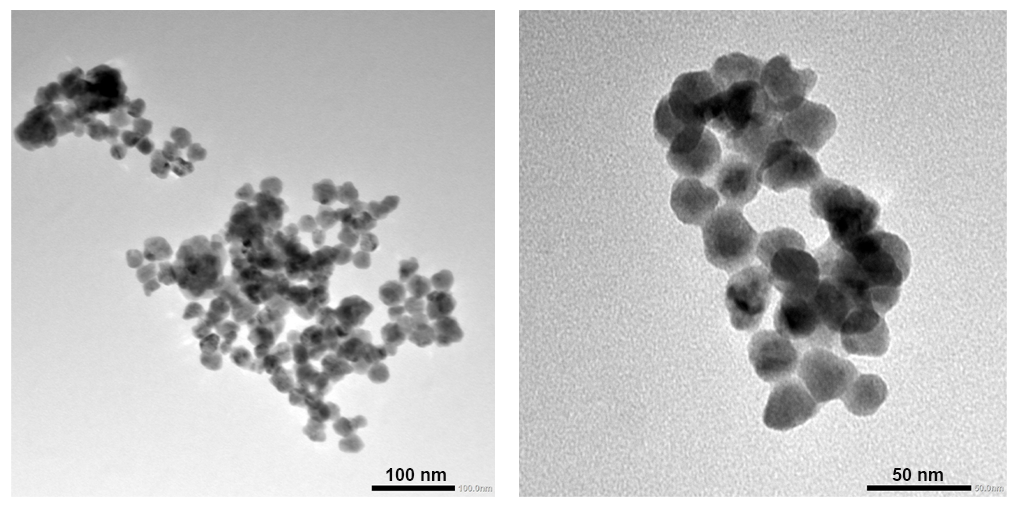


- 1-Na (**pH 7.8**)


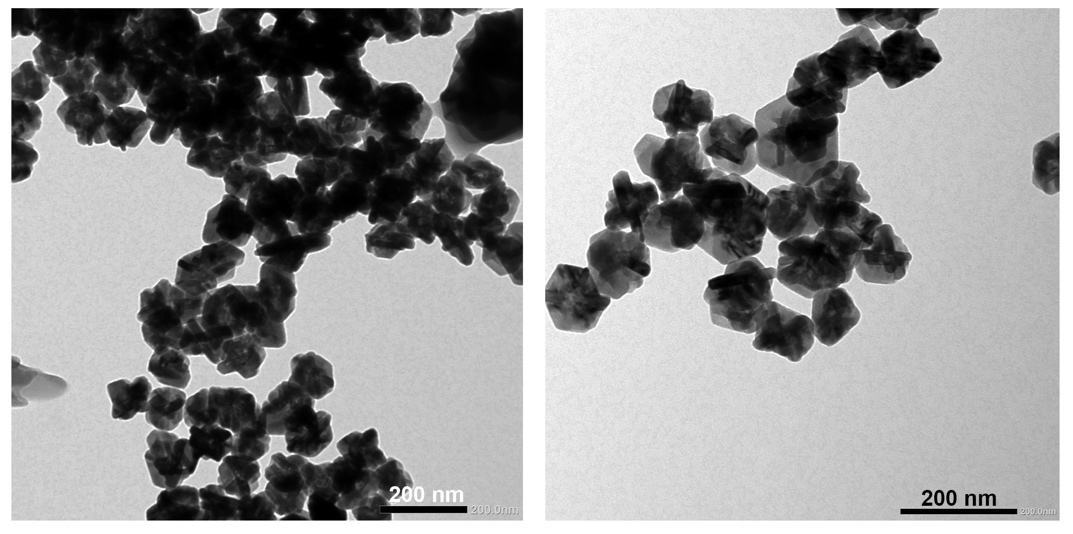


- 1-Na (**pH 11.0**)


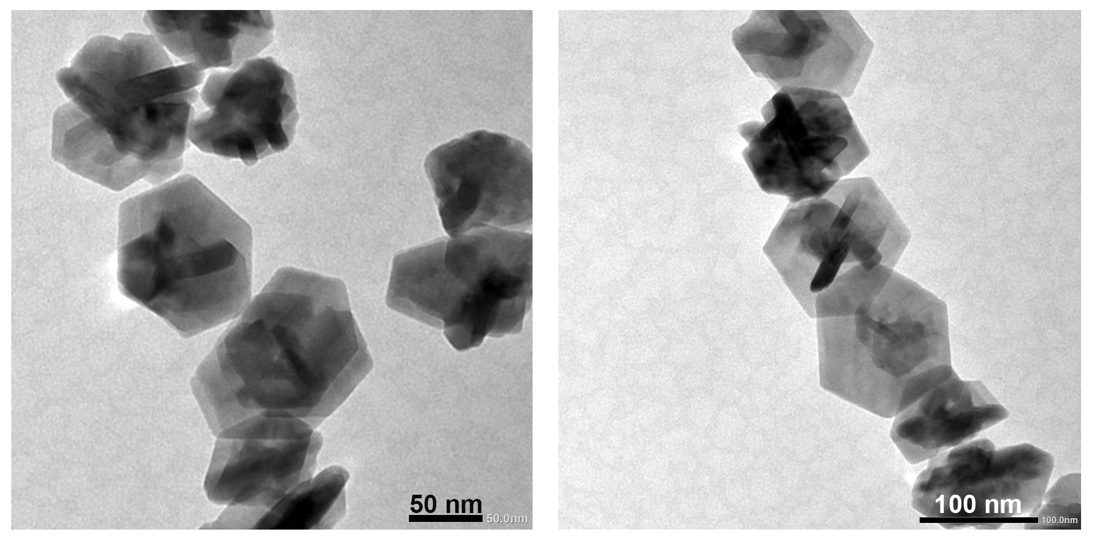


- 1-**Li** (pH 7.5)


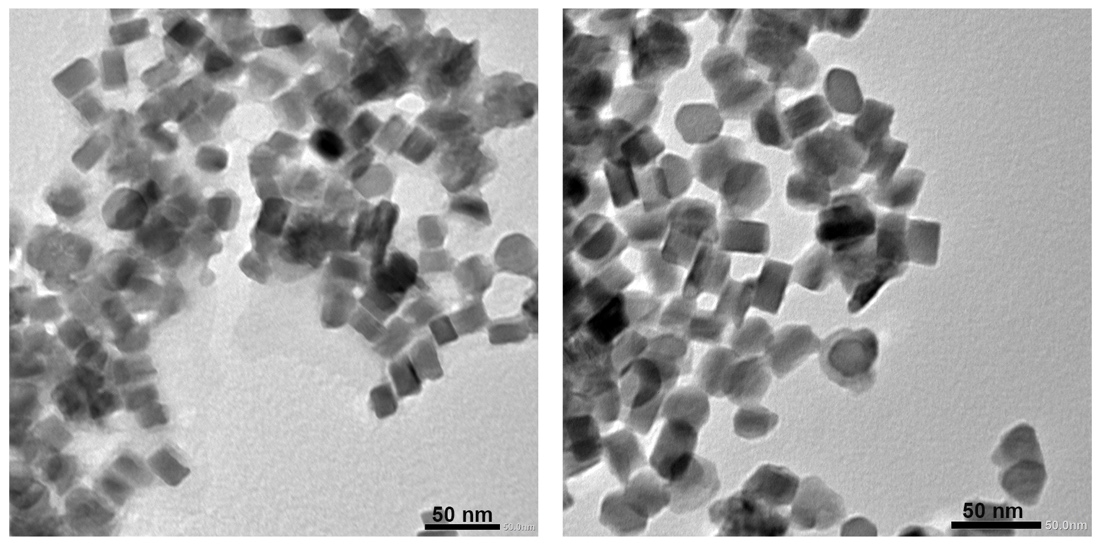


- 1-**K** (pH 7.6)


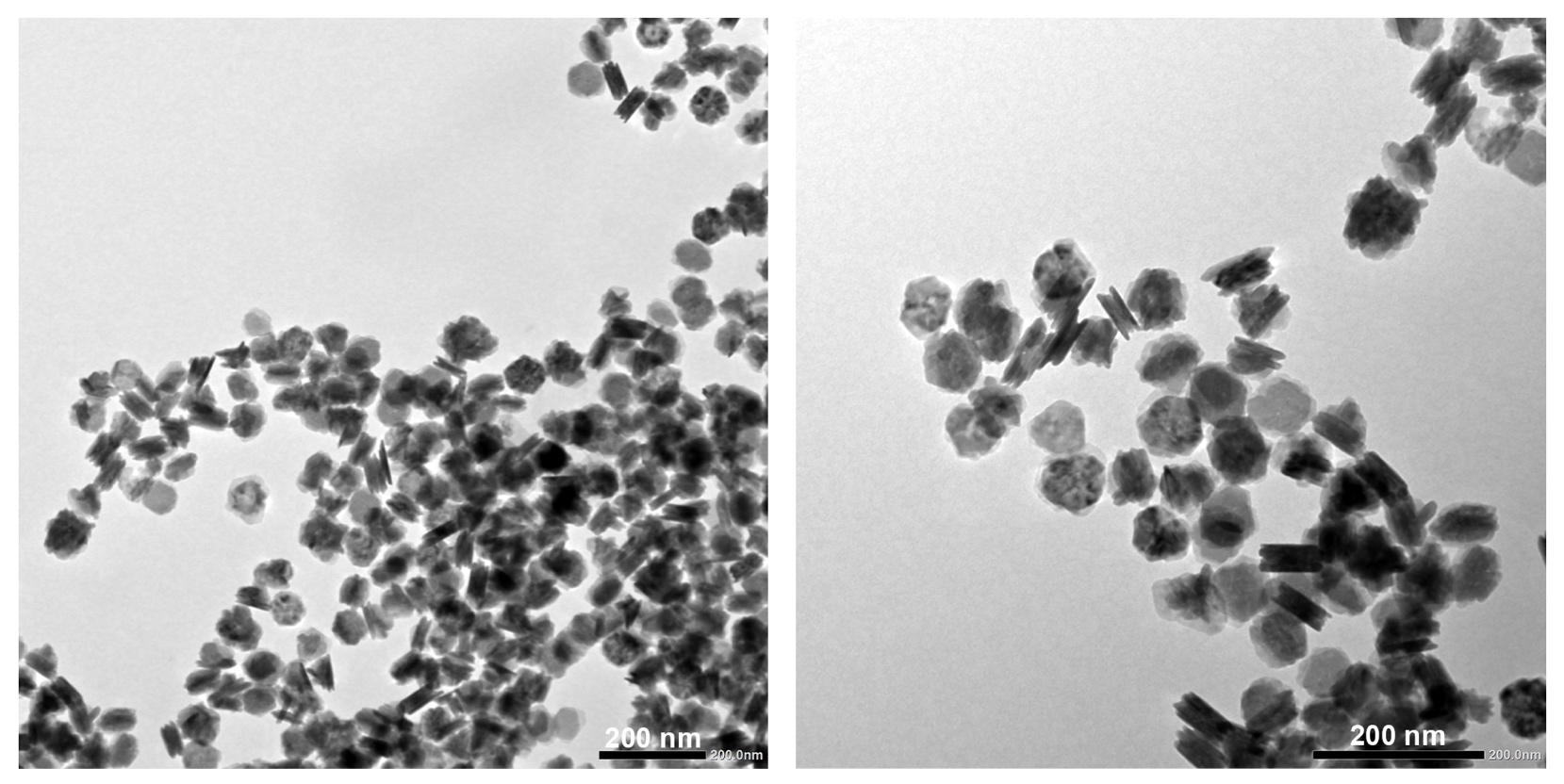


- **4**


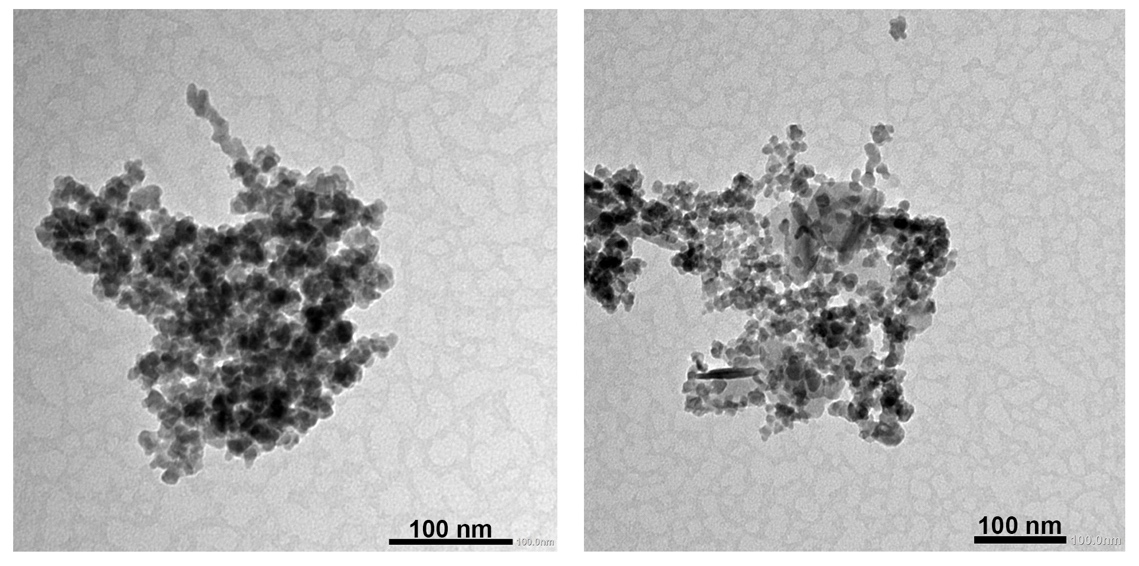


- 1-**Na** (pH 7.8), hot-injection at **80 ℃**


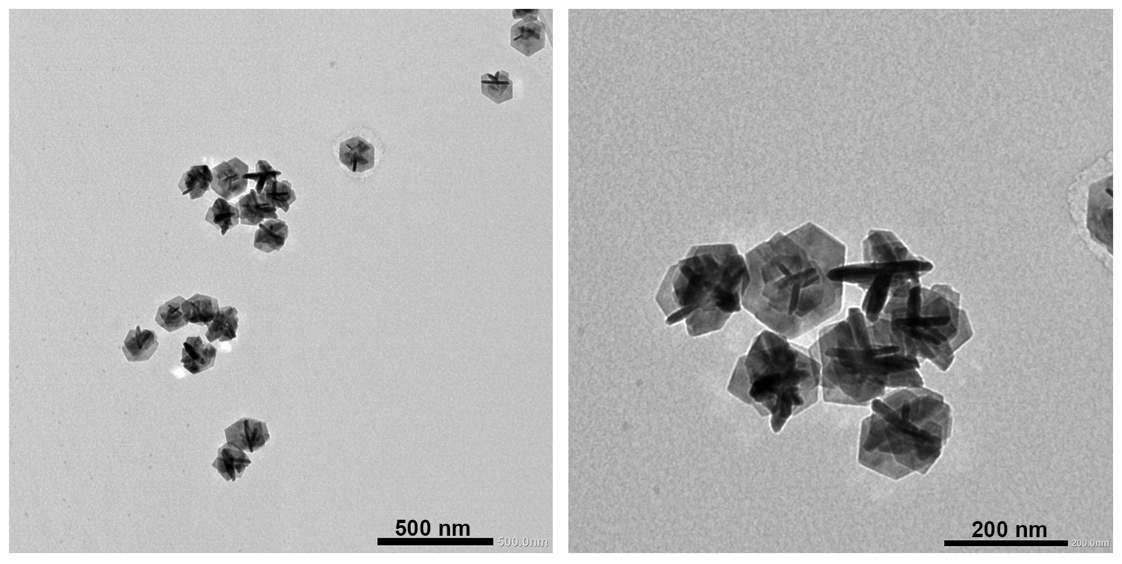


- 1-**Na** (pH 7.8), hot-injection at **100 ℃**


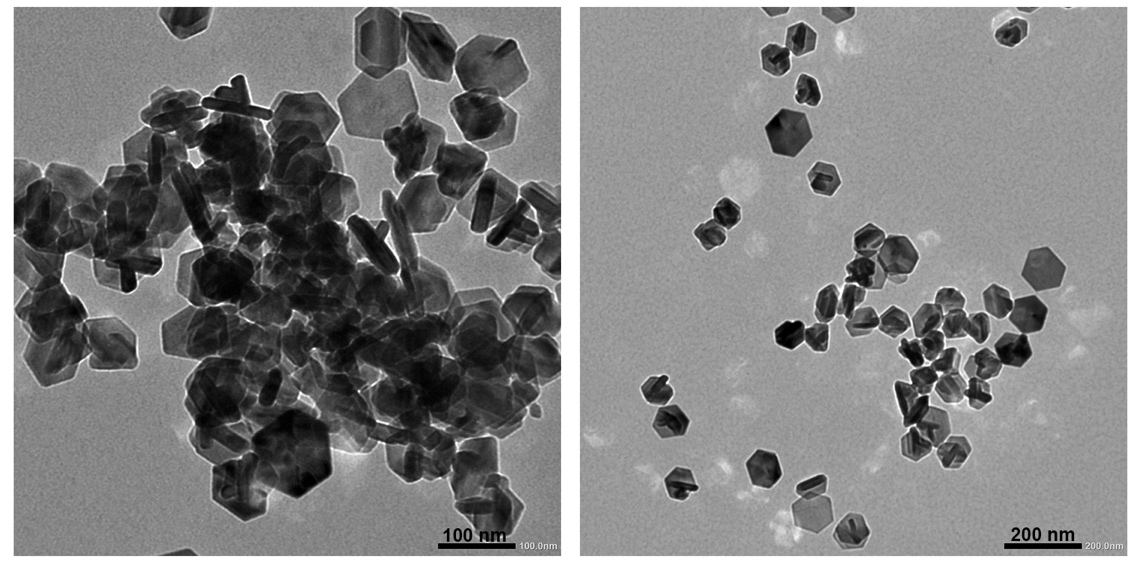


- 1-**Na** (pH 7.8), **microwave****-assisted** at **110 ℃**


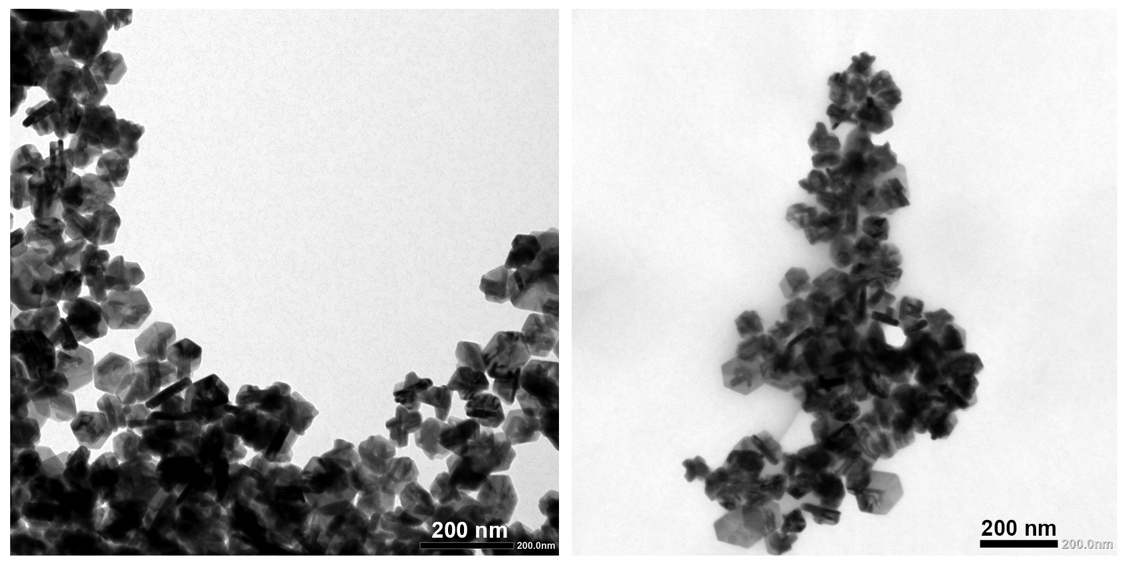


- 1-**Na** (pH 7.8), **microwave-assisted** at **120 ℃**


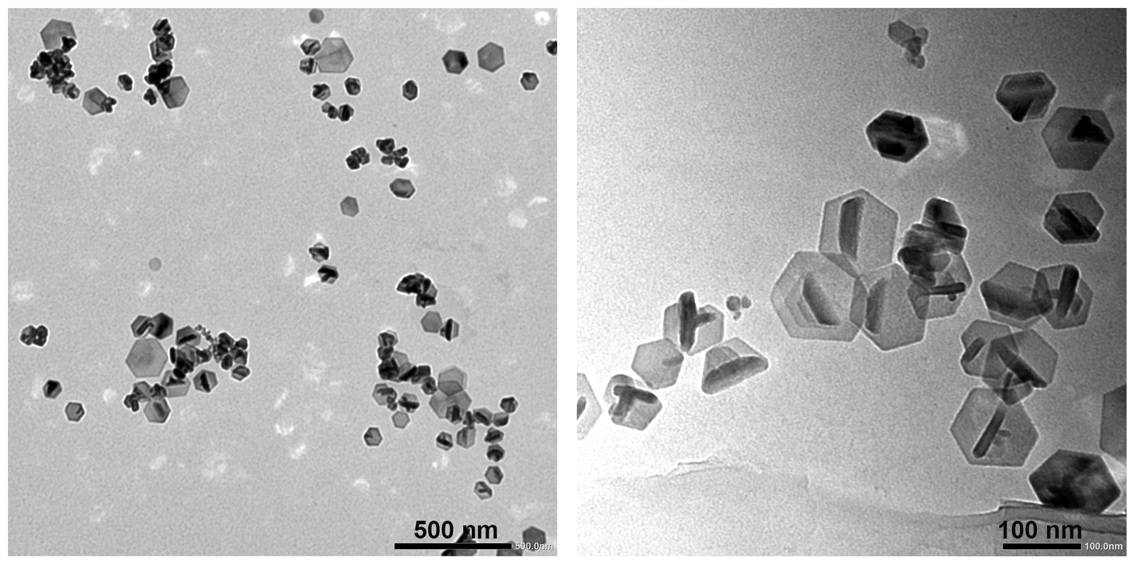


- 1-**Na** (pH 7.8), **microwave-assisted** at **140 ℃**


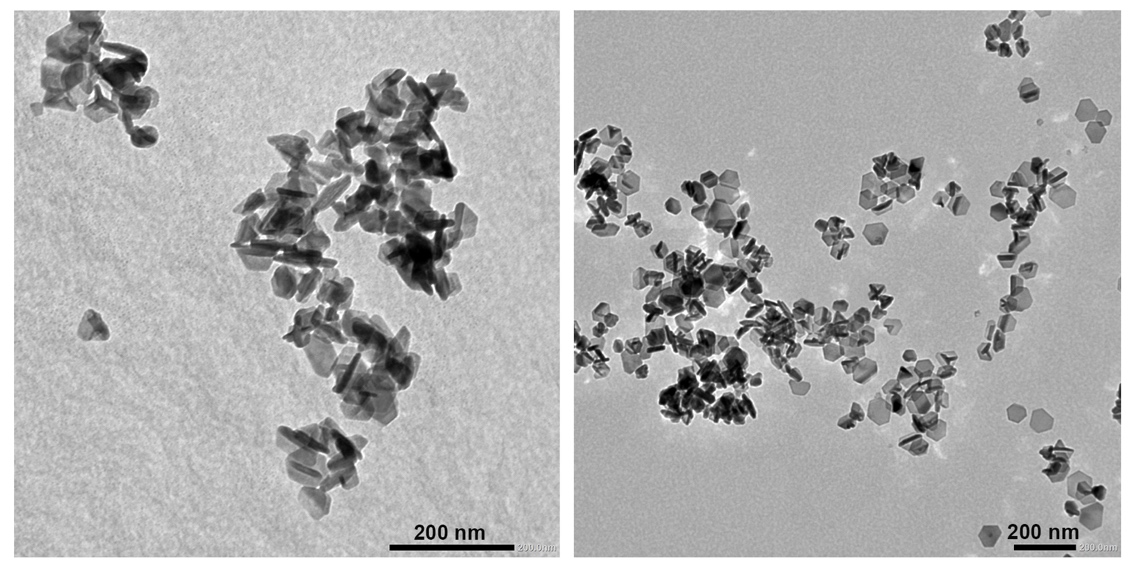


- 2-**Na** (**pH 5.0**)


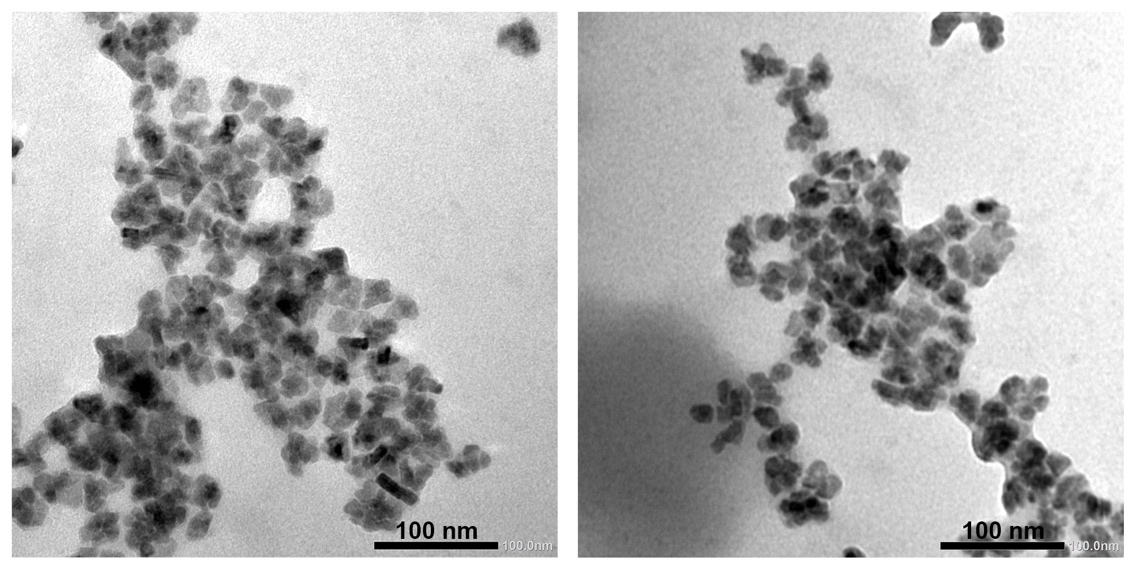


- 2-**Na** (**pH 7.1**)


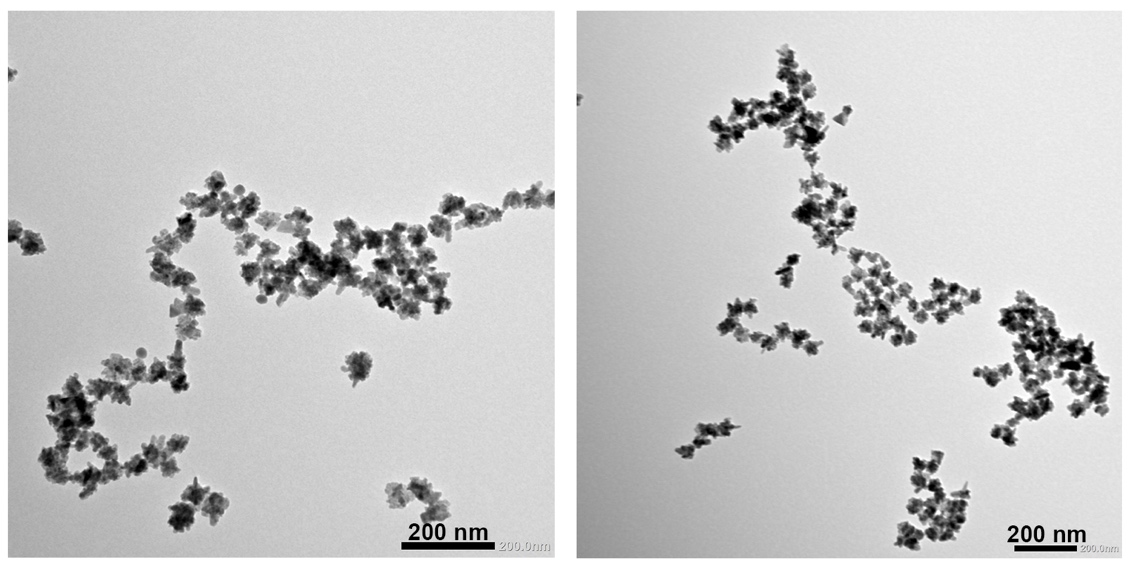


- 2-**Na** (**pH 10.4**)


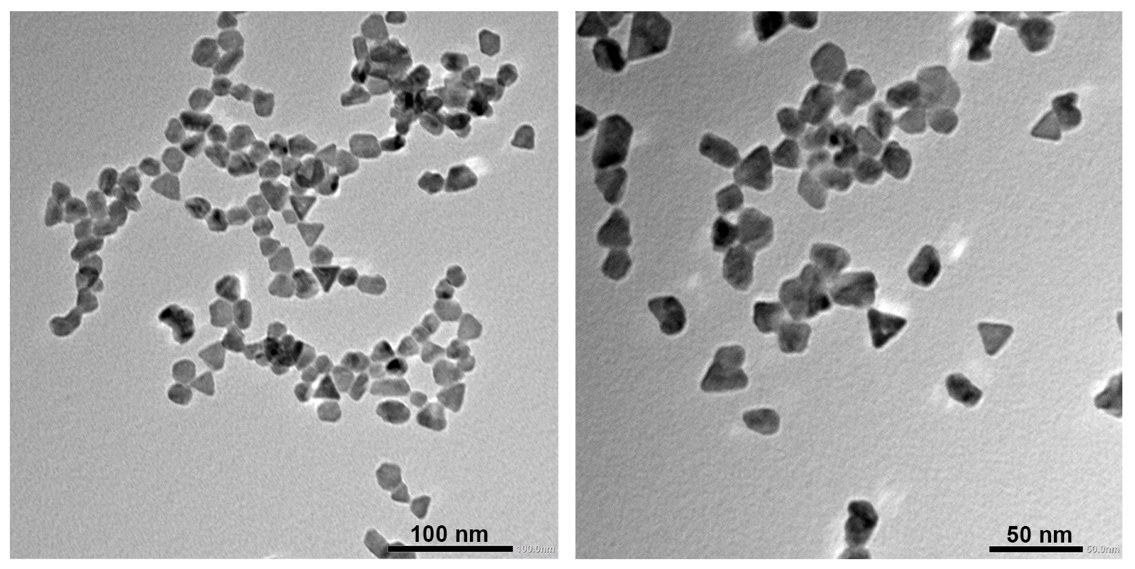


- 2-**Li** (pH 7.4)


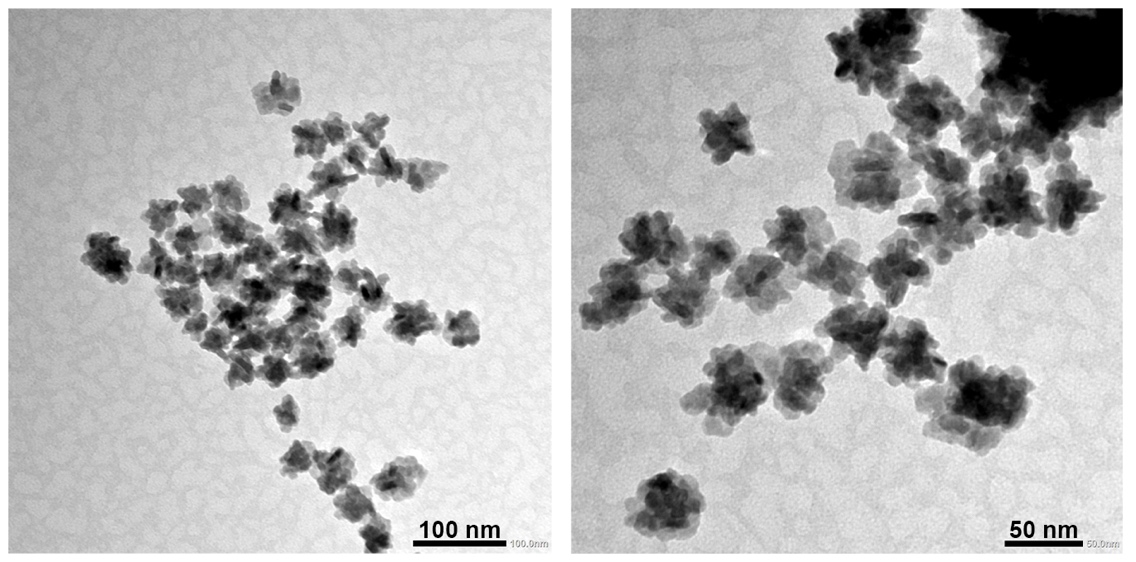


- 2-**K** (pH 7.3)


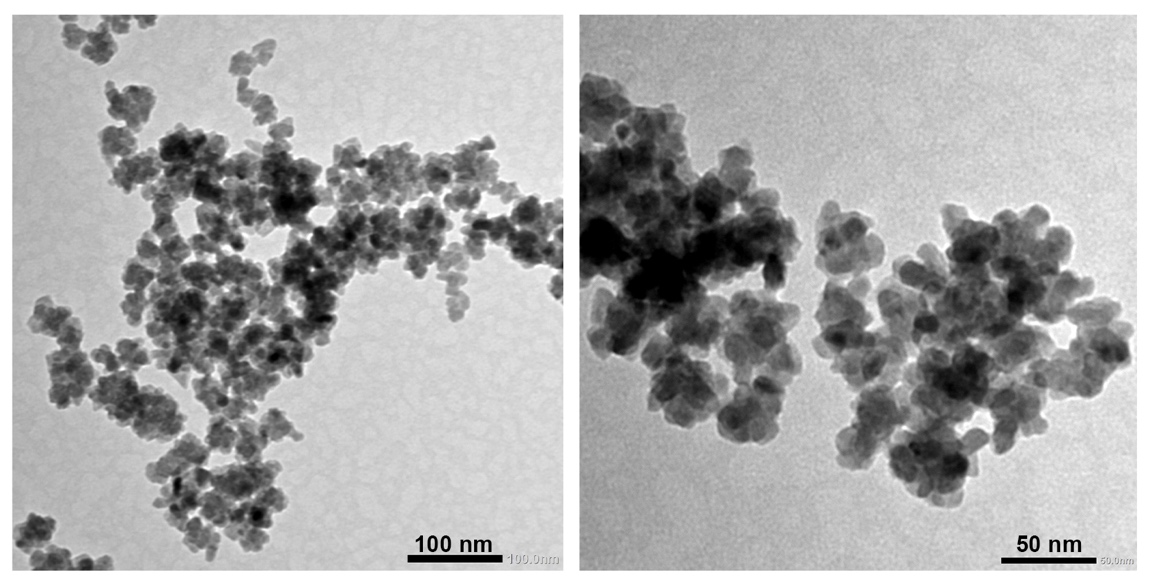


- **5**


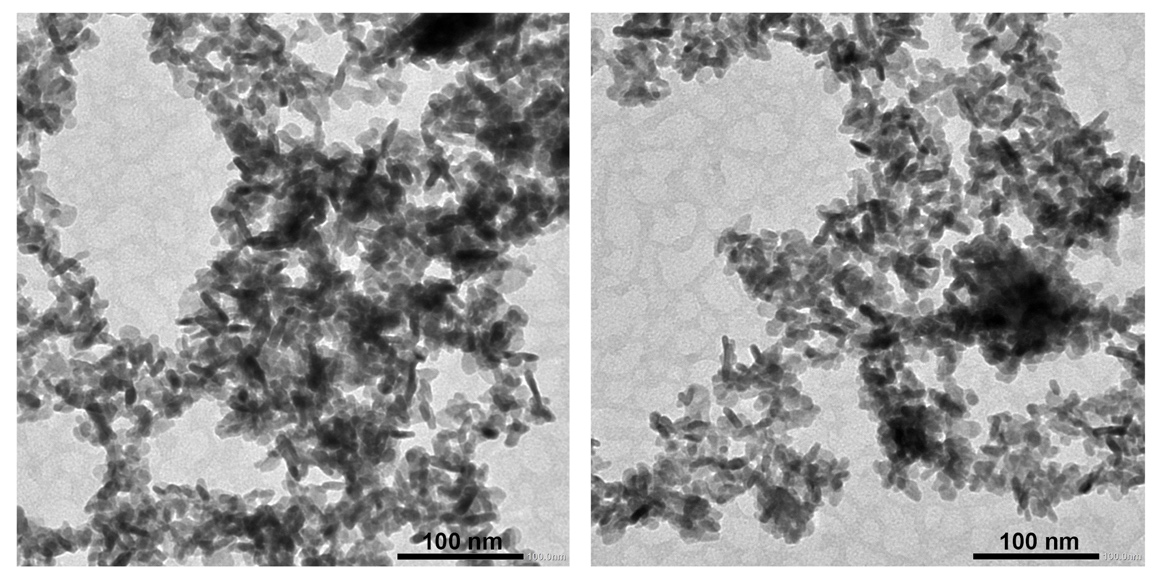

Supplement: Supplementary file 1 — Supporting File: smll73141‐sup‐0001‐SuppMat.docx. [file SMLL-22-e14339-s001.docx]
